# Identifying ‘win-win-win’ futures from inequitable value chain trade-offs: A system dynamics approach

**DOI:** 10.1016/j.agsy.2021.103096

**Published:** 2021-05

**Authors:** Gregory S. Cooper, Karl M. Rich, Bhavani Shankar, Vinay Rana, Nazmun N. Ratna, Suneetha Kadiyala, Mohammad J. Alam, Sharan B. Nadagouda

**Affiliations:** a Centre for Development, Environment and Policy (CeDEP), School of Oriental and African Studies (SOAS), London, United Kingdom; b International Livestock Research Institute (ILRI), West Africa Regional Office, Dakar, Senegal; c Institute for Sustainable Food, University of Sheffield, Sheffield, United Kingdom; d Transform Rural India Foundation (TRIF), Raipur, Chhattisgarh, India; e Department of Global Value Chain & Trade, Faculty of Agribusiness and Commerce, Lincoln University, Christchurch, New Zealand; f Department for Population Health, London School of Hygiene and Tropical Medicine (LSHTM), London, United Kingdom; g Department of Agribusiness and Marketing, Bangladesh Agricultural University (BAU), Mymensingh, Bangladesh; h Digital Green, East India Office, Patna, India

**Keywords:** System dynamics model, Agricultural marketing, Fruits and vegetables, Plausible futures, India

## Abstract

**Context:**

There is growing recognition that food systems must adapt to become more sustainable and equitable. Consequently, in developing country contexts, there is increasing momentum away from traditional producer-facing value chain upgrades towards efforts to increase both the availability and affordability of nutritious foods at the consumer level. However, such goals must navigate the inherent complexities of agricultural value chains, which involve multiple interactions, feedbacks and unintended consequences, including important but often surprising trade-offs between producers and consumers.

**Objective and methods:**

Based around the 'Loop' horticultural aggregation scheme of Digital Green in Bihar, India, we develop a system dynamics modelling framework to survey the value chain trade-offs emerging from upgrades that aim to improve the availability of fruits and vegetables in small retail-oriented markets. We model the processes of horticultural production, aggregation, marketing, and retailing – searching for futures that are ‘win-win-win’ for: (i) the availability of fruits and vegetables in small retail markets, (ii) the profits of farmers participating in aggregation, and (iii) the sustainability of the initial scheme for Digital Green as an organisation. We simulate two internal upgrades to aggregation and two upgrades to the wider enabling environment through a series of 5000 Monte Carlo trajectories – designed to explore the plausible future dynamics of the three outcome dimensions relative to the baseline.

**Results:**

We find that ‘win-win-win’ futures cannot be achieved by internal changes to the aggregation scheme alone, emerging under a narrow range of scenarios that boost supplies to the small retail market whilst simultaneously supporting the financial takeaways of farmers. In contrast, undesirable producer versus consumer trade-offs emerge as unintended consequences of scaling-up aggregation and the introduction of market-based cold storage.

**Significance:**

This approach furthers ongoing efforts to capture complex value chain processes, outcomes and upgrades within system dynamics modelling frameworks, before scanning the horizon of plausible external scenarios, internal dynamics and unintended trade-offs to identify ‘win-win-win’ futures for all.

## Highlights

• It is increasingly acknowledged that food system interventions must benefit both producers and consumers
• We develop a systems tool to explore trade-offs emerging from adapting value chain interventions to become more consumer-sensitive
• Futures with improved local vegetable delivery, producer profits and intervention costs are traced to their causal dynamics
• ‘Win-win-wins’ require improved local retail demands to attract farmers away from urban markets without weakening profits
• Systems-based approaches can horizon-scan plausible futures that navigate complex trade-offs in agricultural value chains

## Graphical abstract

**Figure:**
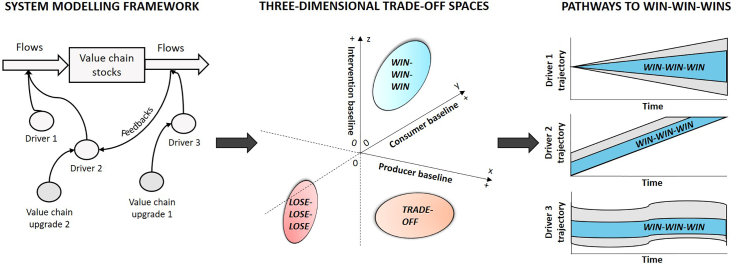

## 1 Introduction

It is increasingly acknowledged that aspirational futures, such as the United Nations' second Sustainable Development Goal to end all forms of malnutrition by 2030, may only be achieved by providing nutritious diets in an equitable and sustainable manner (Dangour et al., 2017; FAO, 2014). However, horticultural value chains, which supply fruits and vegetables (F&V) vital in the combat of chronic diseases and micronutrient deficiencies (Afshin et al., 2019), are frequently characterised by seasonal and perishable supplies, erratic prices, outdated distribution systems and insufficient awareness around the importance of F&V consumption (Maestre et al., 2017). With only 18% of individuals in low and medium income countries consuming the World Health Organisation's (WHO) recommended 400 g/day of F&V (Frank et al., 2019), there is growing recognition around the importance of intervening in value chains and their markets to improve connectivity between producers and consumers (Elliot et al., 2008; Gillespie et al., 2019).

In India, where average F&V intake is estimated to be 200 g/capita/day (Choudhury et al., 2020), the generation of fair outcomes for all actors in horticultural value chains faces numerous challenges (Kadiyala et al., 2012). Entry costs have excluded smallholder farmers from value chains during the expansion of cold storage (Minten et al., 2011), the strengthening of food safety through labelling (Patil et al., 2016) and efforts to modernise marketing chains (Trebbin and Franz, 2010). Moreover, despite F&V production tripling since the mid-1990s (Varadharajan et al., 2013), retail prices have not responded proportionately (Rahman, 2012) and wastage rates between farm and fork remain 30–40% of total production (Minocha et al., 2018).

At the regional scale, farmers often prefer to supply higher demand urban markets due to the relative range and financial wealth of buyers, and the comparatively developed infrastructures that help to minimise losses, preserve F&V quality and increase price transparency (Choudhury et al., 2020; Pingali et al., 2019). Symptomatic of the above, consumption in rural areas is estimated to average only 160 g/capita/day (Minocha et al., 2018). Therefore, pathways towards ‘win-win’ futures that simultaneously achieve positive outcomes for the financial takeaways of producers, and the availability and affordability of F&V for consumers, must be sensitive to the complex dynamics that transmit benefits and trade-offs across value chains (Hawkes and Ruel, 2011; Klapwijk et al., 2014).

Based around this central premise, our study has three main aims which combine as a proof-of-concept for the modelling approach developed below. First, we aim to survey the trade-off space arising from upgrading a pre-existing farmer-facing horticultural aggregation scheme to improve the availability of F&V in small retail-oriented markets in Bihar, India. Second, we aim to identify the multidimensional scenarios, causal pathways and driver interactions underpinning the trade-offs. Third, we aim to isolate the driver pathways leading to ‘win-win-win’ futures: simultaneously improving the availability of F&V in small markets, the financial profits of producers and the financial sustainability of the aggregation scheme.

Our research also makes three contributions to the existing literature. First, models of agricultural trade-offs have traditionally focused on either the optimisation of *farm-level* outcomes like labour costs and soil nutrient losses (Dogliotti et al., 2005; Groot et al., 2012), or, *landscape-scale* trade-offs, such as between land use and sustainability objectives (Coleman et al., 2017; Parish et al., 2012). Moreover, efforts to incorporate markets have traditionally required the integration of multiple models with different assumptions, languages and spatiotemporal characteristics (Kanter et al., 2018). We complement existing approaches by modelling holistic value chain processes and trade-offs within a single model.

Second, this paper furthers efforts to capture explicit food system trade-offs using system dynamics modelling. Built from structures of stocks, flows and feedbacks, system dynamics models (SDM) are suited to exploring the accumulations and movements of goods, finance, and information (Nicholson et al., 2020; Stave and Kopainsky, 2015; Sterman, 2000). Various studies have developed qualitative tools linking production and food security (Langellier et al., 2019; Rammelt and Leung, 2017; Walters et al., 2016), but stop short of quantitative analysis. Although Dizyee et al. (2017) analysed the economic impacts of different interventions amongst different livestock value chain actors, quantitative SDMs have traditionally only addressed the interactions between value chain upgrades and producer-facing outcomes (Lie et al., 2018; von loeper et al., 2016). This paper seeks to expand and deepen such trade-off analysis by modelling the flows, feedbacks and trade-offs between producers and consumers in a transferable and generalisable way.

Third, we further attempts to identify ‘desirable futures’ (Bai et al., 2016) from ‘possibility spaces’ of interacting scenarios and unintended consequences (Srinivasan et al., 2017). Stemming from the global need to achieve progress across multiple and often competing social-ecological dimensions (UNDP, 2012), the concept of ‘win-win-wins’ has provided a lens to view the complex trade-offs involved in climate smart agriculture (Karlsson et al., 2018), trade liberalisation (Campling and Havice, 2013), and sustainable resource use (Cooper and Dearing, 2019). Developing these concepts around agricultural systems will help to identify upgrades that generate both producer-facing and consumer-facing benefits whilst avoiding undesirable trade-offs.

We develop our approach around the ‘Loop’ aggregation scheme (Digital Green, 2017), which aggregates F&V at the village level, before transporting supplies to markets across Bihar, India. An aggregator is assigned to each cluster of two to three villages, with farmers that have joined Loop (‘Loop farmers’) opting whether to send their produce to market through Loop on any given day. Aggregators record market transaction details (e.g. crop type, quantities, prices) on a smartphone to provide farmers with digital receipts of past aggregations.

**Fig. 1:**
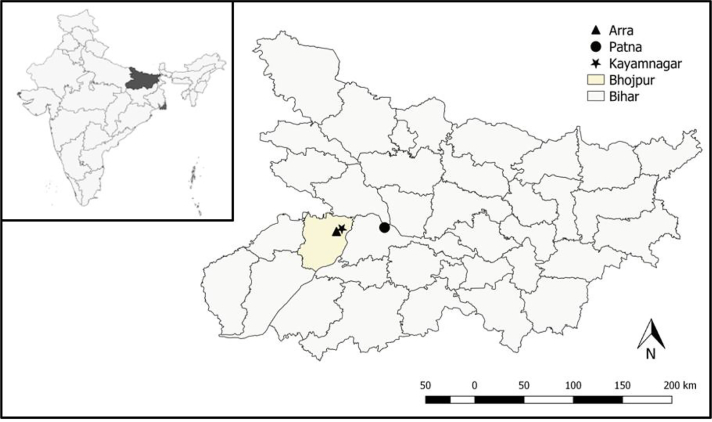
The horticultural markets of Arra and Kayamnagar in Koilwar block, Bhojpur district (yellow), Bihar state (dark grey inset), India.

Over 28,000 farmers have supplied Loop since 2016, aggregating over 95,000 t of F&V to 150 markets. Consistent with the original aims of Loop, farmers report transport costs being cut in half and time savings of between 4 and 8 h each week by removing the need for them to visit the market (Digital Green, 2017). However, the destinations of Loop supplies are primarily urban, owing to the bulky quantities of F&V aggregated and the opening-up of higher demand markets that were previously inaccessible to farmers without transport. For instance, the urban markets of Arra and Kayamnagar (Fig. 1) received 95% of Loop supplies in Bhojpur district since January 2016, with the remaining 5% spread between five local markets.

Within this context, Loop provides a case study to develop a novel modelling framework for the identification of trade-offs and associated causal dynamics. Therefore, specific management recommendations for Loop are secondary to the broader implications for how we view value chain trade-offs and guard against their occurrence.

## 2 Materials and methods

Based upon qualitative and quantitative data (Section 2.1), we develop an SDM of Loop within its wider horticultural system. The model (Section 2.2) is run under a spectrum of Monte Carlo scenarios (Section 3) towards a number of pre-defined outcome spaces (Section 4), involving the availability of F&V in a local small market environment (‘Market B’, rather than the large urban wholesale ‘Market A’), Loop farmer profits, and the financial sustainability of the aggregation scheme itself. We then trace outcomes back to their scenario trajectories and internal driver dynamics to understand the pathways towards ‘win-win-win’ futures, different system trade-offs and unintended consequences.

The model was built in STELLA Architect v.1.6.1 (ISEE Systems, 2018) on a 64-bit Windows 10 device, with each simulation taking approximately five seconds to run and store outputs as CSV files. Whilst data was collected from Muzaffarpur and Bhojpur districts, the value chains were not found to be significantly different. Therefore, rather than developing separate models, the parameters of the model are based on Koilwar block in Bhojpur district (Fig. 1), where Loop has been active since mid-2017.

The model runs at a half-day resolution from October 1st, 2017 until September 30th, 2021 to simultaneously capture various short-term dynamics of fresh produce (e.g. availabilities and prices), whilst exploring how outcomes may evolve over multiple seasons. Moreover, consistent with our aim to model total F&V availability and affordability, all 47 Loop F&V types detailed in the ‘Loop dashboard’ (Section 2.1.1) are aggregated into one F&V variable by summing the daily supplies from Koilwar block. Therefore, to explore system-level dynamics, F&V is modelled as a homogenous entity, meaning outcomes explicit to the different physical and nutritional qualities of different F&V types are not investigated here (e.g. human health benefits from consuming different F&V types).

### 2.1 Datasets informing model design

SDMs may be informed by a spectrum of data: from physical laws such as mass balances to qualitative stakeholder perceptions (Ford, 2010). Prior to data collection, ethical clearance was obtained from the ethics boards of the university leading the research (SOAS Research Ethics Panel: 103_BS_VREP_DEC_18) and the external survey agency (Centre for Media Studies Delhi Institutional Review Board: IRB00006230). Moreover, written consent was sought before stakeholder engagement, with additional consent sought before audio recordings were made.

#### 2.1.1 Value chain analysis

A value chain analysis involving 49 semi-structured interviews was conducted to establish the structures of actors, decision-making processes and governance underlying the horticultural system (March–September 2018). Interviews with farmers, aggregators, and market-based commission agents focused on farming and marketing activities, whilst interviews with wholesalers, retailers, and consumers concentrated on the flows of F&V downstream of markets, namely the decision-making processes around availability, quality and prices.

These qualitative insights were supplemented by the ‘Loop dashboard’ (www.loopapp.org/loop/analytics; full data available on request from Digital Green): a near-real time record collected by the Loop smartphone application, detailing the types of F&V sold through Loop, their quantities and prices, plus various meta-datasets detailing village and market locations, and the costs involved in aggregation. Here, ~46,300 transactions originating from Koilwar block informed model parameterisation (302 half-days between October 2017–February 2018) and evaluation (366 half-days between March–August 2018).

#### 2.1.2 Household and value chain surveys

We conducted 360 farmer-household surveys in February 2019 to increase the volume of quantitative data. The surveys were administered in Bhojpur and Muzaffarpur districts – the only two districts where Loop was active at the time of study. To understand how outcomes may be affected by market association, we considered whether the market had received above or below the mean Loop quantity per market. One market was randomly sampled from each size category per district, followed by a randomly sampled village for each market. The four Loop villages were then paired with villages which met the following conditions: (a) farmers from the ‘non-Loop’ village had not participated in Loop in the last year before the survey (March 2018–February 2019), and (b) the village exhibited similar market access to the respective Loop village (e.g. transport infrastructure). In total, we surveyed 120 Loop and 240 non-Loop farm-households – with half of the non-Loop households located in Loop villages and the other half located in non-Loop villages. The surveys covered land ownership, F&V production and marketing, and Loop participation and adoption. We also conducted 28 quantitative surveys with traders in Koilwar block (Bhojpur) and Minapur block (Muzaffarpur) to understand the capacities, commissions and the costs associated with horticultural trade.

#### 2.1.3 Group model building

There is growing recognition around the importance of contextual qualitative information in systems modelling (Meadows, 2009; Rich et al., 2018). ‘Spatial group model building’ (SGMB) combines the concepts of systems dynamics and Geographical Information Systems (GIS) to develop causal-loop diagrams that describe the key problematic behaviours and feedbacks underlying the system (Mumba et al., 2017). Building upon traditional group modelling approaches (Vennix et al., 1997), the LayerStack tool is an offline GIS framework made up of acetate sheets that overlay maps of the system to facilitate discussions about how value chain dynamics vary over space (Rich et al., 2018). To date, SGMB has contributed to the modelling of East Coast Fever in Zambia (Mumba et al., 2017), the planning of urban agriculture in New Zealand (Rich et al., 2018), and the implementation of a livelihoods development programme in Myanmar (Berends et al., 2020).

We conducted ten SGMB sessions between January and April 2019, split evenly between the districts of Bhojpur and Muzaffarpur. The aims, activities, and timings of each session were planned in advance, with sessions lasting three hours and consisting of up to five Loop farmers, two aggregators, one commission agent, two ‘out-of-state’ distance traders, one local wholesaler, and one local retailer. The first two sessions introduced systems thinking and established the feedbacks determining market capacities, supplies, and demands. The third session focused on seasonal and intra-daily price dynamics, while the remaining sessions concentrated on Loop adoption, day-to-day participation, and market choice.

### 2.2 Model description

**Fig. 2:**
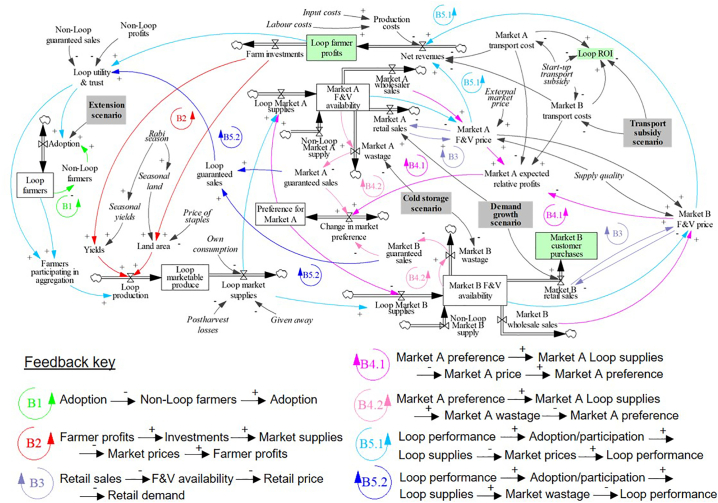
Stock and flow diagram summarising the key variables and feedbacks driving F&V supplies towards the large urban ‘Market A' and the smaller retail-oriented “Market B'. Variables in italics represent external drivers; grey boxes represent the four future scenarios (Section 3); green boxes represent the three outcome dimensions (Section 4). Polarities: ‘+’ positive driver-outcome relationship; ‘−’ inverse driver-outcome relationship”; ‘B’ – balancing feedback. Note: this schematic does not disaggregate intra-market trader dynamics (Section 2.2.3).

Arising from the value chain and SGMB discussions, we developed a systems model to capture the key feedbacks driving F&V supplies downstream to markets in Bhojpur, as well as financial returns and market information back upstream (Fig. 2). All datasets informing the model are listed in Supplementary Material A, and model equations and parameters are listed in Supplementary Material F.

#### 2.2.1 Loop adoption

The total farmer population in Koilwar block (12,087, Census of India, 2011) is split between Loop and non-Loop farmers (Fig. 2), with the initial Loop population equalling 90. Loop adoption is based on the Bass (1969) diffusion model, where adoption is driven by community extension efforts and the benefits of the scheme spreading via word-of-mouth. This feedback structure leads to an increase in Loop farmers over time as the benefits of aggregation spread across the farmer population (B1, Fig. 2).

Based on SGMB discussions, we first extend the original model by relating the adoption rate to the *trust* Loop farmers have in aggregation to continue providing financial benefits and guaranteed sales (i.e. sell all of the produce supplied). Trust increases if profits and/or guaranteed sales increase over the course of a month, leading to more adoptions and less dis-adoptions. Second, the adoption rate is also influenced by the *utility* of aggregation (Grabowski et al., 2019), in terms of the profitability and guarantee of sales from Loop relative to non-Loop.

#### 2.2.2 F&V supplies

Marketable F&V production in the model is a function of seasonal yields, land under F&V cultivation, and the number of Loop and non-Loop farmers marketing produce on any given day (Fig. 2). The latter driver assumes that both populations market F&V once every five days in the Rabi season (October–February), once every seven days in the Zaid season (March–May) and once every eight days in the Kharif season (June–September). However, Loop aggregation only happens in the morning, whilst non-Loop farmers may supply markets in the morning and afternoon.

Seasonal yields are a function of (i) a baseline timeseries of the yields of the three most aggregated crops per season in the Loop dashboard (NHB, 2015; NIFTEM, 2013) (Rabi 2017–2018: brinjal, cauliflower and cabbage accounted for 70% of aggregation; Zaid 2018: cauliflower, tomato and bottle gourd accounted for 50%; Kharif 2018: bottle gourd, bitter gourd and sponge gourd accounted for 73%), and (ii) financial investments made by farmers over time (Section 2.2.5). Average Loop and non-Loop cultivatable land areas were derived from the household surveys, with the proportion of land area under F&V cultivation cycling through a peak of ~80% in the Rabi to a minimum of ~50% in the Kharif (Fig. 2). The proportion of land under F&V cultivation is then further influenced by the price of F&V relative to the price of staples (Fig. 2). Due to a lack of regional, monthly resolution data, we parameterise the price of staples as a timeseries of rice, wheat, and maize prices across India between 2017 and 2018 (USDA, 2019). In turn, F&V quality is expressed as the percentage of production that is high-grade, ranging randomly between 60 and 100%.

Three non-marketing outflows subtract from the production stocks: (i) own F&V consumed by the farm households, (ii) F&V given away, and (iii) F&V lost prior to the farmgate (Fig. 2). Baseline rates of own consumption are modified by the proportion of female farmers in each population, with the latest NSSO data for Bihar (2011−2012) finding that female headed households consume ~10% more F&V per day than male headed households (Table S1). In turn, the model calculates the proportion of Loop farmers opting to market their produce via aggregation on any given morning – depending on Loop profits and guaranteed sales relative to non-Loop over the last week (B5.1 and B5.2, Fig. 2). Loop production that is not aggregated is added to the non-Loop marketable stock.

From the farmgate, Loop farmers must choose between supplying Market A or Market B, which differ in terms of the (i) number of different trader types present, and (ii) daily capacities (Section 2.2.3). The proportion of Loop supplies sent to Market A is equal to the market preference stock, which varies between 0 and 100%, driven by the expected marketing profits and guaranteed sales of each market over the last week. Marketing profits equal the market revenues minus the commission and transport costs. As per dashboard data, aggregating farmers incur transport costs of 0.50–0.85 Rs/kg to Market A, and 0.80–1.10 Rs/kg to Market B (both subsidised by up to 50% by Digital Green over the first year to incentivise adoption). In contrast, the costs of self-supplying either market vary randomly between 1.00 and 1.50 Rs/kg. Consistent with Swinnen and Maertens (2007) and Minten et al. (2009), the change in market preference is weighted towards securing guaranteed sales, with farmers in the SGMB sessions emphasising the importance of marketing efficiency over receiving the highest price possible. Balancing feedbacks (B4.1 and B4.2, Fig. 2) emerge as without equivalent increases in market demand, increasing supplies to either market would lead to less favourable prices and higher chances of unsold produce. In turn, non-Loop market proportions are set as constants (Supplementary Material F), plus or minus a random proportion up to 10% of the constant value per timestep, designed to provide background noise without generating large swings of non-Loop produce.

Loop and non-Loop suppliers must then choose the type of trader to supply at the market. The three-way choice in Market A is between: (i) ‘distance traders’, only operating in the morning with export capacities of 2000 kg/trader/half-day, (ii) local wholesalers operating all day, with capacities of 600 kg/trader/half-day, and (iii) local retailers operating all day, with capacities set by customer demands (Section 2.2.4). The choice in Market B is between wholesalers and retailers in the absence of distance traders. As with market choice, the percentage of Loop and non-Loop supplies sent to each trader is driven by the expected price and guaranteed sales of each trader.

#### 2.2.3 Market processes

The prices paid by market traders for produce are driven by three factors: (a) the volume of F&V available to each trader, relative to their downstream demand (assumed equal to their capacities) (Sterman, 2000); (b) trader expectations of prices when selling the F&V downstream; and (c) produce quality.

The expected price of distance traders is a timeseries generated by the weighted average weekly price of brinjal, cauliflower, cabbage and bitter gourd arrivals at wholesale markets in Patna between October 2017 and September 2018 (NHB, 2018) – repeated over the duration of the simulation (Supplementary Material F). During SGMB, distance traders were identified as the major source of price information; therefore, all other traders base their expected price on the price of distance traders (Fig. 2). The relative proportions of high-grade (receiving full price) and low-grade (receiving half price) supplies then produce a weighted average price per trader.

Markets A and B are parameterised with approximate capacities of 100,000 kg/day and 20,000 kg/day during the peak Rabi season, respectively, and change as a function of the numbers of traders in the market. The baseline numbers of each trader type are parameterised with SGMB timeseries, and market entries are proportional to the profits of each trader type over the last month (Sterman, 2000). Trader revenues are generated by multiplying their purchases (minus wastages) by their selling price, which is the quality-adjusted price offered to farmers plus their profit margin. Trader costs are the sum of F&V purchase and transport costs, market commissions, cold storage rent, and other miscellaneous costs (e.g. tolls and bribes) (Supplementary Material F).

#### 2.2.4 Retail demand

The F&V purchases of retail customers are driven by the selling prices of retailers in Markets A and B, and the respective expenditure elasticities for urban and rural markets in Bihar (Kumari and Singh, 2016). These dynamics form balancing feedbacks (B3, Fig. 2), whereby increased availability leads to lower prices and higher retail demands, which then feedback to generate lower availabilities and higher prices. The purchase demands per customer are multiplied by the number of customers per retailer to generate the demand per retailer and feedbacks with price (Supplementary Material F). Demand changes are smoothed under the assumption that consumers adjust their demand once per week (i.e. once every two market visits). To assess implications for model behaviour, the elasticities and smoothing coefficients undergo sensitivity analysis in Section 2.3.

#### 2.2.5 Farmer outcomes

Weighted average revenues for Loop and non-Loop farmers factor in the number of farmers that supplied each trader type on any given half-day. Likewise, the average guaranteed sales per market and per farmer population are calculated from the total supplies to each market and total sales. From here, average guaranteed sales drive Loop trust and utility, which then feed into Loop adoption and daily participation (B5.1 and B5.2, Fig. 2).

The daily costs of production, labour, transport and commission are subtracted from the average revenues to generate daily average profits per farmer population (Supplementary Material F). Each on-farm cost variable randomly samples a value from a distribution parameterised by household survey data, which are then summed and converted into half-day costs per farmer population. Daily profits then accumulate as a stock over time, from which farmers can invest in F&V production at the start of each season (B2, Fig. 2). In the absence of reliable quantitative data, we conservatively assume farmers only invest in land if their cumulative profits multiplied by their land investment rate (20%) is greater than the cost of one-tenth of one katha (~40 m^2^, priced ~76,000 Rs/katha). If met, then farmers purchase an area equivalent to 20% of their cumulative profits. Likewise, farmers invest 10% of their cumulative profits on enhanced yields at the start of each season, where every Rs 20,000 invested produces a 1% increase in yield. These investments feedback to increase production by a maximum of 1.5%/year – in line with GoI (2018) data for Bihar between 2013 and 2018.

### 2.3 Model evaluation

A model built from the best available knowledge is not automatically a useful learning tool (Sterman, 2000), owing to the uncertainties inherent to data and mental abstractions of reality. Here, we detail the two stage Monte Carlo sensitivity analysis performed to understand how model behaviours react when (i) subject to individual (‘one-at-a-time’) parameter uncertainties, and (ii) subject to the interacting uncertainties of the most sensitive variables (‘multivariable analysis’), identified during the first stage (Cooper and Dearing, 2019; Spear and Hornberger, 1980). See Supplementary Materials A-D for tests of data reliability, behaviour reproduction, integration error and model performance under extreme conditions.

The first stage sensitivity analysis was conducted on the 47 variables found to be qualitatively unreliable through the parameter assessment methodology of Chapman and Darby (2016) (Supplementary Material A). Error magnitudes were relative rather than absolute as not all variables had historical data distributions. Thus, each parameter was perturbed by up to ±25% of its central parameterised value (*V*_*0*_) over 500 simulations:

*V*_*i*, *j*_ = *V*_0, *i*, *j*_ × *ε*_*i*, *j*_ 0.75 ≤ *ε* ≤ 1.25 (1)

where *i* is the given variable (n_*i*_ = 47), *j* is the given sensitivity simulation (n_*j*_ = 500), and *ɛ* is the relative error magnitude.

Constraint corridors were generated from the 95% confidence intervals of locally weighted least squares regression (LOESS) models for daily Loop sales in Market A (e.g. Fig. S7) and total Loop farmers (e.g. Fig. S8). Covering March 1st–August 25th 2018, the constraint corridors ascertain whether historical behaviours are produced to within acceptable confidence intervals. Of the 47 variables tested, 33 generated timeseries with at least 95% of all output values falling within their constraint corridors (Table S4), meaning quantitative uncertainties in the majority of qualitatively unreliable parameters do not have significant implications for model behaviour.

**Fig. 3:**
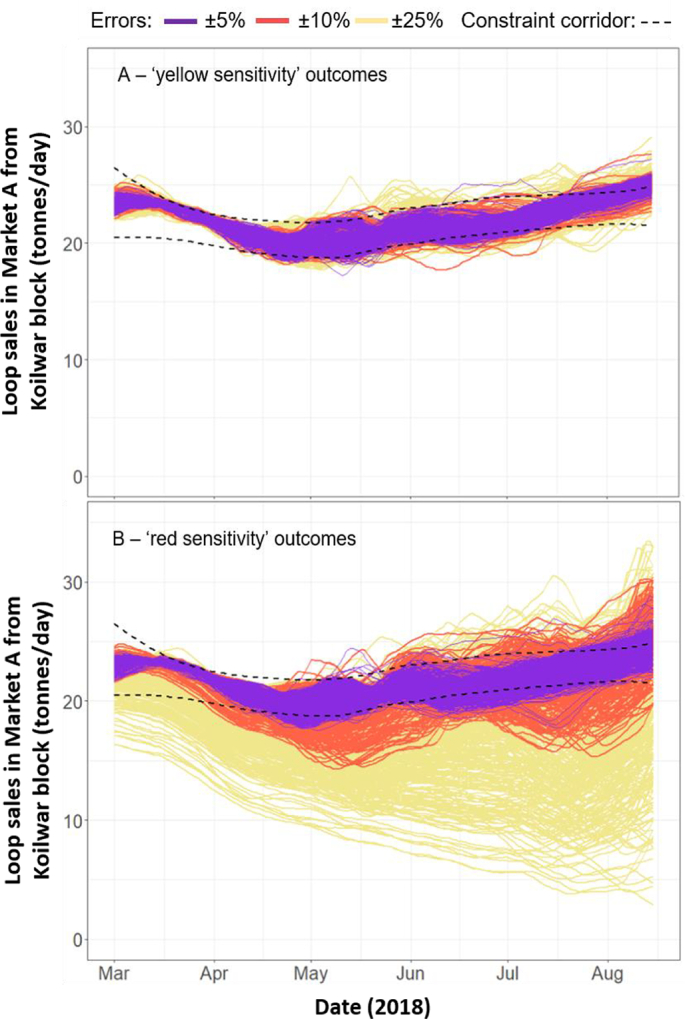
Outputs from the multi-variable sensitivity analysis, where the variables found to be sensitive during the one-at-a-time analysis were simultaneously varied across three error ranges. The six ‘yellow sensitivity’ variables each had at least one error subset with less than 95% of supply quantities within the first stage constraint corridor (Table S4); the eight ‘red sensitivity’ variables each had at least one error subset with less than 90% of supply quantities within the first stage constraint corridor (Table S4).

For the second stage, the 14 remaining variables were then split into (Table S4): (i) six moderately-sensitive ‘yellow sensitivity’ variables, and (ii) eight most-sensitive ‘red sensitivity’ variables (see Fig. 3). The ‘yellow sensitivity’ variables were sequentially perturbed by ± 5%, ± 10% and ± 25% of their parameterised values, followed by the ‘red sensitivity’ variables under the same error ranges. As expected, for both subsets, the similarity between model outputs and reality weakens as errors widen (Fig. 3A-B). For the ‘yellow sensitivity’ group (Fig. 3A), 90.4% of all values in the ±5% error range fall inside the constraint corridor, before falling to 88.1% for the ± 25% error range. For the ‘red sensitivities’ (Fig. 3B), 88.3% and 63.6% of all values in the ± 5% and ± 25% error ranges fall inside the constraint corridor, respectively.

The Kolmogorov-Smirnov test (Massey, 1951) identifies influential parameters with significant differences between the behaviour-giving and non-giving error subsets (*ε*_*i*, *j*_), where behaviour-giving subsets have at least 95% of outcomes falling within the constraint corridor (Spear and Hornberger, 1980). The two most influential determinants of model behaviour control the quantities of non-Loop F&V to Market A (Supplementary Material E3). Consistent with reality, non-Loop supplies constitute >90% of all F&V supplies to large urban markets in Bihar and are thus major drivers of market dynamics.

Overall, the model tracks the historical trends of three key outcomes (Supplementary Material B) and remains robust under extreme conditions (Supplementary Material C). Positive for model reliability, sensitivity analysis finds that the parametric uncertainties underlying qualitatively unreliable variables match our understanding of the system, whilst robustly reproducing reality across wide relative error ranges.

## 3 Future scenarios

Rather than narrowly forecasting multidimensional value chain trade-offs under the most likely system trajectory, we investigate how retail-based F&V availability, Loop farmer profits, and the sustainability of the Loop aggregation scheme may co-evolve across four simultaneously varying exploratory scenarios.

Running the model unchanged from the validation period until September 2021 represents the baseline *do-nothing* scenario. As in reality, Loop does not currently recruit farmers through extension or influence market or trader choices, meaning aggregation membership and participation remain driven by profitability and guaranteed sales.

In turn, the exploratory future scenarios have two dimensions (Table 1): (i) the type of system change, and (ii) the magnitude of change from the baseline. Whilst the number of potential changes is theoretically limitless (i.e. an infinite number of new variables), we simulate the simultaneous effects of two internal changes to aggregation and two changes to the wider value chain, which were all hypothesised to increase F&V availability in local markets during SGMB (Table 1).

**Table 1:** The minimum and maximum limits of the four scenario types generating the Monte Carlo simulations.

Scenario type	Variable name	Units	Scenario magnitude range		Explanatory notes
			Min.	Max.	
1. Scaling-up Loop	Loop extension rate	Farmers per 1000 non-Loop farmers per half-day	0	0.274	The ‘innovation coefficient’ of the Bass (1969) model; sampled between no active recruitment and the historical maximum for Koilwar block
2. Loop transport subsidy	Market B subsidy	%	0	100	Covers the plausible range between no subsidies and the complete subsidy of supplies to Market B.
3. Cold storage (multi-stage)	Cold storage active	Unitless	0	1	Cold storage is only present if ‘cold storage active’ is greater than or equal to 0.5.
	Cold storage investment level	Unitless	0	3	Cold storage investment of ‘1’ provides 25,000 kg/half-day in Market A and 5000 kg/half-day in Market B; an investment of ‘3’ provides Market A with 75,000 kg/half-day and Market B with 15,000 kg/half-day
4. F&V demand annual growth rate	External retail reference demand growth rate	%/year	0	10	The externally driven demand growth is varied between ‘no growth’ and an extreme increase for both markets.

Scaling-up Loop membership may increase the aggregated volumes supplied towards Market B, whilst subsidising transport costs may encourage supplies to Market B by offsetting possible profit losses relative to supplying Market A. In turn, Bihar has the third widest horticultural cold storage deficit of all Indian states (Vanitha et al., 2013), with evidence from the state's potato chain suggesting that cold storage can dampen price fluctuations and stabilise demands (Minten et al., 2011; Rich and Dizyee, 2016). Lastly, representing possible community-level and/or government-led behaviour changes, the reference retail demands for F&V (for a given price) are increased to explore how the other scenarios interact with a growing awareness around the importance of F&V consumption.

To generate multidimensional driver pathways, we simultaneously vary the magnitudes of the four scenarios across 5000 Monte Carlo simulations. Scenario magnitudes are randomly sampled from uniform distributions between plausible minimum and maximum trajectories (Table 1). The cold storage capacity per simulation equals ‘Cold storage active’ (either 0 or 1) multiplied by the ‘Cold storage investment level’ (Table 1). A trader's share of the storage capacity is assumed proportional to their share of all F&V supplies to that market, and rent is paid by traders at 0.15 Rs/kg-stored/half-day (NHM, 2020). Lastly, cold storage is assumed to increase F&V shelf-life from two to 21 days. Each of the future scenarios start from October 1st 2018 (the 669th half-day) and run until September 30th 2021 (the 2922nd half-day).

## 4 Trade-off space definition

Each of the 5000 simulations are placed within a pre-defined three-dimensional trade-off space, where desirable ‘win-win-win’ futures simultaneously achieve positive outcomes relative to the baselines of three traditionally competing outcomes. This classification contributes to the search for value chain upgrades that minimise trade-offs by achieving mutual benefits at both ends of value chains (Kadiyala et al., 2012; Thow et al., 2018). The trade-off space is constructed from the following three outcomes:

— *Cumulative F&V purchases per retail customer in Market B*: F&V purchases by consumers in the smaller, traditionally access-limited market are a function of availability and affordability.
— *Cumulative horticultural profits per Loop farmer*: evaluates the impacts of F&V access-sensitive upgrades on the financial takeaways of farmers.
— *Mean average Loop return on investments (ROI)*: the ratio of transport costs collected from Loop farmers (post-subsidy), relative to the amount paid by the scheme to compensate aggregators (0.1 Rs/kg sold). An increasing ratio means ROI is improving.

The trade-offs operate between ‘win-win-win’ and ‘lose-lose-lose’ spaces. The remaining spaces between the extremes are aggregated to identify the main trade-offs: (i) win-win ‘access and profit wins’, (ii) ‘access wins’ but farmer profit losses; (iii) farmer ‘profit wins’ but access losses, and (iv) ‘others’ – where ROI wins, but F&V availability and profits lose.

## 5 Results

### 5.1 Operationalising the trade-off space

We first visualise the array of trade-offs emerging from the 5000 simulations, before tracking their causal scenarios and driver pathways. We define a ‘core’ subset of outcomes within each trade-off category to help distinguish the pathways, populated by simulations which have three-dimensional cartesian distances (i.e. purchases, profits, and ROI) from the baseline greater than the threshold distance for each category (equal to the mean three-dimensional cartesian distances plus one standard deviation).

**Fig. 4:**
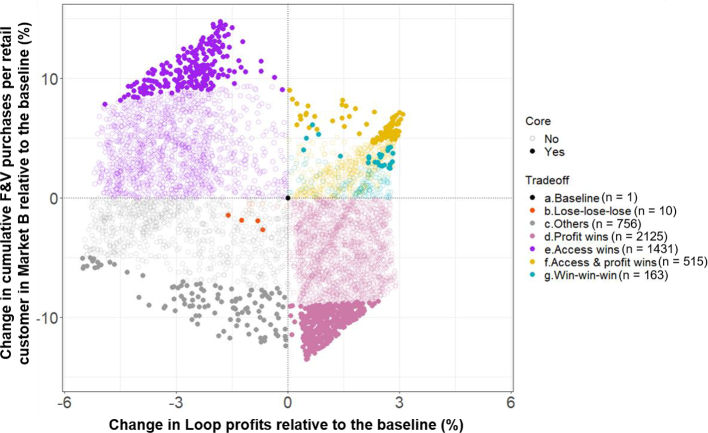
Trade-offs resulting from the 5000 plausible futures. Here the trade-off space is plotted in two dimensions for readability, with the three-dimensional trade-off categories depicted in colour. Runs depicted in solid colour fall within the ‘core’ of each trade-off category.

Cumulative F&V purchases by consumers in Market B increase in 42% of all simulations (Fig. 4); however, 68% of these are associated with simultaneous declines in Loop farmer profits relative to the baseline. Second, Loop farmer profits increase in 56% of all simulations, but 75% of these are associated with weakened cumulative F&V purchases relative to the baseline. Worryingly, only 163 (3.3%) simulations co-produce positive outcomes for Loop farmer profits, F&V purchases in Market B and Loop ROI. Positively however, only 10 simulations are ‘lose-lose-lose’, meaning that the upgrade scenarios benefit at least one outcome in 99.8% of futures.

The overall trade-off space indicates that attempts to align aggregation schemes with small market F&V delivery risks degrading farmer profits and/or the sustainability of the aggregation scheme. Beneath the trade-off category overlays, the division of the space into two distinct zones suggests the potential presence of nonlinearities, where particular scenario ranges or interactions split outcomes towards either ‘access wins’ or ‘profit wins’. To this end, the next section investigates the causes of the above trade-offs and the pathways to avoid them.

### 5.2 Multidimensional driver pathways

Whilst the scenarios could be plotted as timeseries, their constant values per simulation would appear as horizontal lines. Therefore, conditional probability plots illustrate how the chances of producing the different trade-offs vary across the four scenario ranges. Moreover, to illuminate the internal pathways towards the trade-off categories, we visualise the timeseries of five key drivers of small market F&V availability, Loop farmer profits and participation.

**Fig. 5:**
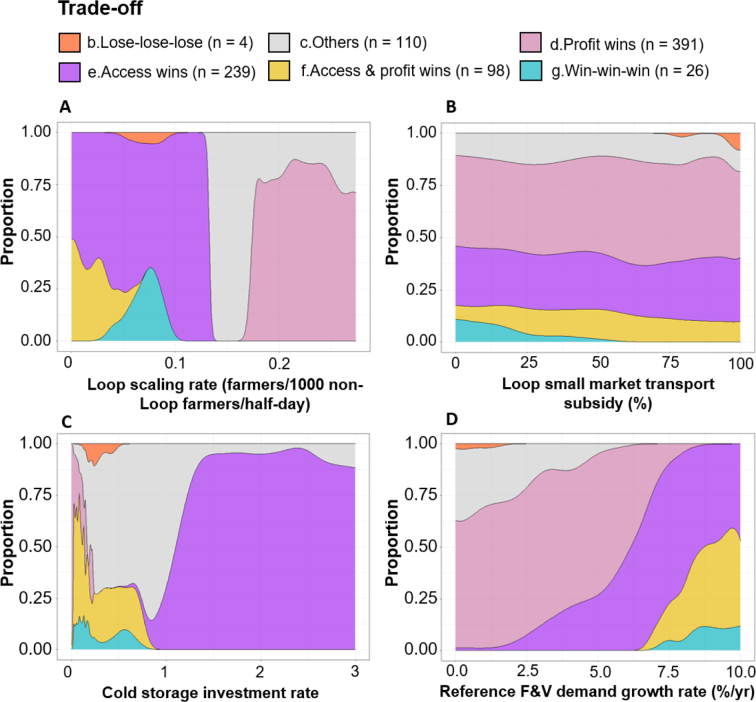
Conditional probability plots illustrating how the proportion of different core trade-offs vary over the scenario ranges. ‘Win-win-win’ futures may only be achieved if the individual ‘win-win-win’ scenario trajectories are simultaneously achieved.

**Fig. 6:**
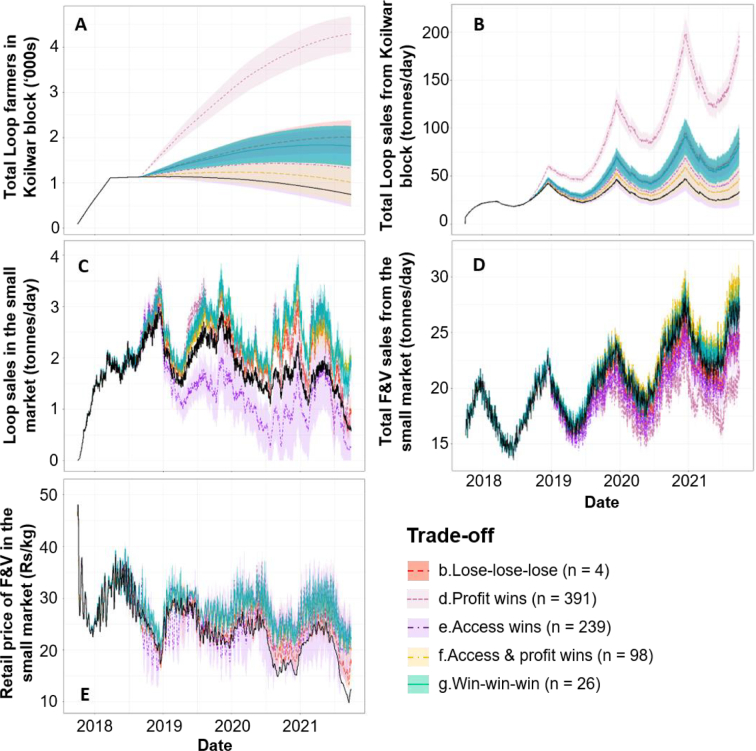
Timeseries (solid) and the 95% confidence intervals (shading) of five internal drivers of F&V availability in Market B, as coloured by their respective trade-off category. Black lines represent the baseline simulation. ‘Win-win-wins’ have been plotted last for visibility.

The 26 core ‘win-win-win’ futures correspond to a distinct but limited scenario space, formed of relatively low rates of Loop scaling, transport subsidy and cold storage investment, but rates of retail F&V demand growth above 7%/year (Fig. 5). ‘Win-win-wins’ therefore represent a delicate balance, with the external growth in demand boosting prices in Market B by an average of 6.4 Rs/kg above the baseline scenario (Fig. 6E), which offsets potentially weaker prices and profits from heightened Loop supplies to Market B (Fig. 6D). In contrast, ‘lose-lose-lose’ scenarios entail the opposite combination of relatively high rates of transport subsidy (i.e. unsustainable for the intervention), low rates of demand growth (i.e. Market B remains relatively unprofitable), and low rates of cold storage (i.e. no extension of F&V storage-life).

In turn, cold storage investment rates of at least 1.5 (~75% of daily market capacities) sharply separate ‘access wins’ from ‘win-win-wins’ and ‘profit wins’ (Fig. 5C), with weakened farmer profits emerging as an unintended consequence of dampened trader demands from the storage and accumulation of F&V supplies (Fig. 6D-E). As an illustration, while ‘access win’ Loop sales in Market B occasionally fall to less than 1000 kg/day (Fig. 6C), F&V prices still average 1.1 Rs/kg lower than the baseline scenario (Fig. 6E). However, such profit losses can be partially offset by the steepest rates of retail demand growth, which shift outcomes towards the right-hand side of the ‘access wins’ space (Fig. S6).

In contrast, moving from ‘win-win-wins’ to ‘profit wins’ requires relatively steep rates of Loop extension (Figs. 5a and 6a). These trajectories trigger a critical mass of aggregating farmers to switch from supplying Market B to the relatively profitable Market A, producing total sales of F&V in Market B that average 4000 kg/day less than the baseline (Fig. 6D). Here, reductions in Market B F&V purchases under the highest rates of Loop extension can be partially offset by the highest rates of external demand growth (Fig. S6), with local consumers becoming more resilient to seasonally lower F&V availabilities (Fig. 6D) and higher prices (Fig. 6E).

Ultimately, different trade-off categories may be achieved by following particular external trajectories of aggregation extension, cold storage capacity and retail demand growth. In turn, the exact position of each outcome within each trade-off category is a function of two interactions stretching across the length of the value chain, with Loop extension working to undermine downstream Market B F&V availability, and the retail demand growth positively influencing the financial takeaways of Loop farmers upstream.

### 5.3 Refining pathways to ‘win-win-win’ futures

In an attempt to reinforce the pathways towards ‘win-win-win’ futures from the broader horizon scan, we run an additional 500 simulations, randomly sampling between the ranges of the initial core ‘win-win-win’ scenarios (Fig. 5):

• Loop scaling rate (farmers/1000 non-Loop farmers/half-day): 0.035–0.086;
• Loop small market transport subsidy (%): 0–50;
• Cold storage investment (unitless): 0–0.62;
• F&V reference demand growth (%/yr): 7.5–10.0.

**Fig. 7:**
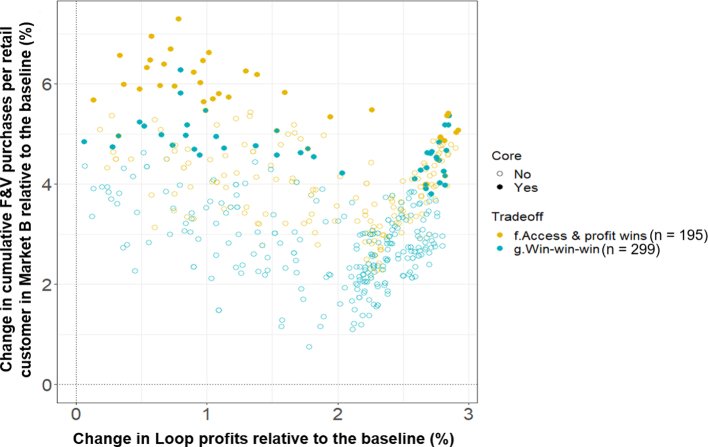
Trade-off space resulting from the second phase of 500 simulations. Six simulations (1.2%) which did not achieve either ‘win-win-win’ or ‘access & profit win’ futures have been removed here for visual clarity, enabling only the top-right quadrant of the full trade-off space to be shown.

Here, 60% of all runs achieve ‘win-win-win’ futures relative to the baseline, with 39% simultaneously achieving ‘win-win’ futures for cumulative F&V purchases and farmer profits (Fig. 7). A negative correlation exists within the ‘win-win-win’ space between Loop farmer profits and F&V purchases (R^2^ = 0.13, *p* < 0.05, df = 295), representing a trade-off even within the most desirable space. However, the relationship is positively correlated beyond cumulative profit increases of 2% (R^2^ = 0.40, p < 0.05, df = 180), driven by extreme reference F&V demand growth rates (>9%/year) inflating trader demands to the extent that they exceed the potential farmer profits generated from supplying Market A.

**Fig. 8:**
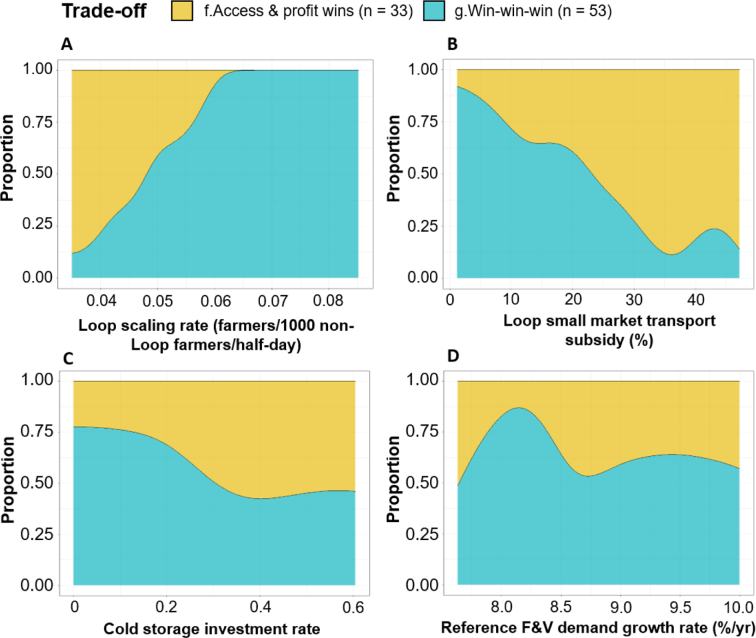
Conditional probability plots illustrating how the proportion of core ‘win-win-win’ and ‘access and profits wins’ vary over the second phase scenario ranges.

**Fig. 9:**
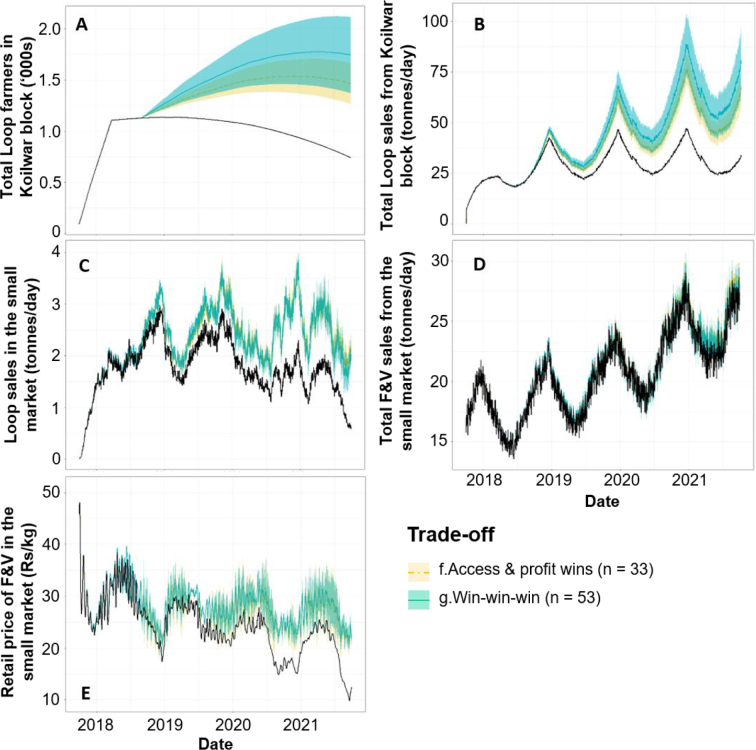
Second phase timeseries (solid lines) and 95% confidence intervals (shading) of five internal drivers of F&V availability in Market B. Black lines equal the baseline scenario.

In contrast to the shape of the overall trade-off space (Fig. 5), the internal Loop scenarios are key to determining whether ‘win-win-win’ or ‘access and profit wins’ are produced (Fig. 8A-B). Whilst there are no discernible differences between the two spaces in terms of the availability (Fig. 9D) and price (Fig. 9E) of F&V in Market B, ‘win-win-wins’ are dependent upon a sufficient number of Loop farmers (Fig. 9A) pooling adequate volumes of F&V (Fig. 9C) and covering transport costs with minimal subsidisation (Fig. 8B). In contrast, lower rates of Loop extension (Fig. 8A) and higher rates of small market transportation subsidy (Fig. 8B) produce lower ROI values than the baseline scenario.

Importantly, relative to the wider spectrum of futures (Fig. 5), both trade-off categories in this second phase are supported by comparatively low rates of extension and cold storage capacity. Therefore, the multidimensional pathways developed here illustrate the complex balances needed to achieve ‘win-win-wins’ across often competing dimensions. For instance, whilst a positive relationship exists between aggregation extension and the likelihood of ‘win-win-wins’ in the narrower second phase (Fig. 8), the first phase shows that simply doubling the scaling rate from 0.1 to 0.2 (farmers/1000 non-Loop farmers/half-day) collapses the chances of increasing F&V availability in Market B, irrespective of the other scenarios (Fig. 5).

## 6 Discussion

Navigating trade-offs in food systems is critical to equitably providing nutritious food for all (Kanter et al., 2018; Kanter et al., 2015). Here we have developed a novel modelling framework to identify ‘win-win-wins’ for horticultural value chains, including those between broad livelihood and food security outcomes. We consider the practical implications of this framework for agricultural interventions like Loop, before discussing its methodological transferability and limitations.

### 6.1 Practical implications of the framework

Simply increasing the affordability (i.e. transport subsidies) and volumes (i.e. Loop adoption) of horticultural aggregations may not automatically translate into multidimensional benefits, but instead reinforce the pre-existing governance structures and urban-centric flows of F&V to the detriment of availability in small, access-limited markets. Therefore, the pathways that achieve positive outcomes for multiple value chain actors are inherently complex, involving multidimensionality, nonlinearities and multifinality (i.e. where the same scenario trajectory may produce multiple trade-off outcomes).

Consistent with Giller et al. (2011) and Kanter et al. (2018), identifying and managing key system feedbacks are critical to avoid reinforcing problematic behaviours that already exist within value chains (e.g. the preference to supply large markets). Consequently, we find that interventions that are originally producer-facing may be limited in their leverage, with ‘win-win-win’ futures requiring concurrent changes to the enabling environment to ensure that F&V producers are not financially penalised when increasing supplies towards smaller markets. Similarly, including the implementation costs of governments and private institutions would further widen the scope of this study, as for example, we currently assume that the Bihar state government faces no financial constraints in the delivery of cold storage and efforts to boost baseline F&V demands (e.g. through nutrition awareness programmes).

Exploratory future scenarios show that the chances of different trade-offs occurring are dynamic, dependent upon both the trajectories of individual scenarios and their cross-interactions. For instance, whilst futures with improved local F&V availability may be achieved across the entire range of aggregation transport subsidies, the chances of achieving such futures undergo an abrupt decline as the Loop extension rate gradually increases; such threshold-like dynamics with important food security implications would likely remain hidden if the model was simulated with traditional one-at-a-time scenarios based upon predicting the system's most likely trajectory. From the perspective of development agencies with often limited financial capacities (Stanley et al., 2017), such frameworks provide virtual laboratories to horizon-scan alternative scenarios before implementing more expensive trials in-situ*.* Development practitioners may compare the landscape of trade-offs to their institutional priorities, weighing-up whether to incorporate more value chain actors into their aspirational goals or concentrate on securing positive outcomes for their core participants (e.g. Loop farmers).

To this end, the number of outcome trade-off dimensions could be increased to include any number of actors and interests. For instance, the dimensions analysed here arguably underrepresent the midstream of the chain, with trader numbers and profits being important drivers of F&V distribution (Attwood and Baviskar, 1996). However, increasing the number of targets risks underperformance in one dimension (e.g. F&V availability) being discounted for overperformance in others (e.g. farmer and trader profits) (Holden et al., 2017). Alternatively, simulation outcomes could instead be categorised *post-hoc*, with the use of statistical clustering (Janssen et al., 2012) or multivariable regression techniques empirically analysing the relationships between scenario trajectories and outcomes from the bottom-up. However, the normative futures here help to distinguish the scenarios and feedbacks leading to aspirational futures defined a priori – laying the foundations for future studies to disaggregate outcome dynamics within the ‘win-win-win’ space – such as between farmers with different assets (e.g. land ownership) or consumers with different purchasing capacities.

### 6.2 Transferability and limitations of the framework

To be truly valuable, modelling studies must develop concepts and methodologies that are transferable to other systems and problems (Verburg et al., 2016). In theory, simulating a systems model under a spectrum of exploratory scenarios is transferable to any human or ecological system that can be described as a series of stocks, flows and feedbacks, and where distinct trade-off spaces can be envisioned upfront. However, where decision-makers favour more predictive forecasts (Börjeson et al., 2006), the exploratory elements of this framework can investigate the chances of trade-offs emerging around the most likely system trajectories – in case the system diverges slightly from its intended path. Where data and/or computational resources are limited, qualitative tools such as causal-loop diagrams could be combined with descriptive storylines to explore the range of outcomes and trade-offs emerging under divergent futures (Carpenter et al., 2015; Nicholson et al., 2020; Rammelt and Leung, 2017).

The spatiotemporal dimensions of this framework are also adaptable. Modelling multiple decades could investigate how farmer livelihoods and F&V availability co-evolve under scenarios of climatic shocks, population growth and dietary trends, as well as additional biophysical feedbacks between crop yields and greenhouse gas emissions, soil erosion and groundwater use. However, modelling until 2050 with a half-daily resolution would require ~23,400 timesteps per simulation, making exploratory scenarios and subsequent analysis at the same half-daily resolution unwieldy. Therefore, the temporal horizon is constrained by the desired granularity of the model, which is dependent on data resolution and the dynamics of the underlying problem.

Moreover, this framework could be scaled-up to international trade or scaled-down to village level food systems. For example, at the international scale, the United Nation's FAOSTAT (FAO, 2018) and Comtrade (UN, 2020) databases could provide key numerical datasets on national production quantities, consumption levels and livelihood metrics. Ultimately, adapting this framework going forward requires the relevant stocks, feedbacks, temporal dimensions, and scenarios to be aligned with the system under study, although the underlying philosophy of finding ‘win-win-wins’ would remain the same.

The need to aggregate processes such as individual F&V types and the market preferences of farmers must be traded-off against lighter data demands, shorter model runtimes and increased user-friendliness. Moreover, a single model removes the need to integrate multiple platforms – often written in different computer languages and different spatiotemporal scales (Kanter et al., 2018; Voinov and Shugart, 2013). On the flipside, whilst helping to co-develop model boundaries and uncover key feedbacks driving aggregation adoption and market choice, the process of group model building (GMB) contributed to a larger and more complex model than might have otherwise been designed with only quantitative survey and timeseries data. Although the iterative nature of GMB refined the model over time, and provided a greater depth of analyses than conventional approaches, there is an important balance to be struck going forward between (i) including the variety of processes perceived to be important by stakeholders, (ii) numerically expressing intangible processes as accurately as possible (e.g. trust between farmers and traders), and (iii) the tractability of model outcomes for decision-makers (Ghaffarzadegan et al., 2011; Summers et al., 2015). There are also logistical limits to what can be modelled. For example, whilst it is theoretically possible to model the individual flows of even the five-most aggregated Loop F&Vs, technical feasibility is constrained by the availability of data and model complexity.

More broadly, while system dynamics models are often recognised for their ability to capture of feedback loops, nonlinear dynamics and system leverage points (Nicholson et al., 2020; Turner et al., 2016), numerous limitations remain relative to more traditional process-based trade-off models (Kanter et al., 2018). Common in the analysis of landscape trade-offs (Polasky et al., 2008), SDMs struggle to capture spatially-explicit feedbacks such as the co-evolution of land-use, agricultural inputs and long-term productivity (Neuwirth et al., 2015), which might occur under scenarios of production intensification. Spatial GMB (SGMB) data could inform spatial SDM approaches (Neuwirth et al., 2015), although integrating GIS techniques will likely limit the transferability of the framework, the computational ability to run exploratory scenarios and the tractability of the results for stakeholders. Similarly, disaggregating outcomes amongst a population of consumers would require each sub-population (e.g. by wealth quantile) to have its own stock and flow structure. Consequently, the present study stops short of attributing different health outcomes to individuals, instead focusing on the system-wide trends and feedbacks that drive F&V availability in retail-oriented markets. Therefore, this framework does not attempt to replace process-based models, but instead offers an approach to horizon-scan the dynamics that potentially lead to producer versus consumer trade-offs in agricultural value chains.

## 7 Conclusion

Based upon the Loop aggregation scheme in India, we developed a novel modelling framework that traces multidimensional value chain trade-offs back to their spectrum of causal dynamics to identify futures that simultaneously increase farmer profits, F&V availability in small retail-oriented markets and the sustainability of the original scheme. Upgrading the original aggregation scheme and its wider enabling environment leads to producer versus consumers trade-offs in 71% of simulations, whilst ‘win-win-win’ futures require a narrow range of scenarios that improve F&V demand despite higher prices in the small market, whilst minimising the transport subsidies provided to aggregating farmers. Therefore, this study operationalises a transferable approach to identify guardrails for regional food systems which steer clear of major producer versus consumer trade-offs and towards ‘win-win-win’ futures, whilst calling for the greater appreciation of the unintended consequences that plausibly arise from nutrition-focused value chain upgrades.

## Declaration of Competing Interest

The authors declare that they have no known competing financial interests or personal relationships that could have appeared to influence the work reported in this paper.

## Appendix A Supplementary data

Supplementary material Image 1

## References

[bb0005] Afshin A., Sur P.J., Fay K.A., Cornaby L., Ferrara G., Salama J.S., Mullany E.C., Abate K.H., Abbafati C., Abebe Z., Afarideh M., Aggarwal A., Agrawal S., Akinyemiju T., Alahdab F., Bacha U., Bachman V.F., Badali H., Badawi A., Bensenor I.M., Bernabe E., Biadgilign S.K.K., Biryukov S.H., Cahill L.E., Carrero J.J., Cercy K.M., Dandona L., Dandona R., Dang A.K., Degefa M.G., El Sayed Zaki M., Esteghamati A., Esteghamati S., Fanzo J., Farinha C.S. e S., Farvid M.S., Farzadfar F., Feigin V.L., Fernandes J.C., Flor L.S., Foigt N.A., Forouzanfar M.H., Ganji M., Geleijnse J.M., Gillum R.F., Goulart A.C., Grosso G., Guessous I., Hamidi S., Hankey G.J., Harikrishnan S., Hassen H.Y., Hay S.I., Hoang C.L., Horino M., Islami F., Jackson M.D., James S.L., Johansson L., Jonas J.B., Kasaeian A., Khader Y.S., Khalil I.A., Khang Y.-H., Kimokoti R.W., Kokubo Y., Kumar G.A., Lallukka T., Lopez A.D., Lorkowski S., Lotufo P.A., Lozano R., Malekzadeh R., März W., Meier T., Melaku Y.A., Mendoza W., Mensink G.B.M., Micha R., Miller T.R., Mirarefin M., Mohan V., Mokdad A.H., Mozaffarian D., Nagel G., Naghavi M., Nguyen C.T., Nixon M.R., Ong K.L., Pereira D.M., Poustchi H., Qorbani M., Rai R.K., Razo-García C., Rehm C.D., Rivera J.A., Rodríguez-Ramírez S., Roshandel G., Roth G.A., Sanabria J., Sánchez-Pimienta T.G., Sartorius B., Schmidhuber J., Schutte A.E., Sepanlou S.G., Shin M.-J., Sorensen R.J.D., Springmann M., Szponar L., Thorne-Lyman A.L., Thrift A.G., Touvier M., Tran B.X., Tyrovolas S., Ukwaja K.N., Ullah I., Uthman O.A., Vaezghasemi M., Vasankari T.J., Vollset S.E., Vos T., Vu G.T., Vu L.G., Weiderpass E., Werdecker A., Wijeratne T., Willett W.C., Wu J.H., Xu G., Yonemoto N., Yu C., Murray C.J.L. (2019). Health effects of dietary risks in 195 countries, 1990–2017: a systematic analysis for the Global Burden of Disease Study 2017. Lancet.

[bb0010] Attwood D., Baviskar B., Baviskar B., Attwood D. (1996). Viewpoints. Finding the Middle Path: The Political Economy of Cooperation in Rural India.

[bb0015] Bai X., van der Leeuw S., O’Brien K., Berkhout F., Biermann F., Brondizio E.S., Cudennec C., Dearing J., Duraiappah A., Glaser M., Revkin A., Steffen W., Syvitski J. (2016). Plausible and desirable futures in the Anthropocene: a new research agenda. Glob. Environ. Chang..

[bb0020] Bass F.M. (1969). A new product growth for model consumer durables. Manag. Sci..

[bb0025] Berends J., Rich K., Lyne M. (2020). A Pro-poor Approach to Upgrade Agri-food Value Chains in Tanintharyi Region of Myanmar, in: System Dynamics Society: 3rd Asia Pacific Conference. Brisbane, Australia.

[bb0030] Börjeson L., Höjer M., Dreborg K.-H., Ekvall T., Finnveden G. (2006). Scenario types and techniques: towards a user’s guide. Futures.

[bb0035] Campling L., Havice E. (2013). Mainstreaming environment and development at the World Trade Organization? Fisheries subsidies, the politics of rule-making, and the elusive ‘triple win. Environ. Plan. A.

[bb0040] Carpenter S.R., Booth E.G., Gillon S., Kucharik C.J., Loheide S., Mase A.S., Motew M., Qiu J., Rissman A.R., Seifert J., Soylu E., Turner M., Wardropper C.B. (2015). Plausible futures of a social-ecological system: Yahara watershed, Wisconsin, USA. Ecol. Soc..

[bb0045] Census of India (2011). District Census Handbook.

[bb0050] Chapman A., Darby S. (2016). Evaluating sustainable adaptation strategies for vulnerable mega-deltas using system dynamics modelling: rice agriculture in the Mekong Delta’s An Giang Province, Vietnam. Sci. Total Environ..

[bb0055] Choudhury S., Shankar B., Aleksandrowicz L., Tak M., Green R., Harris F., Scheelbeek P., Dangour A. (2020). What underlies inadequate and unequal fruit and vegetable consumption in India? An exploratory analysis. Global Food Secur..

[bb0060] Coleman K., Muhammed S.E., Milne A.E., Todman L.C., Dailey A.G., Glendining M.J., Whitmore A.P. (2017). The landscape model: a model for exploring trade-offs between agricultural production and the environment. Sci. Total Environ..

[bb0065] Cooper G.S., Dearing J.A. (2019). Modelling future safe and just operating spaces in regional social-ecological systems. Sci. Total Environ..

[bb0070] Dangour A.D., Mace G., Shankar B. (2017). Food systems, nutrition, health and the environment. Lancet Planet. Health.

[bb0075] Digital Green (2017). Digital Green’s LOOP: Pooling Technology and Extension Networks for Market Access.

[bb0080] Dizyee K., Baker D., Rich K.M. (2017). A quantitative value chain analysis of policy options for the beef sector in Botswana. Agric. Syst..

[bb0085] Dogliotti S., van Ittersum M., Rossing W.A. (2005). A method for exploring sustainable development options at farm scale: a case study for vegetable farms in South Uruguay. Agric. Syst..

[bb0090] Elliot D., Gibson A., Hitchins R. (2008). Making markets work for the poor: rationale and practice. Enterprise Dev. Microfinance.

[bb0095] FAO (2014). Developing Sustainable Food Value Chains – Guiding Principles.

[bb0100] FAO (2018). FAOSTAT Statistics Database.

[bb0105] Ford A. (2010). Modeling the Environment.

[bb0110] Frank S.M., Webster J., McKenzie B., Geldsetzer P., Manne-Goehler J., Andall-Brereton G., Houehanou C., Houinato D., Gurung M.S., Bicaba B.W., McClure R.W., Supiyev A., Zhumadilov Z., Stokes A., Labadarios D., Sibai A.M., Norov B., Aryal K.K., Karki K.B., Kagaruki G.B., Mayige M.T., Martins J.S., Atun R., Bärnighausen T., Vollmer S., Jaacks L.M. (2019). Consumption of fruits and vegetables among individuals 15 years and older in 28 low- and middle-income countries. J. Nutr..

[bb0115] Ghaffarzadegan N., Lyneis J., Richardson G.P. (2011). How small system dynamics models can help the public policy process. Syst. Dyn. Rev..

[bb0120] Giller K.E., Tittonell P., Rufino M.C., van Wijk M.T., Zingore S., Mapfumo P., Adjei-Nsiah S., Herrero M., Chikowo R., Corbeels M., Rowe E.C., Baijukya F., Mwijage A., Smith J., Yeboah E., van der Burg W.J., Sanogo O.M., Misiko M., de Ridder N., Karanja S., Kaizzi C., K’ungu J., Mwale M., Nwaga D., Pacini C., Vanlauwe B. (2011). Communicating complexity: integrated assessment of trade-offs concerning soil fertility management within African farming systems to support innovation and development. Agric. Syst..

[bb0125] Gillespie S., Poole N., van den Bold M., Bhavani R.V., Dangour A.D., Shetty P. (2019). Leveraging agriculture for nutrition in South Asia: what do we know, and what have we learned?. Food Policy.

[bb0130] GoI (2018). Horticultural Statistics at a Glance 2018.

[bb0135] Grabowski P., Schmitt Olabisi L., Adebiyi J., Waldman K., Richardson R., Rusinamhodzi L., Snapp S. (2019). Assessing adoption potential in a risky environment: the case of perennial pigeonpea. Agric. Syst..

[bb0140] Groot J.C.J., Oomen G.J.M., Rossing W.A.H. (2012). Multi-objective optimization and design of farming systems. Agric. Syst..

[bb0145] Hawkes C., Ruel M.T. (2011). Value chains for nutrition. IFPRI 2020 International Conference “Leveraging Agriculture for Improving Nutrition and Health”.

[bb0150] Holden E., Linnerud K., Banister D. (2017). The imperatives of sustainable development. Sustain. Dev..

[bb0155] ISEES (2018). STELLA Architect: Systems Thinking for Education and Research.

[bb0160] Janssen P., Walther C., Lüdeke M. (2012). Cluster Analysis to Understand Socio-ecological Systems: A Guideline.

[bb0165] Kadiyala S., Joshi P., Dev S., Kumar T., Vyas V. (2012). A nutrition secure India: role of agriculture. Econ. Polit. Wkly..

[bb0170] Kanter R., Walls H.L., Tak M., Roberts F., Waage J. (2015). A conceptual framework for understanding the impacts of agriculture and food system policies on nutrition and health. Food Secur..

[bb0175] Kanter D.R., Musumba M., Wood S.L.R., Palm C., Antle J., Balvanera P., Dale V.H., Havlik P., Kline K.L., Scholes R.J., Thornton P., Tittonell P., Andelman S. (2018). Evaluating agricultural trade-offs in the age of sustainable development. Agric. Syst..

[bb0180] Karlsson L., Naess L.O., Nightingale A., Thompson J. (2018). ‘Triple wins’ or ‘triple faults’? Analysing the equity implications of policy discourses on climate-smart agriculture (CSA). J. Peasant Stud..

[bb0185] Klapwijk C.J., van Wijk M.T., Rosenstock T.S., van Asten P.J.A., Thornton P.K., Giller K.E. (2014). Analysis of trade-offs in agricultural systems: current status and way forward. Curr. Opin. Environ. Sustain..

[bb0190] Kumari M., Singh R.G. (2016). Demand supply and trade prospects of major fruits and vegetables in Bihar. Int. J. Agric. Sci. Res..

[bb0195] Langellier B.A., Kuhlberg J.A., Ballard E.A., Slesinski S.C., Stankov I., Gouveia N., Meisel J.D., Kroker-Lobos M.F., Sarmiento O.L., Caiaffa W.T., Diez Roux A.V. (2019). Using community-based system dynamics modeling to understand the complex systems that influence health in cities: the SALURBAL study. Health Place.

[bb0200] Lie H., Rich K.M., van der Hoek R., Dizyee K. (2018). An empirical evaluation of policy options for inclusive dairy value chain development in Nicaragua: a system dynamics approach. Agric. Syst..

[bb0205] Maestre M., Poole N., Henson S. (2017). Assessing food value chain pathways, linkages and impacts for better nutrition of vulnerable groups. Food Policy.

[bb0210] Massey F.J. (1951). The Kolmogorov-Smirnov test for goodness of fit. J. Am. Stat. Assoc..

[bb0215] Meadows D.H. (2009). Thinking in Systems: A Primer.

[bb0220] Minocha S., Thomas T., Kurpad A.V. (2018). Are ‘fruits and vegetables’ intake really what they seem in India?. Eur. J. Clin. Nutr..

[bb0225] Minten B., Randrianarison L., Swinnen J.F.M. (2009). Global retail chains and poor farmers: evidence from Madagascar. World Dev..

[bb0230] Minten B., Reardon T., Singh K.M., Sutradhar R. (2011). The Potato Value Chain in Bihar: An Assessment and Policy Implications.

[bb0235] Mumba C., Skjerve E., Rich M., Rich K. (2017). Application of system dynamics and participatory spatial group model building in animal health: a case study of East Coast Fever interventions in Lundazi and Monze districts of Zambia. PLoS One.

[bb0240] Neuwirth C., Peck A., Simonović S.P. (2015). Modeling structural change in spatial system dynamics: a Daisyworld example. Environ. Model Softw..

[bb0245] NHB (2015). Horticulture Crops Estimates for the Year 2013-14 and 2014-15 [WWW Document].

[bb0250] NHB (2018). ‘MIS Weekly Report’ dataset [WWW Document]. http://www.nhb.gov.in/OnlineClient/Weekly.aspx.

[bb0255] NHM (2020). Project Report on Cool Chamber 10MT.

[bb0260] Nicholson C.F., Kopainsky B., Stephens E.C., Parsons D., Jones A.D., Garrett J., Phillips E.L. (2020). Conceptual frameworks linking agriculture and food security. Nat. Food.

[bb0265] NIFTEM (2013). Area, Production and Productivity of Fruits and Vegetables in Different States of the Country.

[bb0270] Parish E.S., Hilliard M.R., Baskaran L.M., Dale V.H., Griffiths N.A., Mulholland P.J., Sorokine A., Thomas N.A., Downing M.E., Middleton R.S. (2012). Multimetric spatial optimization of switchgrass plantings across a watershed. Biofuels Bioprod. Biorefin..

[bb0275] Patil S., K Jha A., Sinha A. (2016). Role of financial agencies in integrating small farmers into a sustainable value chain: a synthesis-based on successful value chain financing efforts. Curr. Sci..

[bb0280] Pingali P., Aiyar A., Abraham M., Rahman A. (2019). Transforming Food Systems for a Rising India.

[bb0285] Polasky S., Nelson E., Camm J., Csuti B., Fackler P., Lonsdorf E., Montgomery C., White D., Arthur J., Garber-Yonts B., Haight R., Kagan J., Starfield A., Tobalske C. (2008). Where to put things? Spatial land management to sustain biodiversity and economic returns. Biol. Conserv..

[bb0290] Rahman A. (2012). Characterizing Food Prices in India.

[bb0295] Rammelt C.F., Leung M.W.H. (2017). Tracing the causal loops through local perceptions of rural road impacts in Ethiopia. World Dev..

[bb0300] Rich K.M., Dizyee K. (2016). Policy Options for Sustainability and Resilience in Potato Value Chains in Bihar: A System Dynamics Approach.

[bb0305] Rich K.M., Rich M., Dizyee K. (2018). Participatory systems approaches for urban and peri-urban agriculture planning: the role of system dynamics and spatial group model building. Agric. Syst..

[bb0310] Spear R.C., Hornberger G.M. (1980). Eutrophication in Peel Inlet-II. Identification of critical uncertainties via generalized sensitivity analysis. Water Res..

[bb0315] Srinivasan V., Sanderson M., Garcia M., Konar M., Blöschl G., Sivapalan M. (2017). Prediction in a socio-hydrological world. Hydrol. Sci. J..

[bb0320] Stanley A.C., Willms D., Schuster-Wallace C., Watt S. (2017). From rhetoric to reality: an NGO’s challenge for reaching the furthest behind. Dev. Pract..

[bb0325] Stave K.A., Kopainsky B. (2015). A system dynamics approach for examining mechanisms and pathways of food supply vulnerability. J. Environ. Stud. Sci..

[bb0330] Sterman J.D. (2000). Business Dynamics: Systems Thinking and Modeling for a Complex World.

[bb0335] Summers D.M., Bryan B.A., Meyer W.S., Lyle G., Wells S., McLean J., Moon T., van Gaans G., Siebentritt M. (2015). Simple models for managing complex social–ecological systems: the Landscape Futures Analysis Tool (LFAT). Environ. Model Softw..

[bb0340] Swinnen J.F.M., Maertens M. (2007). Globalization, privatization, and vertical coordination in food value chains in developing and transition countries. Agric. Econ..

[bb0345] Thow A.M., Verma G., Soni Deepa, Soni Divya, Beri D.K., Kumar P., Siegel K.R., Shaikh N., Khandelwal S. (2018). How can health, agriculture and economic policy actors work together to enhance the external food environment for fruit and vegetables? A qualitative policy analysis in India. Food Policy.

[bb0350] Trebbin A., Franz M. (2010). Exclusivity of private governance structures in agrofood networks: Bayer and the food retailing and processing sector in India. Environ. Plan. A.

[bb0355] Turner B., Menendez H.M., Gates R., Tedeschi L., Atzori A. (2016). System dynamics modeling for agricultural and natural resource management issues: review of some past cases and forecasting future roles. Resources.

[bb0360] UN (2020). UN Comtrade. https://comtrade.un.org/.

[bb0365] UNDP (2012). Case Studies of Sustainable Development in Practice: Triple Wins for Sustainable Development.

[bb0370] USDA (2019). India: Grain and Feed Annual Report.

[bb0375] Vanitha S.M., Chaurasia S.N.S., Singh P.M., Naik P.S. (2013). Vegetable Statistics - Technical Bulletin.

[bb0380] Varadharajan K.S., Thomas T., Kurpad A.V. (2013). Poverty and the state of nutrition in India. Asia Pac. J. Clin. Nutr..

[bb0385] Vennix J.A.M., Andersen D.F., Richardson G.P. (1997). Foreword: group model building, art, and science. Syst. Dyn. Rev..

[bb0390] Verburg P.H., Dearing J.A., Dyke J.G., van der Leeuw S., Seitzinger S., Steffen W., Syvitski J. (2016). Methods and approaches to modelling the Anthropocene. Glob. Environ. Chang..

[bb0395] Voinov A., Shugart H.H. (2013). ‘Integronsters’, integral and integrated modeling. Environ. Model Softw..

[bb0400] von loeper W., Musango J., Brent A., Drimie S. (2016). Analysing challenges facing smallholder farmers and conservation agriculture in South Africa: a system dynamics approach. S. Afr. J. Econ. Manag. Sci..

[bb0405] Walters J.P., Archer D.W., Sassenrath G.F., Hendrickson J.R., Hanson J.D., Halloran J.M., Vadas P., Alarcon V.J. (2016). Exploring agricultural production systems and their fundamental components with system dynamics modelling. Ecol. Model..

