# Supplementary material for: Identifying ‘win-win-win’ futures from inequitable value chain trade-offs: A system dynamics approach
Source: Agric Syst. 2021 May;190:103096. doi: 10.1016/j.agsy.2021.103096 (PMC8121761; doi:10.1016/j.agsy.2021.103096)
Supplement: Supplementary file 1 — Supplementary material [file mmc1.docx]

**Supplementary Material**

Contents

**Supplementary Material A: Qualitative parameter assessment**

A.1: Test setup

A.2: Qualitative parameter assessment output table

**Supplementary Material B: Behaviour reproduction**

B.1: Test setup

B.2: Behaviour reproduction output timeseries

**Supplementary Material C: Extreme scenario analysis**

C.1: Test setup

C.2: Extreme scenario output table

C.3: Extreme scenario output timeseries

**Supplmentary Material D: Integration error test**

**Supplmentary Material E: Monte Carlo sensitivity analysis**

E.1: One-at-a-time output table

E.2: One-at-a-time output timeseries

E.3: Kolmogorov-Smirnov tests

**Supplmentary Material F: Model equations and parameter values**

F.1: Scenario Hub

F.2: Farmer Household

F.3: Loop Benefits & Costs

F.4: Market Supply

F.5: Non-Loop Benefits & Costs

F.6: Production & Aggregation

F.7: Retail Demand

**Supplmentary references**

Supplementary Material A: Qualitative parameter assessment

- 1. Test setup

Chapman and Darby (2016) developed a qualitative assessment to assign a reliability score to every model parameter, whereby the spatial applicability and transferability of the underlying data, plus the quantity of evidence and any statistical confidence are each scored out of three (giving a total of 12). The assessment identifies the most qualitatively uncertain variables, with any variable scoring below the threshold (the mean score minus the standard deviation) here submitted for sensitivity analysis in Section 2.3.

Of the 128 parameters assessed, 47 (36.4%) scored beneath the reliability threshold (Table S1). All unreliable parameters were informed by modeller intuition in the absence of reliable survey, Loop dashboard or SGMB data. These parameters fall into four distinct categories: (i) explicit non-Loop parameters, such as F&V supply quantities and investment in land for F&V cultivation; (ii) parameter weightings in Loop decision-making processes, such as the importance of potential profits when deciding which market to supply; (iii) delays and perception times, including the time it takes traders to adjust prices based on supply and demand, and (iv) other investment rates, including the investments made by Loop farmers into enhanced F&V yields.

- 1. Qualitative parameter assessment output table

| Table S1: Output table of the qualitative parameter assessment for the Bihar system dynamics model (SDM) – based on the methodology of Chapman and Darby (2016). The mean average reliability score equals 58.7% (n = 128) and the standard deviation equals 37.6%. The reliability threshold score equals 37.6%, and variables scoring below the threshold are highlighted in red. | | | | | | |
| --- | --- | --- | --- | --- | --- | --- |
| Module | **Sub-module** | **Variable(s)** | **Source(s)** | | **Information type** | **Score (%)** |
| Farmer Household | Farmer population | Extension on | SGMB sessions; Loop dashboard data; personal intuition | | Expert stakeholder knowledge; modeller intuition | 83 |
|  |  | Extension effectiveness | Bass (1969); Loop dashboard data | | Modeller intuition; statistical information | 67 |
|  |  | Adoption fraction | Bass (1969); Loop dashboard data | | Modeller intuition; statistical information | 67 |
|  |  | Contact rate | Bass (1969); Loop dashboard data; MINI household surveys | | Modeller intuition; statistical information | 67 |
|  |  | Koilwar farming population | Indian census 2011 | | Statistical information | 67 |
|  |  | Harvest frequency | MINI household surveys | | Statistical information | 75 |
|  |  | Base disadoption rate | Loop dashboard data | | Statistical information | 58 |
|  |  | Proportion female Loop farmers | Loop dashboard data | | Statistical information | 67 |
|  |  | Proportion female non-Loop farmers | Indian census 2011 | | Statistical information | 67 |
|  | Home consumption | Loop day consumption | MINI household surveys | | Statistical information | 75 |
|  |  | Non-Loop day consumption | MINI household surveys | | Statistical information | 75 |
|  |  | Gender consumption difference | NSSO 2011-2012 | | Statistical information | 83 |
| Production & aggregation | On-farm costs | Loop on-farm costs (x7) | MINI household surveys | | Statistical information | 75 |
|  |  | Loop labour costs (x7) | MINI household surveys | | Statistical information | 75 |
|  |  | non-Loop on-farm costs (x7) | MINI household surveys | | Statistical information | 75 |
|  |  | non-Loop labour costs (x7) | MINI household surveys | | Statistical information | 75 |
|  | Loop land & yield | Rabi & Zaid yield | NIFTEM (2013) and NHB (2015) | | Statistical information | 50 |
|  |  | Kharif yield | NIFTEM (2013) and NHB (2015) | | Statistical information | 50 |
|  |  | Loop random self-supply | SGMB sessions; personal intuition | | Expert stakeholder knowledge; modeller intuition | 83 |
|  |  | Minimum Loop quality | SGMB sessions; personal intuition | | Expert stakeholder knowledge; modeller intuition | 83 |
|  |  | Maximum Loop quality | SGMB sessions; personal intuition | | Expert stakeholder knowledge; modeller intuition | 83 |
|  |  | Loop quality smooth time | SGMB sessions; personal intuition | | Expert stakeholder knowledge; modeller intuition | 83 |
|  | Loop marketable | On farm wastage rate | MINI household surveys | | Statistical information | 75 |
|  |  | F&V given away rate | MINI household surveys | | Statistical information | 75 |
|  |  | Aggregation fee | SGMB sessions | | Expert stakeholder knowledge | 83 |
|  | Short-term satisfaction | Satisfaction perception time | SGMB sessions | | Expert stakeholder knowledge | 83 |
|  | Market preference | Market A cost per km | SGMB sessions; Loop dashboard data | | Expert stakeholder knowledge; statistical information | 83 |
|  |  | Market A distance | Map-based data; SGMB sessions | | Statistical information; expert stakeholder knowledge | 75 |
|  |  | Market B cost per km | SGMB sessions; Loop dashboard data | | Expert stakeholder knowledge; statistical information | 83 |
|  |  | Market B distance | Map-based data; SGMB sessions | | Statistical information; expert stakeholder knowledge | 75 |
|  |  | Aggregation default capacity | SGMB sessions; Loop dashboard data | | Expert stakeholder knowledge; statistical information | 83 |
|  |  | Smooth capacity time | SGMB sessions; personal intuition | | Expert stakeholder knowledge; modeller intuition | 58 |
|  |  | Aggregator capacity standard deviation | SGMB sessions; Loop dashboard data | | Expert stakeholder knowledge; statistical information | 83 |
|  |  | Market preference change time | SGMB sessions; personal intuition | | Expert stakeholder knowledge; modeller intuition | 83 |
|  |  | Market guaranteed sales weight | Personal intuition | | Modeller intuition | 33 |
|  |  | Market profit weight | Personal intuition | | Modeller intuition | 33 |
|  | Non-Loop land & yield | Rabi & Zaid yield | NIFTEM (2013) and NHB (2015) | | Statistical information | 50 |
|  |  | Kharif yield | NIFTEM (2013) and NHB (2015) | | Statistical information | 50 |
|  |  | Staple price | USDA (2019) | | Statistical information | 58 |
|  |  | Minimum non-Loop quality | SGMB sessions; personal intuition | | Expert stakeholder knowledge; modeller intuition | 83 |
|  |  | Maximum non-Loop quality | SGMB sessions; personal intuition | | Expert stakeholder knowledge; modeller intuition | 83 |
|  |  | Non-Loop quality smooth time | SGMB sessions; personal intuition | | Expert stakeholder knowledge; modeller intuition | 83 |
|  | Non-Loop marketable | On farm wastage rate | MINI household surveys | | Statistical information | 75 |
|  |  | F&V given away rate | MINI household surveys | | Statistical information | 75 |
|  |  | Non-Loop Market A proportion | Personal intuition | | Modeller intuition | 33 |
|  |  | Non-Loop Market B proportion | Personal intuition | | Modeller intuition | 33 |
|  |  | Non-Loop Market A random | Personal intuition | | Modeller intuition | 33 |
|  |  | Non-Loop Market B random | Personal intuition | | Modeller intuition | 33 |
| Market supply: Market A | Large distance market | Large distance market price | National Horticultural Board (NHB) ‘MIS Weekly Report’ dataset | | Statistical information | 50 |
|  |  | Distance trader margin | SGMB sessions | | Expert stakeholder knowledge | 83 |
|  | Trader preference | Price perception time (Loop and non-Loop same variable) | Personal intuition | | Modeller intuition | 33 |
|  |  | Trust perception time (Loop and non-Loop same variable) | Personal intuition | | Modeller intuition | 33 |
|  | Distance trader | Distance traders | SGMB sessions | | Expert stakeholder knowledge | 83 |
|  |  | Distance trader capacity | SGMB sessions; MINI value chain surveys | | Expert stakeholder knowledge; statistical information | 75 |
|  |  | Distance trader wastage rate | MINI value chain surveys | | Statistical information | 67 |
|  |  | Inventory coverage perception time | Value chain assessment and SGMB | | Expert stakeholder knowledge | 83 |
|  |  | Sensitivity of price to inventory coverage | Sterman (2000); personal intuition | | Modeller intuition | 33 |
|  |  | Sensitivity of price to costs | Sterman (2000); personal intuition | | Modeller intuition | 33 |
|  |  | Trader cost perception time | Personal intuition | | Modeller intuition | 33 |
|  |  | Quality price difference | SGMB sessions; MINI value chain surveys | | Expert stakeholder knowledge; statistical information | 75 |
|  |  | Profit perception time | Personal intuition | | Modeller intuition | 33 |
|  | Local trader | Local traders | SGMB sessions | | Expert stakeholder knowledge | 83 |
|  |  | Local trader capacity | SGMB sessions; MINI value chain surveys | | Expert stakeholder knowledge; statistical information | 75 |
|  |  | Local trader wastage rate | MINI value chain surveys | | Statistical information | 67 |
|  |  | Inventory coverage perception time | Value chain assessment and SGMB | | Expert stakeholder knowledge | 83 |
|  |  | Sensitivity of price to inventory coverage | Sterman (2000); personal intuition | | Modeller intuition | 33 |
|  |  | Sensitivity of price to costs | Sterman (2000); personal intuition | | Modeller intuition | 33 |
|  |  | Quality price difference | SGMB sessions; MINI value chain surveys | | Expert stakeholder knowledge; statistical information | 75 |
|  |  | Profit perception time | Personal intuition | | Modeller intuition | 33 |
|  | Local retailer | Retailers | SGMB sessions | | Expert stakeholder knowledge | 83 |
|  |  | Local retailer capacity | SGMB sessions; MINI value chain surveys | | Expert stakeholder knowledge; statistical information | 75 |
|  |  | Local retailer wastage rate | MINI value chain surveys | | Statistical information | 67 |
|  |  | Inventory coverage perception time | Value chain assessment and SGMB | | Expert stakeholder knowledge | 83 |
|  |  | Sensitivity of price to inventory coverage | Sterman (2000); personal intuition | | Modeller intuition | 33 |
|  |  | Sensitivity of price to costs | Sterman (2000); personal intuition | | Modeller intuition | 33 |
|  |  | Quality price difference | SGMB sessions; MINI value chain surveys | | Expert stakeholder knowledge; statistical information | 75 |
|  |  | Profit perception time | Personal intuition | | Modeller intuition | 33 |
|  | Gaddidar commission | Gaddidar outside actor commission | SGMB sessions; MINI value chain surveys | | Expert stakeholder knowledge; statistical information | 75 |
|  |  | Gaddidar local actor commission | SGMB sessions; MINI value chain surveys | | Expert stakeholder knowledge; statistical information | 75 |
| Market supply: Market B | Trader preference | Price perception time | Personal intuition | | Modeller intuition | 33 |
|  |  | Trust perception time | Personal intuition | | Modeller intuition | 33 |
|  | Local trader | Local traders | SGMB sessions | | Expert stakeholder knowledge | 83 |
|  |  | Local trader capacity | SGMB sessions; MINI value chain surveys | | Expert stakeholder knowledge; statistical information | 75 |
|  |  | Local trader wastage rate | MINI value chain surveys | | Statistical information | 67 |
|  |  | Inventory coverage perception time | Value chain assessment and SGMB | | Expert stakeholder knowledge | 83 |
|  |  | Sensitivity of price to inventory coverage | Sterman (2000); personal intuition | | Modeller intuition | 33 |
|  |  | Sensitivity of price to costs | Sterman (2000); personal intuition | | Modeller intuition | 33 |
|  |  | Trader cost perception time | Personal intuition | | Modeller intuition | 33 |
|  |  | Quality price difference | SGMB sessions; MINI value chain surveys | | Expert stakeholder knowledge; statistical information | 75 |
|  |  | Profit perception time | Personal intuition | | Modeller intuition | 33 |
|  | Local retailer | Retailers | SGMB sessions | | Expert stakeholder knowledge | 83 |
|  |  | Local retailer capacity | SGMB sessions; MINI value chain surveys | | Expert stakeholder knowledge; statistical information | 75 |
|  |  | Local retailer wastage rate | MINI value chain surveys | | Statistical information | 67 |
|  |  | Inventory coverage perception time | Value chain assessment and SGMB | | Expert stakeholder knowledge | 83 |
|  |  | Sensitivity of price to inventory coverage | Sterman (2000); personal intuition | | Modeller intuition | 33 |
|  |  | Sensitivity of price to costs | Sterman (2000); personal intuition | | Modeller intuition | 33 |
|  |  | Quality price difference | SGMB sessions; MINI value chain surveys | | Expert stakeholder knowledge; statistical information | 75 |
|  |  | Profit perception time | Personal intuition | | Modeller intuition | 33 |
|  | Gaddidar commission | Gaddidar local actor commission | SGMB sessions; MINI value chain surveys | | Expert stakeholder knowledge; statistical information | 75 |
| Retail demand | Market A demand | Market A retail consumers | MINI value chain surveys | | Statistical information | 67 |
|  |  | Demand adjustment delay | Personal intuition | | Modeller intuition | 33 |
|  |  | Reference industry demand elasticity | Kumari and Singh (2016); Kumar et al. (2011) | | Statistical information | 50 |
|  |  | Maximum consumption | Personal intuition | | Modeller intuition | 33 |
|  |  | Reference price | Personal intuition | | Modeller intuition | 33 |
|  |  | Reference consumer demand | NSSO 2011-2012 | | Statistical information | 83 |
|  |  | Market A customer routine | SGMB sessions; value chain analysis report | | Expert stakeholder knowledge | 66 |
|  | Market B demand | Market B retail consumers | MINI value chain surveys | | Statistical information | 67 |
|  |  | Demand adjustment delay | Personal intuition | | Modeller intuition | 33 |
|  |  | Reference industry demand elasticity | Kumari and Singh (2016); Kumar et al. (2011) | | Statistical information | 50 |
|  |  | Maximum consumption | Personal intuition | | Modeller intuition | 33 |
|  |  | Reference price | Personal intuition | | Modeller intuition | 33 |
|  |  | Reference consumer demand | NSSO 2011-2012 | | Statistical information | 83 |
|  |  | Market B customer routine | SGMB sessions; value chain analysis report | | Expert stakeholder knowledge | 66 |
| Loop benefits and costs | Benefits Market A | All variables are internally derived (i.e. do not rely on external data sources or equations) | | | | |
|  | Benefits Market B | Loop yield investment rate | Personal intuition | | Modeller intuition | 33 |
|  |  | Loop yield investment conversion | Personal intuition | | Modeller intuition | 33 |
|  |  | Loop land investment rate | Personal intuition | | Modeller intuition | 33 |
|  | Trust and utility | Trust perception time | SGMB sessions; personal intuition | | Expert stakeholder knowledge; modeller intuition | 75 |
|  |  | Loop utility perception time | Personal intuition | | Modeller intuition | 33 |
|  |  | Profit utility weight | Personal intuition | | Modeller intuition | 33 |
|  |  | Sales utility weight | Personal intuition | | Modeller intuition | 33 |
|  |  | Loop profit weight in trust | Personal intuition | | Modeller intuition | 33 |
|  |  | Loop sales weight in trust | Personal intuition | | Modeller intuition | 33 |
| Non-Loop benefits and costs | Benefits Market A | All variables are internally derived (i.e. do not rely on external data sources or equations) | | | | |
|  | Benefits Market B | Non-Loop yield investment rate morning | | Personal intuition | Modeller intuition | 33 |
|  |  | Non-Loop yield investment rate afternoon | | Personal intuition | Modeller intuition | 33 |
|  |  | Non-Loop yield investment conversion morning | | Personal intuition | Modeller intuition | 33 |
|  |  | Non-Loop yield investment conversion afternoon | | Personal intuition | Modeller intuition | 33 |
|  |  | Non-Loop land investment rate morning | | Personal intuition | Modeller intuition | 33 |
|  |  | Non-Loop land investment rate afternoon | | Personal intuition | Modeller intuition | 33 |

Supplmentary Material B: Behaviour reproduction

B.1 Test setup

Behaviour reproduction aims to understand the disparity between the model and reality. To this end, Loop dashboard data from October 2017 to February 2018 was reserved for model design (Section 2.2), before data from March to August 2018 assessed model fit.

Ultimately, system dynamics models are not intended for point-to-point prediction (Sterman, 2000), meaning some discrepancy between model and reality should be expected. Moreover, due to co-dependence, non-stationarity and non-normality, SDM timeseries are ill-suited to conventional goodness of fit metrics (versus empirical datasets) such as the coefficient of determination (Barlas, 1989). Instead, SDMs should capture major timeseries trends, including cycles of growth and decline, mean averages and change points. Considering data availability in the Loop dashboard, we visualise the total number of farmers in Koilwar registered to Loop, the number supplying Loop each day and the Loop quantities supplied to Market A.

Within the model itself, Loop extension activities are switched-off from 15^th^ March 2018. The modelled total number of Loop farmers in Koilwar is almost an exact replica of the empirical data (Figure S1A), building confidence that the model accurately captures the timing and magnitudes of Loop adoption. The modelled number of farmers supplying Loop each day also tracks the empirical data between March – August 2018 (Figure S1B) (mean modelled = 73.4 farmers/day; mean observed = 74.0 farmers/day).

Moreover, whilst the model estimates the mean Loop supply to Market A to within 0.7% (mean modelled = 21,900 kg/day, mean observed = 21,700 kg/day), the model does not capture the day-to-day variations (Figure S1C). This is a limitation of deterministic modelling more generally, as the model does not capture all external sources of apparent randomness (e.g. weather conditions, traffic jams or random human decisions). Consequently, the modelled supply of F&V is closer to a moving average – capturing the major periods of growth, stability and decline. This tendency is positive from a modelling standpoint, as outcomes should be traceable to driver dynamics rather than stochastic variations (Voinov and Shugart, 2013).

| 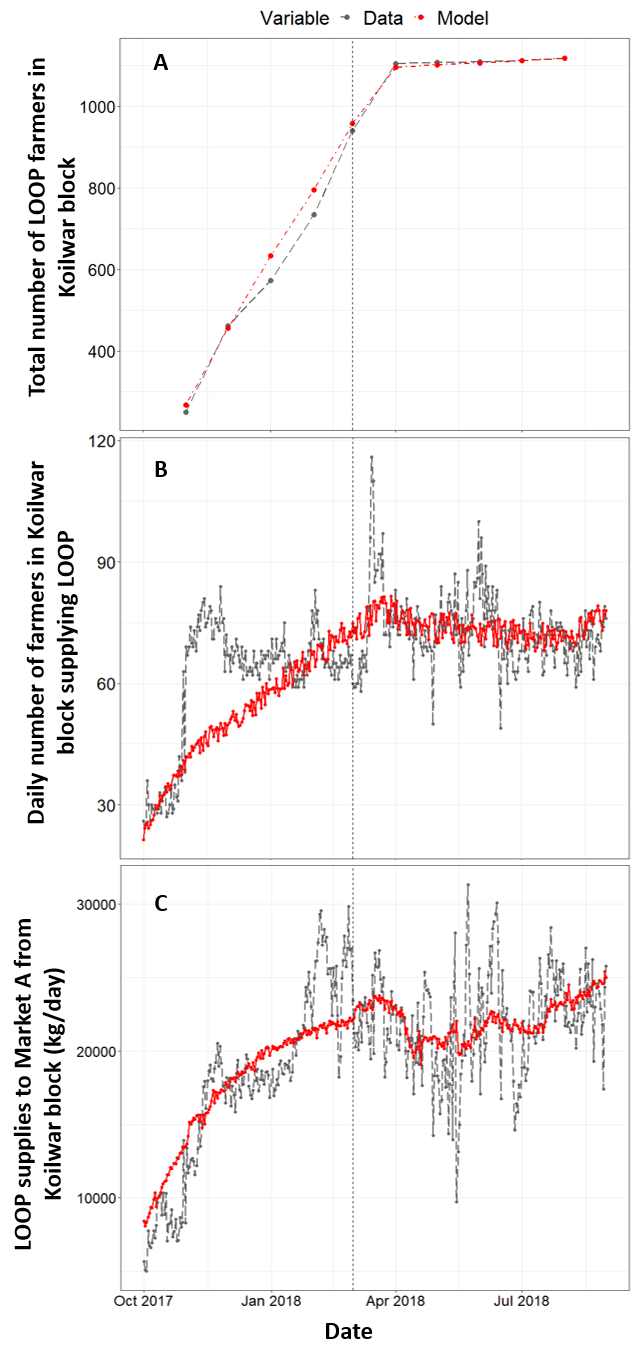 |
| --- |
| Figure S1: Timeseries comparing modelled outputs to Loop dashboard data: (a) the number of Loop farmers in Koilwar block, (b) the number of farmers in Koilwar block supplying Loop each day, and (c) the quantity of Loop F&V supplied to Market A from Koilwar block. The dashed line separates the parameterisation period (left) from the evaluation period (right). |

B.2 Behaviour reproduction output timeseries

Supplmentary Material C: Extreme scenario analysis

C.1 Test setup

Given the range of feedbacks, data sources and equations in the model, it is possible for errors to creep in that only become exposed in the extreme regions of parameter space. This performance analysis test is designed to assess whether model integrity remains when inputs are ‘switched off’ or maximised (Sterman, 2000). For example, setting F&V yields to 0 kg/katha/half-day but finding that Loop produce is still being sold one month later would suggest the presence of a spurious variable/equation that is artificially inflating or delaying market supplies.

We ran 24 scenarios by simulating 12 external variables (i.e. not involved in any feedbacks) under extreme ‘low’ and extreme ‘high’ conditions. The effects were then observed on four key outcome variables: (i) total Loop market sales, (ii) the retail price of F&V in Market B, (iii) Loop revenues, and (iv) cumulative F&V purchases by consumers in Market B. Model outcomes should remain realistic and avoid crashing the model. We go one-step further by hypothesising the likely output dynamics prior to the simulation, before explaining any discrepancies between our prediction and the model outcome.

All of the 96 output dynamics (12 external parameters $\times$ 2 extreme scenarios $\times$ 4 outcome variables) are realistic and uphold the integrity of the model (Table S2 and Figures S2.1-S4.2). We were able to predict 84 outcome dynamics, with 9 outcomes partially predicted and 3 outcomes not predicted prior to the simulations. These incorrect predictions underestimated the sensitivity of non-Loop supplies to the rate of Loop adoption, as opposed to an unforeseeable/unrealistic output from the model. Ultimately, this test builds confidence that the model can be stressed under extreme conditions but still retain structural and quantitative integrity, as well as tractability between model outcomes and drivers.

C.2 Extreme scenario output table

| Table S2: Output table for the extreme scenarios ran to evaluate whether the model’s structures and equations remain robust when subject to extreme conditions. Each of the 12 external parameters are simulated under extreme low and extreme high scenarios, with the resulting dynamics summarised by four output variables (which can be seen in Figures S2.1-S5.2). Hypothesis matched key: ‘✓’ – the output dynamics were successfully predicted prior to the simulation; ‘~’ – the output dynamics were partially predicted prior to the simulation, however surprising dynamics also emerged; ‘🗶’ - the output dynamics were not predicted prior to the simulation. | | | | | | |
| --- | --- | --- | --- | --- | --- | --- |
| **Driving variable** | **Scenario ID** | **Output variable** | **Driver extreme scenario** | **Hypothesised output behaviour** | **Hypothesis matched** | **Explanatory notes** |
| A1. Loop horticultural yield | A1.1 | Total Loop sales | Low (*Loop yield = 0*) | Loop sales to drop to zero almost immediately | ✓ |  |
|  |  |  | High (*Loop yield = 100* $\times$ *reference*) | Loop sales to rapidly increase before potential declining due to market oversaturation | ~ | Dynamics are as hypothesised, except for the decline in sales, which is less significant than predicted. Instead, Loop production is accommodated by an increased number of traders in Market A. |
|  | A1.2 | Retail F&V price in Market B | Low (*Loop yield = 0*) | Slight increase in prices relative to reference mode owing to cessation of Loop supply | ✓ | Mean Market B retail price in extreme low scenario = 24.6 Rs/kg; Mean Market B retail price in reference scenario = 23.2 Rs/kg. |
|  |  |  | High (*Loop yield = 100* $\times$ *reference*) | Drastic price decline, assuming ~10% of Loop supplies continue to Market B | ~ | Dynamics are as hypothesises, except for a temporary recovery of prices after 150 days, driven by Loop farmers moving away from Market B in favour of the higher prices in Market A. |
|  | A1.3 | Loop revenues | Low (*Loop yield = 0*) | Loop revenues drop to zero due to a lack of sales | ✓ |  |
|  |  |  | High (*Loop yield = 100* $\times$ *reference*) | Similar to the total sales output (A1.1), with a decline over time due to market saturation | ✓ |  |
|  | A1.4 | Cumulative F&V purchases per retail customer in Market B | Low (*Loop yield = 0*) | Cumulative purchases may be less than the reference mode due to a reduction in supply and an increase in retail prices | ✓ | We can estimate the final daily per capita F&V consumption for customers in retail Market B assuming that food is evenly split within a household across an average of 5.5 members. Low scenario = 124 g/capita/day; reference scenario = 127 g/capita/day. |
|  |  |  | High (*Loop yield = 100* $\times$ *reference*) | Likely to lead to the steepest cumulative trend due to higher supplies and lower prices. | ✓ | As per the methodology above to estimate the final daily per capita F&V consumption from retail Market B: high scenario F&V consumption = 244 g/capita/day. |
| A2. Loop production quality | A2.1 | Total Loop sales | Low (*all produce is second grade*) | Prices and revenues will be negatively affected by the poor quality supply, potentially causing farmers to disadopt Loop | ✓ | We see a gradual decline in total Loop sales, owing to the inferior performance of the aggregation scheme relative to non-Loop supply pathways. |
|  |  |  | High (*all produce is high grade)* | The produce will demand the best prices from traders, leading to the best revenues and potentially feeding back to positively effect sales (e.g. through F&V land allocation) | ✓ |  |
|  | A2.2 | Retail F&V price in Market B | Low (*all produce is second grade*) | Expect lower prices than the reference mode. However, the difference may be limited by Loop’s small contribution to Market B dynamics (<10% total sales by retailers in Market B) | ✓ | Mean Market B retail price in extreme low scenario = 23.0 Rs/kg; Mean Market B retail price in reference scenario = 23.2 Rs/kg. |
|  |  |  | High (*all produce is high grade)* | Prices expected to be slightly higher owing to the high quality of the produce | ✓ | Mean Market B retail price in extreme high scenario = 23.6 Rs/kg; Mean Market B retail price in reference scenario = 23.2 Rs/kg. |
|  | A2.3 | Loop revenues | Low (*all produce is second grade*) | As per total Loop sales (A2.1), would expect revenues to be less than the reference mode | ✓ |  |
|  |  |  | High (*all produce is high grade)* | Loop revenues to be more than the reference mode | ✓ |  |
|  | A2.4 | Cumulative F&V purchases per retail customer in Market B | Low (*all produce is second grade*) | Assuming prices decrease, we should see a proportionate increase in consumption (albeit that Loop supplies are a small contribution to Market B). | ✓ | Per capita daily F&V consumption by the end of the simulation: Low scenario = 128 g/capita/day; reference scenario = 127 g/capita/day. |
|  |  |  | High (*all produce is high grade)* | The opposite of the above; we might expect a decrease in consumption due to higher prices | ✓ | Per capita daily F&V consumption by the end of the simulation: High scenario = 124 g/capita/day; reference scenario = 127 g/capita/day. |
| A3. Loop adoption rate | A3.1 | Total Loop sales | Low *(no Loop adoption)* | Loop sales will only derive from the initial number of Loop farmers | ~ | The initial Loop farmers are the only farmers to supply the aggregation system, but the total sales actually decline over time as farmers leave the scheme. |
|  |  |  | High (Reference adoption rate $\times$100 | Loop sales will dramatically increase, potentially until all F&V production in Koilwar block is captured. The trend may then decline over time as farmers disadopt Loop | ✓ |  |
|  | A3.2 | Retail F&V price in Market B | Low *(no Loop adoption)* | A slight decrease in F&V prices, as the lack of Loop adoption increases the supply of F&V to Market B (relative to reference mode preference for Market A) | ✓ | Mean Market B retail price in extreme low scenario = 22.3 Rs/kg; Mean Market B retail price in reference scenario = 23.2 Rs/kg. |
|  |  |  | High (Reference adoption rate $\times$100 | Assuming that the vast majority of Loop farmers will prefer to supply Market A, we may see a dramatic increase in F&V prices in Market B (with all non-Loop farmers adopting Loop) | ✓ | Mean Market B retail price in extreme low scenario = 51.9 Rs/kg; Mean Market B retail price in reference scenario = 23.2 Rs/kg. |
|  | A3.3 | Loop revenues | Low *(no Loop adoption)* | Revenue will only be generated by the initial stock of Loop farmers | ✓ | As per A3.1, revenue declines as the original group of farmers gradually disadopt over time. |
|  |  |  | High (*Reference adoption rate* $\times$*100*) | Approximately the same trend as in A3.1 is expected | ✓ |  |
|  | A3.4 | Cumulative F&V purchases per retail customer in Market B | Low *(no Loop adoption)* | We might expect a slight increase in purchases based on the hypothesis of A3.2 | ✓ | Per capita daily F&V consumption by the end of the simulation: Low scenario = 135 g/capita/day; reference scenario = 127 g/capita/day. |
|  |  |  | High (*Reference adoption rate* $\times$*100*) | Based on the hypothesis of A3.2, we might see a shallow trend and a reduction in the per capita daily F&V consumption relative to the reference mode | ✓ | Per capita daily F&V consumption by the end of the simulation: High scenario = 6.6 g/capita/day (from Market B only); reference scenario = 127 g/capita/day. |
| A4. Loop market transport costs | A4.1 | Total Loop sales | Low (*transport costs = 0 Rs*) | May be a slight increase in sales, as supplying both markets will become more affordable, and increased profits may feedback into land allocation, yield investment and F&V production | ✓ | Average daily sales are 0.06% higher under the extreme low scenario than the reference mode, suggesting the feedbacks with production had little effect over the simulation horizon (i.e. less than an entire year). |
|  |  |  | High (*Reference transport costs* $\times$*100*) | Would expect a decline in the total Loop sales, although perhaps not immediately, due to the in-built delays that resist farmers disadopting Loop at the first opportunity | ✓ | As hypothesised, the decline in Loop sales does not happen immediately, as Loop farmers consider both their profits (which have collapsed under the extreme cost scenario) *and* the ability to sell all of their produce through Loop (which remains high). Eventually, the lost profits outweigh the benefits of remaining, so the number of Loop farmers fall to 0. |
|  | A4.2 | Retail F&V price in Market B | Low (*transport costs = 0 Rs*) | The impact might be marginal here, as it becomes cheaper to supply both markets, so the model is unlikely to produce an abrupt swing to/from Market B | ✓ | Mean Market B retail price in extreme low scenario = 23.5 Rs/kg; Mean Market B retail price in reference scenario = 23.2 Rs/kg. |
|  |  |  | High (*Reference transport costs* $\times$*100*) | As above: no major change expected, although we may see a slight price decline as farmers leave Loop (and consequently increase supplies to Market B) | ✓ | Mean Market B retail price in extreme low scenario = 23.1 Rs/kg; Mean Market B retail price in reference scenario = 23.2 Rs/kg. |
|  | A4.3 | Loop revenues | Low (*transport costs = 0 Rs*) | Will reflect Loop membership and total sales. Might expect an increase in revenue if there is an increase in sales (assuming the increase in sales does not negatively impact market prices) | ~ | Average daily sales are 0.25% higher under the extreme low scenario than the reference mode. |
|  |  |  | High (*Reference transport costs* $\times$*100*) | Expect revenue to reflect the sales pattern in A4.1, i.e. potentially decline after a delay | ✓ |  |
|  | A4.4 | Cumulative F&V purchases per retail customer in Market B | Low (*transport costs = 0 Rs*) | Expect marginal changes in line with A4.2 | ✓ | Per capita daily F&V consumption by the end of the simulation: Low scenario = 128 g/capita/day; reference scenario = 127 g/capita/day. |
|  |  |  | High (*Reference transport costs* $\times$*100*) | Expect marginal changes in line with A4.2 | ✓ | Per capita daily F&V consumption by the end of the simulation: High scenario = 128 g/capita/day (from Market B); reference scenario = 127 g/capita/day. |
| A5. Loop on-farm costs | A5.1 | Total Loop sales | Low (*production and labour costs = 0 Rs*) | As hypothesised with transport costs (A4.1), we may see a slight increase in Loop sales, as increased profits feedback to boost production | ~ | The difference is marginal, with the average daily Loop sales in the low extreme scenario equalling < 0.05% more than the reference mode. |
|  |  |  | High (*reference costs* $\times$*100*) | If the costs of Loop participation far outweigh the benefits, we should expect to see a decline in the total Loop sales over time | ✓ | As seen in A4.1, we see a decline in the total number of Loop farmers as the costs of production outweigh the benefits of participation. |
|  | A5.2 | Retail F&V price in Market B | Low (*production and labour costs = 0 Rs*) | Again, as per A4.2, unlikely to have a significant impact, as the costs effect both markets equally | ✓ | Mean Market B retail price in extreme low scenario = 23.1 Rs/kg; Mean Market B retail price in reference scenario = 23.2 Rs/kg. |
|  |  |  | High (*reference costs* $\times$*100*) | Also, as per A4.2, expect to see little changes here, except for perhaps a slight decrease in price as farmers who previously supplied Market A in Loop switch to supplying Market B as part of non-Loop | ✓ | Mean Market B retail price in extreme high scenario = 23.1 Rs/kg; Mean Market B retail price in reference scenario = 23.2 Rs/kg. |
|  | A5.3 | Loop revenues | Low (*production and labour costs = 0 Rs*) | A slight increase in revenues - as per the reasoning in A5.1 | ✓ | As hypothesised, we see a marginal increase in Loop revenue (0.2%) relative to the reference mode. |
|  |  |  | High (*reference costs* $\times$*100*) | If sales dry up, revenues should follow. | ✓ | Loop revenues fall as hypothesised. |
|  | A5.4 | Cumulative F&V purchases per retail customer in Market B | Low (*production and labour costs = 0 Rs*) | Based on A5.2, expect the scenario to have a negligible impact | ✓ | Per capita daily F&V consumption by the end of the simulation: Low scenario = 125 g/capita/day; reference scenario = 127 g/capita/day. |
|  |  |  | High (*reference costs* $\times$*100*) | Same as above; expect negligible impact, assuming there is little change in the retail price in Market B | ✓ | Per capita daily F&V consumption by the end of the simulation: High scenario = 128 g/capita/day (from Market B); reference scenario = 127 g/capita/day. |
| B1. Non-Loop horticultural yield | B1.1 | Total Loop sales | Low (*non-Loop yield = 0*) | We hypothesise the effect of non-Loop yield to be the opposite of Loop yield (A1.1). Therefore, an extremely low non-Loop yield might lead to an increase in Loop sales, as more non-Loop farmers are attracted to Loop practices/practices | ✓ |  |
|  |  |  | High (*non-Loop yield = 100* $\times$ *reference*) | In contrast, if non-Loop yield is extremely high, we expect farmers to disadopt Loop and opt for non-Loop practices/pathways | ✓ |  |
|  | B1.2 | Retail F&V price in Market B | Low (*non-Loop yield = 0*) | As non-Loop supplies dominate Market B dynamics, the disappearance of non-Loop production should have a positive impact on Market B retail prices | ✓ | As hypothesised, retail prices in Market B increase (relative to the reference mode), although the increase is limited somewhat by the heightened adoption of Loop.  Mean Market B retail price in extreme low scenario = 33.4 Rs/kg; Mean Market B retail price in reference scenario = 23.2 Rs/kg. |
|  |  |  | High (*non-Loop yield = 100* $\times$ *reference*) | This scenario is likely to flood Market B with extra F&V supplies, leading to a collapse in the retail price | ✓ | As hypothesised, the over-supply of F&V to Market B causes the retail price to abruptly collapse. At times, the non-Loop supply to Market B exceeds 200,000 kg/day, while the initial market capacity is ~20,000 kg/day.  Mean Market B retail price in extreme high scenario = 0.20 Rs/kg; Mean Market B retail price in reference scenario = 23.2 Rs/kg. |
|  | B1.3 | Loop revenues | Low (*non-Loop yield = 0*) | Expect a similar trend to B1.1: i.e. increase in Loop revenue relative to the reference mode | ✓ | This scenario produces daily revenues that are on average three times that of the reference mode. However, this figure is skewed towards the first 100 timesteps, when Loop farmers generate massive profits from the high prices (owing to the disappearance of non-Loop supplies). |
|  |  |  | High (*non-Loop yield = 100* $\times$ *reference*) | Again, in-line with B1.1, we expect revenues to fall to zero as farmers disadopt Loop | ✓ |  |
|  | B1.4 | Cumulative F&V purchases per retail customer in Market B | Low (*non-Loop yield = 0*) | As per B1.2, the cessation of non-Loop supplies and the associated increase in Market B retail prices should have a dampening effect on F&V purchases | ✓ | Per capita daily F&V consumption by the end of the simulation: Low scenario = 107 g/capita/day; reference scenario = 127 g/capita/day. |
|  |  |  | High (*non-Loop yield = 100* $\times$ *reference*) | Oppositely, flooding Market B with non-Loop produce should lead to more affordable F&V and higher purchase rates | ✓ | Per capita daily F&V consumption by the end of the simulation: High scenario = 264 g/capita/day; reference scenario = 127 g/capita/day. |
| B2. Non-Loop production quality | B2.1 | Total Loop sales | Low (*all produce is second grade*) | Likely to lead to slightly higher Loop sales, as Loop’s superior quality may lead to higher prices and profits, which will feedback into production. | ✓ | This scenario produces a 4.5% increase in the daily average Loop sales relative to the reference mode |
|  |  |  | High (*all produce is high grade)* | The opposite effect; non-Loop’s quality advantage may dampen the benefits gained from Loop | ✓ | This scenario causes a -0.3% change in the daily average Loop sales relative to the reference mode |
|  | B2.2 | Retail F&V price in Market B | Low (*all produce is second grade*) | As non-Loop supply is a significant contribution to Market B, a lower quality standard will likely reduce the retail price relative to the reference mode | 🗶 | The extreme low scenario averages 25.7 Rs/kg and the reference mode averages 23.2 Rs/kg. Whilst the output is certainly reasonable, the hypothesis did not factor in the reduced non-Loop supplies to Market B (as farmers adopt Loop) – which helps to limit the price reduction relative to the reference mode. |
|  |  |  | High (*all produce is high grade)* | Opposite of the above; better non-Loop quality should produce higher retail prices in Market B | ✓ | The extreme low scenario averages 23.5 Rs/kg and the reference mode averages 23.2 Rs/kg. |
|  | B2.3 | Loop revenues | Low (*all produce is second grade*) | As per B2.1, Loop revenues may be boosted due to the superior quality of F&V supplies relative to non-Loop | ✓ | This scenario produces a 7.7% increase in the daily average Loop revenue relative to the reference mode |
|  |  |  | High (*all produce is high grade)* | Opposite to the above, Loop revenues may be dampened if non-Loop supplies all high grade | ✓ | This scenario causes a -2.0% change in the daily average Loop sales relative to the reference mode |
|  | B2.4 | Cumulative F&V purchases per retail customer in Market B | Low (*all produce is second grade*) | As per the hypothesis of B2.2 (low), a significant reduction in the retail price should cause a positive purchase response | 🗶 | In line with the outcome of B2.2 (low), there is actually a small negative effect on cumulative F&V purchases, as the hypothesis did not factor in the reduced non-Loop supplies to Market B (as farmers adopt Loop)  Per capita daily F&V consumption by the end of the simulation: Low scenario = 122 g/capita/day; reference scenario = 127 g/capita/day. |
|  |  |  | High (*all produce is high grade)* | Opposite to the above, an increase in F&V price should reduce F&V purchases relative to the reference mode | 🗶 | As per B2.2 (high), the hypothesis here did not factor in the competitive advantage of non-Loop produce leading to a slight increase in the non-Loop volumes supplied to Market B.  Per capita daily F&V consumption by the end of the simulation: High scenario = 127 g/capita/day; reference scenario = 127 g/capita/day. |
| C1. External market price | C1.1 | Total Loop sales | Low (*price* = 0.01 $\times$ *reference*) | Supplying Market A would not be profitable, so supplies would switch to Market B. However, the shift is likely to outpace the change in traders in Market B, leading to unsold Loop produce. | ✓ | Loop supplies peak at 17,400 kg/day, before overshooting the capacity of Market B. Farmers then gradually disadopt Loop as a consequence of the undesirable wastage rates. |
|  |  |  | High (*price = 100* $\times$ *reference*) | The opposite is likely to happen. The total sales and revenues generated are likely to rapidly increase. Supplies may continue to increase as F&V cultivation becomes hyper profitable; however, there is the possibility that supplies from Loop and non-Loop will overshoot the market capacities if the number of traders keeps pace. | ✓ | Sales rapidly increase as Loop participation becomes hugely profitable. However, the production response causes local trader and retailer prices to collapse, thus limiting the profits gained from F&V cultivation. |
|  | C1.2 | Retail F&V price in Market B | Low (*price* = 0.01 $\times$ *reference*) | Increased supplies to Market B might dampen the retail price initially. However, prices may then increase if Loop participation falls | ✓ | Mean Market B retail price in extreme low scenario = 24.0 Rs/kg; Mean Market B retail price in reference scenario = 23.2 Rs/kg. |
|  |  |  | High (*price = 100* $\times$ *reference*) | The price in Market B is likely to start high (i.e. in line with the scenario set up), but then fall as a result of two processes: (i) the over-supply of Market A and the resulting spillover affects on Market B, and (ii) re-adjustment to the affordable price range for consumers in Market B. | ✓ | As predicted, although the average price in the extreme scenario remains higher than the reference mode (owing to the extreme price for the first ~20 days):  Mean Market B retail price in extreme low scenario = 83.7 Rs/kg; Mean Market B retail price in reference scenario = 23.2 Rs/kg |
|  | C1.3 | Loop revenues | Low (*price* = 0.01 $\times$ *reference*) | Start low and fall towards zero (depending on the sales trend) | ✓ | As predicted, revenue for the first 100 timesteps equals 10% of the reference, before following the steady trajectory of the sales dynamic (see C1.1). |
|  |  |  | High (*price = 100* $\times$ *reference*) | As per the sales hypothesis (C1.1), revenues are likely to rapidly increase. However, they may then decline if the effect of market over-supply causes a collapse in the price of F&V in Market A and B | ✓ |  |
|  | C1.4 | Cumulative F&V purchases per retail customer in Market B | Low (*price* = 0.01 $\times$ *reference*) | Assuming prices remain lower than the reference mode, cumulative purchases from retailers in Market B are likely to be higher than the reference mode by the end of the simulation. | ~ | The hypothesis did not account for the loss of supplies to Market B producing a slightly higher average price (see C1.2)  Per capita daily F&V consumption by the end of the simulation: Low scenario = 123 g/capita/day; reference scenario = 127 g/capita/day |
|  |  |  | High (*price = 100* $\times$ *reference*) | If prices start extremely high, cumulative purchases are likely to be below the reference mode. However, cumulative purchases may rapidly increase if oversupply causes prices to collapse. | ✓ | Per capita daily F&V consumption by the end of the simulation: High scenario = 200 g/capita/day; reference scenario = 127 g/capita/day. |
| C2. Market A capacity  (Daily capacities of distance traders, local traders and retailers) | C2.1 | Total Loop sales | Low (*combined capacity = 0 kg/day*) | Expect Loop sales to be constrained by the absence of Market A from the value chain. Whilst sales might continue in Market B, at least temporarily, the loss of Market A is likely to lead to a reduction in Loop sales and participation over time. | ✓ |  |
|  |  |  | High (*combined capacity = 100* $\times$ *reference*) | Expect the buying prices offered by traders to increase dramatically, as the gap between supply and demand will widen dramatically. This will then enhance profits generated by Loop farmers, which will boost investment into F&V land, yield and ultimately sales. The unknown here is whether production may eventually overshoot the new market capacities. | ✓ | As hypothesised, trader prices steeply rise, with local traders in Market A offering 10,000 Rs/kg by time-step 330 due to the imbalance between supply and demand. Moreover, the farmer profits trigger a step-wise change in the supply quantities flowing towards Market A and Market B. |
|  | C2.2 | Retail F&V price in Market B | Low (*combined capacity = 0 kg/day*) | The prices in Market B are likely to decline, owing to the increase in supplies due to the effective closure of Market A. | ✓ | Mean Market B retail price in extreme low scenario = 21.8 Rs/kg; Mean Market B retail price in reference scenario = 23.2 Rs/kg. |
|  |  |  | High (*combined capacity = 100* $\times$ *reference*) | Here the prices are likely to increase, owing to the influence of higher prices in Market A and the diversion of supplies towards Market A (due to the supply and demand imbalance). However, in line with C2.1, prices may eventually crash if the production response leads to an overshoot of the new market capacity. | ✓ | Caused by the capacity overshoot, the retail price in Market B falls from 68 Rs/kg to 0.5 Rs/kg between simulation days 124 and 155. |
|  | C2.3 | Loop revenues | Low (*combined capacity = 0 kg/day*) | Owing to the closure of Market A, revenues generated from only supplying Market B are likely to be weaker than the reference. | ✓ | This scenario produces a -75.0% change in the daily average Loop revenue relative to the reference mode |
|  |  |  | High (*combined capacity = 100* $\times$ *reference*) | Function of the supply and price trends. Expect an increase in the revenue, potentially followed by an abrupt collapse in case of any capacity overshoot. | ✓ | Loop revenues reach 650-million Rs/day by time-step 153, before collapsing in line with C2.1 and C2.2. |
|  | C2.4 | Cumulative F&V purchases per retail customer in Market B | Low (*combined capacity = 0 kg/day*) | Likely to lead to an increase in purchases relative to the reference mode, owing to greater supplies to Market B and lower prices. | ✓ | Per capita daily F&V consumption by the end of the simulation: Low scenario = 137 g/capita/day; reference scenario = 127 g/capita/day. |
|  |  |  | High (*combined capacity = 100* $\times$ *reference*) | May see a more complex pattern here. Purchases may be initially stifled by extremely high prices, although any subsequent price collapse may lead to a sudden increase in purchases. | ✓ | Per capita daily F&V consumption by the end of the simulation: High scenario = 127 g/capita/day; reference scenario = 127 g/capita/day. |
| C3. Market B capacity | C3.1 | Total Loop sales | Low (*combined capacity = 0 kg/day*) | Expect to see slightly dampened sales relative to the reference mode, as the majority of Loop supplies continue to be routed towards Market A. | ✓ | This scenario produces a -4.8% decrease in the daily average Loop sales relative to the reference mode |
|  |  |  | High (*combined capacity = 100* $\times$ *reference*) | As per C2.1, total sales are likely to be higher than the reference mode. However, the effect is likely to be less pronounced, as Market B is initially less important to Loop than Market A. Again, we may see an overshoot effect if production exceeds the combined market capacities, leading to a decline in Loop sales over time. | ✓ | The decline in Loop sales is driven by a retraction in Loop participation. Whilst non-Loop farmers are benefiting from Market B, Loop farmers are relatively slow to move away from their trusted Market A, producing lower average profits than non-Loop farmers. |
|  | C3.2 | Retail F&V price in Market B | Low (*combined capacity = 0 kg/day*) | Owing to the lack of Market B demand, we would expect to see lower prices than the reference. | ✓ | Mean Market B retail price in extreme low scenario = 11.0 Rs/kg; Mean Market B retail price in reference scenario = 23.2 Rs/kg. |
|  |  |  | High (*combined capacity = 100* $\times$ *reference*) | In contrast to the above, we expect to see higher prices than the reference mode. However, the price trend may fluctuate, in cases where market supplies overshoot demand. | ✓ | Mean Market B retail price in extreme high scenario = 62.1 Rs/kg; Mean Market B retail price in reference scenario = 23.2 Rs/kg. |
|  | C3.3 | Loop revenues | Low (*combined capacity = 0 kg/day*) | In line with sales and associated prices, we expect revenues to be slightly less than the reference mode (but not to the extent of C2.3). | ✓ | This scenario produces a -2.2% change in the daily average Loop revenue relative to the reference mode |
|  |  |  | High (*combined capacity = 100* $\times$ *reference*) | Expect to see a relatively spiky trend here, with revenues spiking during times of high sales and high prices in Market B, but falling if/when capacities are overshot. | ✓ | This scenario produces a 630% increase in the daily average Loop revenue relative to the reference mode. |
|  | C3.4 | Cumulative F&V purchases per retail customer in Market B | Low (*combined capacity = 0 kg/day*) | Owing to the lack of retailers in Market B, F&V purchases in this scenario should equal 0 g/day. | ✓ | Per capita daily F&V consumption by the end of the simulation: Low scenario = 0 g/capita/day; reference scenario = 127 g/capita/day. |
|  |  |  | High (*combined capacity = 100* $\times$ *reference*) | Again, if prices start high, then cumulative purchases should remain below the reference mode. However, the trends may then switch if the retail price in Market B collapses. | ✓ | Per capita daily F&V consumption by the end of the simulation: High scenario = 99 g/capita/day; reference scenario = 127 g/capita/day. |
| D1. Market A customers | D1.1 | Total Loop sales | Low (*retail customers =* *0.1* $\times$ *reference)* | Retailers in Market A make up a small percentage of Loop buyers. Therefore, with retailer demands shrinking further, we expect a small to negligible impact upon total Loop sales. | ✓ | This scenario produces a 7.1% increase in the daily average Loop sales relative to the reference mode. Essentially, more Loop F&V supplies are routed towards the higher capacity distance traders. |
|  |  |  | High (*retail customers =* *100* $\times$ *reference)* | Increased retail demand should increase the flows of Loop supplies towards retailers in Market B. However, the capacity of retailers should remain below that of distance traders, potentially producing weaker sales than the reference mode. | ✓ |  |
|  | D1.2 | Retail F&V price in Market B | Low (*retail customers =* *0.1* $\times$ *reference)* | There is likely to be a negligible effect here, assuming that the local trader prices in Market A are also negligibly affected. | ✓ | Mean Market B retail price in extreme low scenario = 23.2 Rs/kg; Mean Market B retail price in reference scenario = 23.2 Rs/kg. |
|  |  |  | High (*retail customers =* *100* $\times$ *reference)* | If this scenario causes Market A’s capacity to retract, then Loop produce may spillover to Market B, and thus lower the retail price. | ✓ | Mean Market B retail price in extreme high scenario = 20.0 Rs/kg; Mean Market B retail price in reference scenario = 23.2 Rs/kg. |
|  | D1.3 | Loop revenues | Low (*retail customers =* *0.1* $\times$ *reference)* | Revenues are likely to be negligibly affected, combining D1.1 and D1.2. | ~ | This scenario produces a 4.8% increase in the daily average Loop revenue relative to the reference mode |
|  |  |  | High (*retail customers =* *100* $\times$ *reference)* | We may see increases if Loop takes advantage of the higher prices in Market A. However, these may be limited by the smaller market capacities and the potential overspill of supplies to Market B. | ✓ | This scenario produces a 52.0% increase in the daily average Loop revenue relative to the reference mode |
|  | D1.4 | Cumulative F&V purchases per retail customer in Market B | Low (*retail customers =* *0.1* $\times$ *reference)* | Assuming prices in Market B are negligibly affected, we should see a cumulative purchase trend that is similar to the reference mode. | ✓ | Per capita daily F&V consumption by the end of the simulation: Low scenario = 129 g/capita/day; reference scenario = 127 g/capita/day. |
|  |  |  | High (*retail customers =* *100* $\times$ *reference)* | Any spillover from Market A should lead to higher purchase rates in Market B. | ✓ | Per capita daily F&V consumption by the end of the simulation: High scenario = 140 g/capita/day; reference scenario = 127 g/capita/day. |
| D2. Market B customers | D2.1 | Total Loop sales | Low (*retail customers =* *0.1* $\times$ *reference)* | Again, similar to D1.1, retailers in Market B currently receive a small proportion of Loop supplies. Therefore, this scenario is likely to have a negligible impact on total Loop sales. | ✓ | Daily average Loop sales increase by 1.2% over the reference mode, as Market A (and the distance traders in Market A) become a more attractive supply route. |
|  |  |  | High (*retail customers =* *100* $\times$ *reference)* | Likely to observe a similar pattern to D1.1 (high), where Loop sales were limited by supplying the relatively low capacity retailer over the high capacity traders. | ~ | The model generates a slow decline in the number of farmers willing to supply Loop, owing to the higher wastage rates associated with opting for the retailers in Market B. |
|  | D2.2 | Retail F&V price in Market B | Low (*retail customers =* *0.1* $\times$ *reference)* | Low demand is likely to supress prices below the reference mode. | ✓ | Mean Market B retail price in extreme low scenario = 10.2 Rs/kg; Mean Market B retail price in reference scenario = 23.2 Rs/kg. |
|  |  |  | High (*retail customers =* *100* $\times$ *reference)* | In contrast to the above, a heightened number of retail customers is likely to increase retail prices. A peak and trough timeseries may result from temporary over-supply. | ✓ | Mean Market B retail price in extreme low scenario = 75.8 Rs/kg; Mean Market B retail price in reference scenario = 23.2 Rs/kg. |
|  | D2.3 | Loop revenues | Low (*retail customers =* *0.1* $\times$ *reference)* | As per D1.1 (low), this scenario is likely to have a negligible impact on Loop revenues. | ✓ |  |
|  |  |  | High (*retail customers =* *100* $\times$ *reference)* | As per D1.1 (high), we may see higher revenues driven by higher retail prices, although these may be limited by the total volumes sold. | ~ | Consistent with D1.1 (high), Loop revenues fall to 0 Rs/day by the end of the simulation, as non-Loop supply pathways are preferred. |
|  | D2.4 | Cumulative F&V purchases per retail customer in Market B | Low (*retail customers =* *0.1* $\times$ *reference)* | The absence of retail customers in Market B should produce cumulative F&V purchases that remain at 0 kg. | ✓ |  |
|  |  |  | High (*retail customers =* *100* $\times$ *reference)* | High prices (D2.2 – high) are likely to lead to lower purchase rates per retail customer. | ✓ | Per capita daily F&V consumption by the end of the simulation: High scenario = 20 g/capita/day; reference scenario = 127 g/capita/day. |

C.3 Extreme scenario output timeseries

| 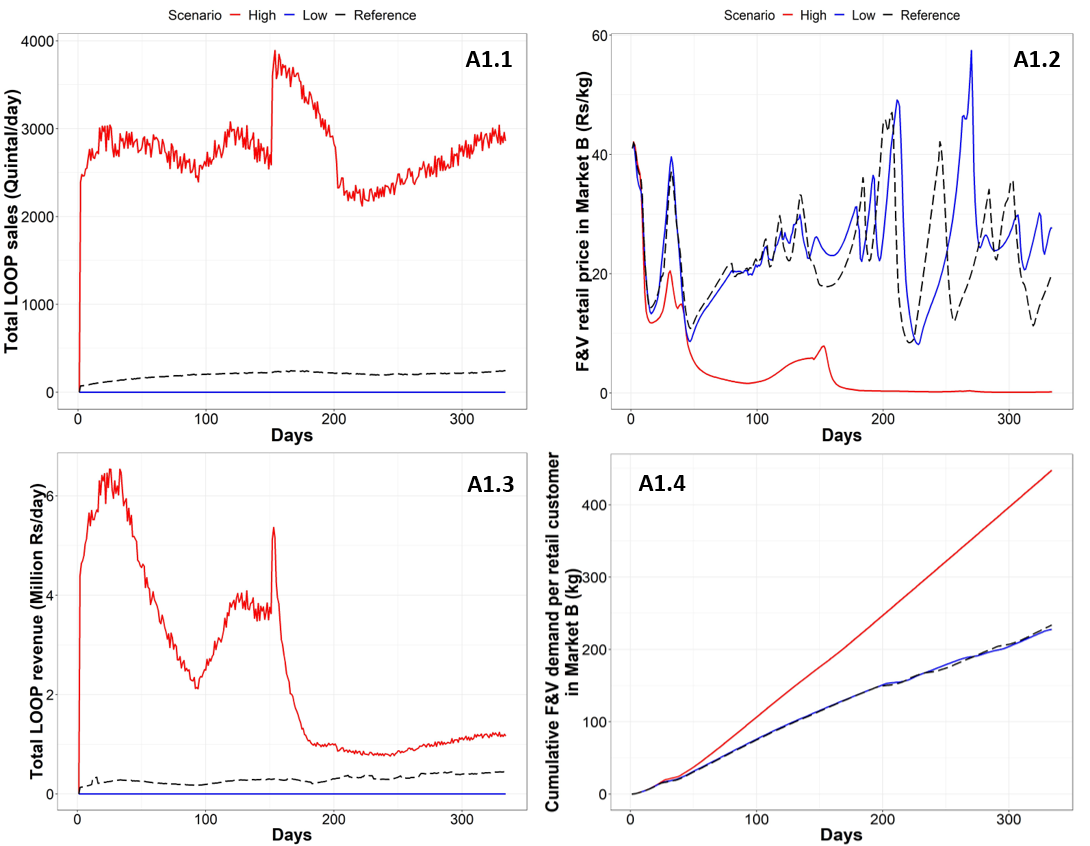 |
| --- |
| Figure S2.1: Output timeseries from the first extreme scenario experiment (A1). |
|  |
| 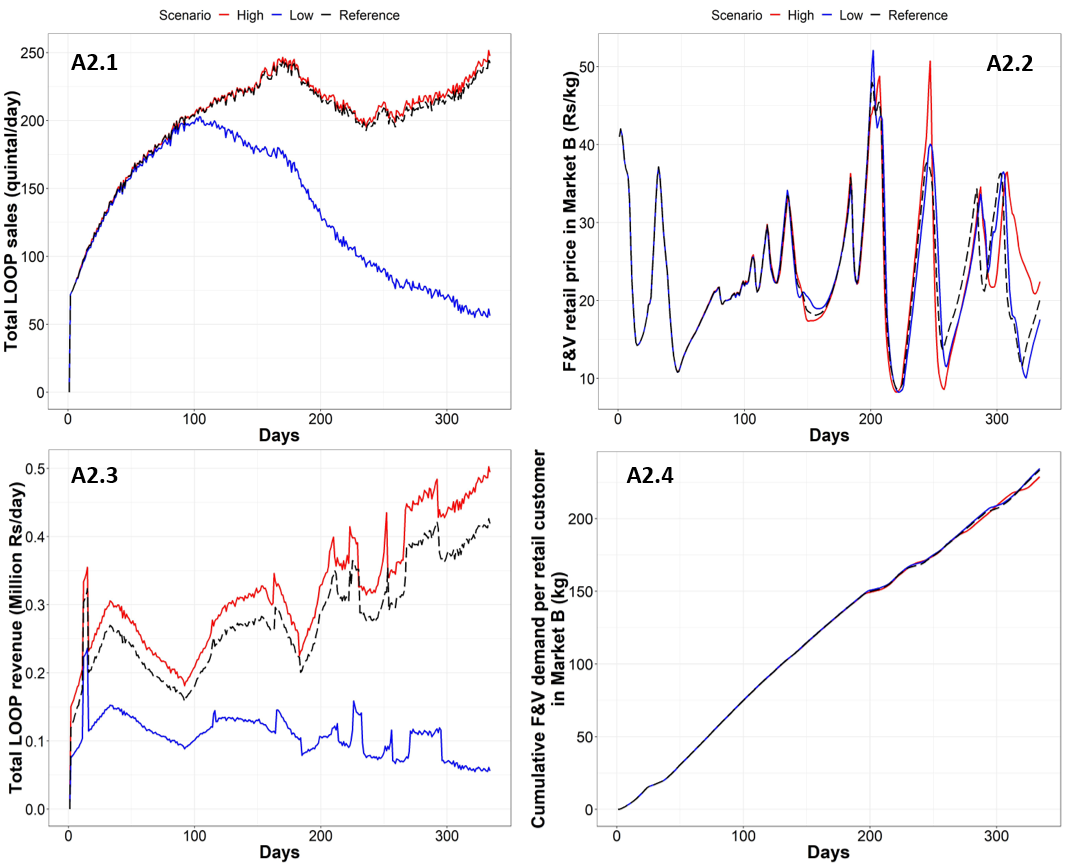 |
| Figure S2.2: Output timeseries from the second extreme scenario experiment (A2). |
| 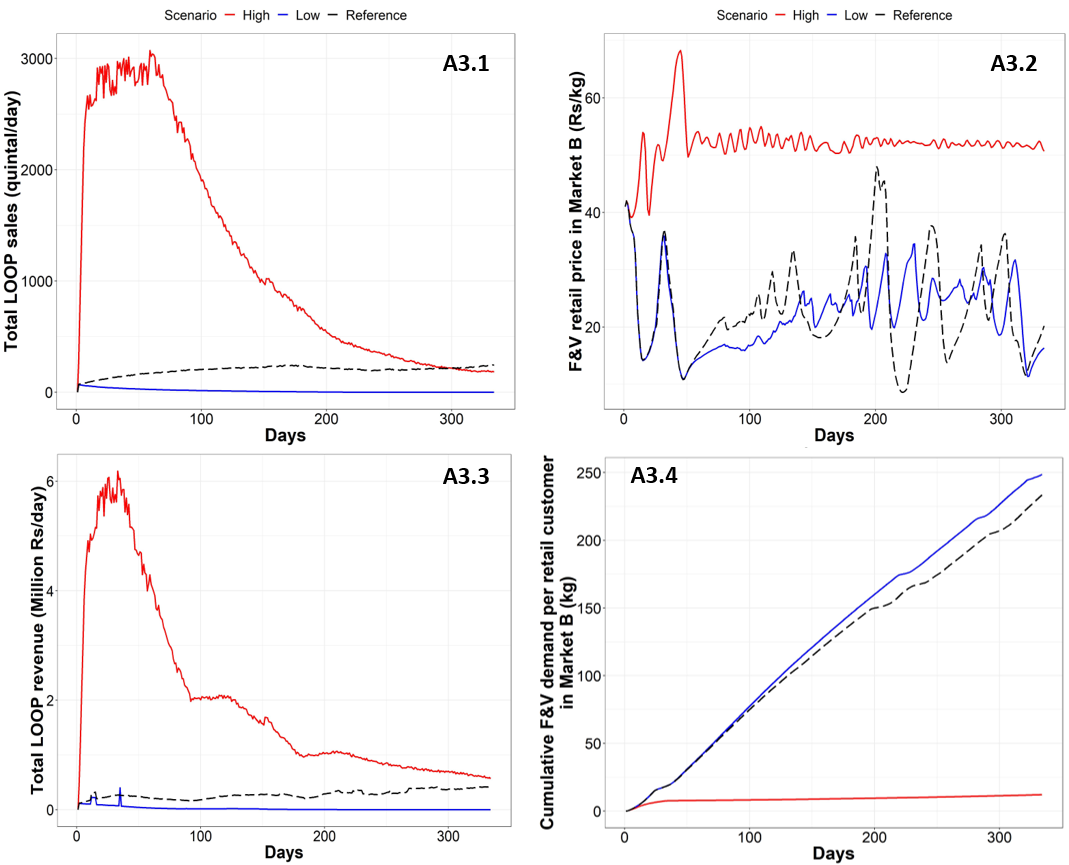 |
| Figure S2.3: Output timeseries from the third extreme scenario experiment (A3). |

| 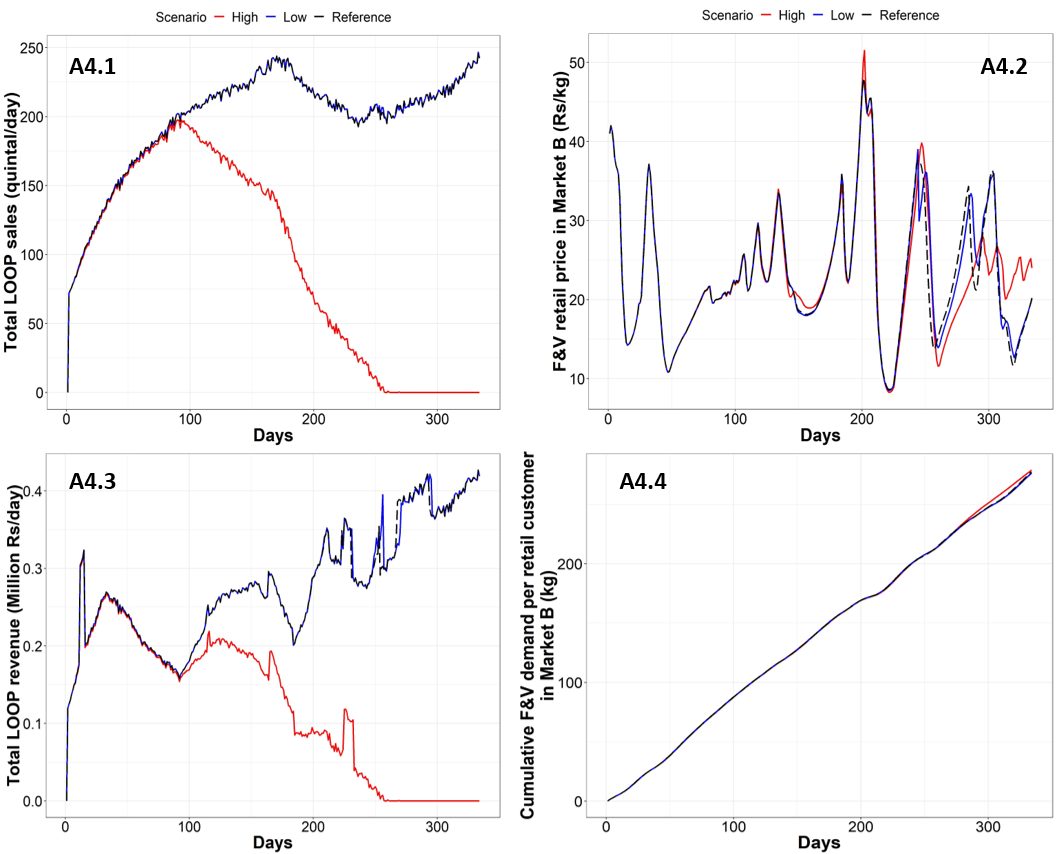 |
| --- |
| Figure S2.4: Output timeseries from the fourth extreme scenario experiment (A4). |
| 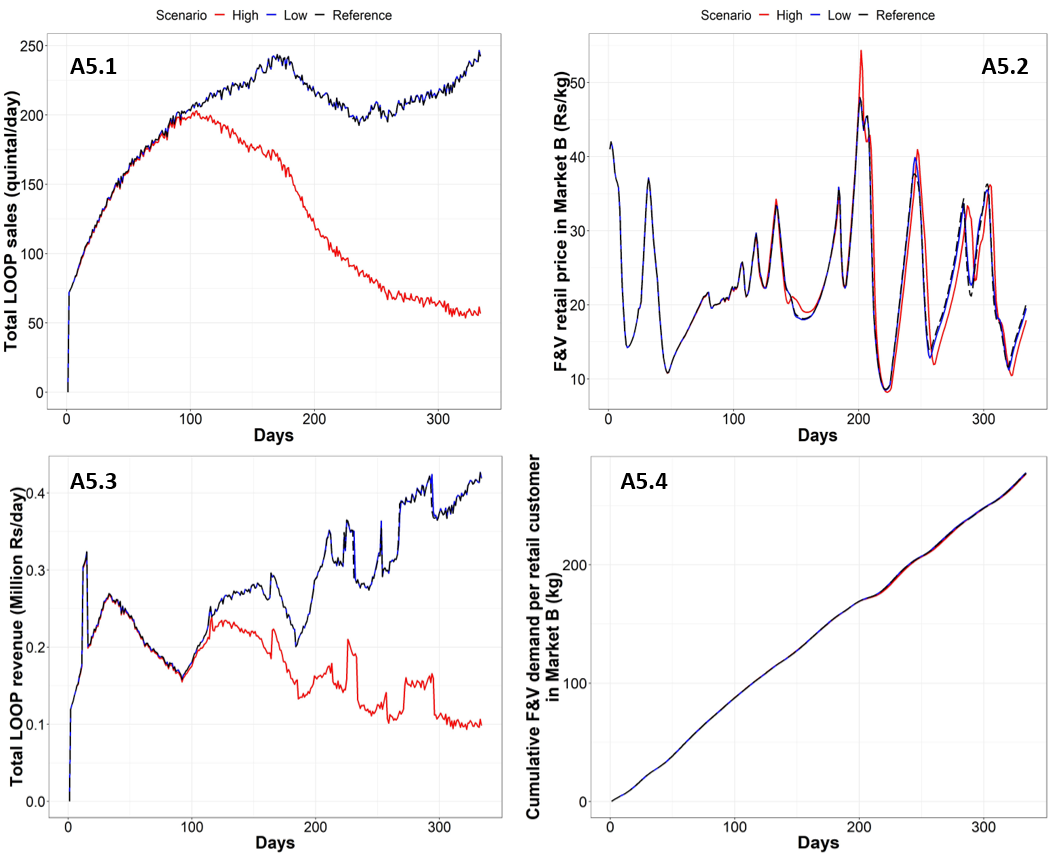 |
| Figure S2.5: Output timeseries from the fifth extreme scenario experiment (A5). |
| 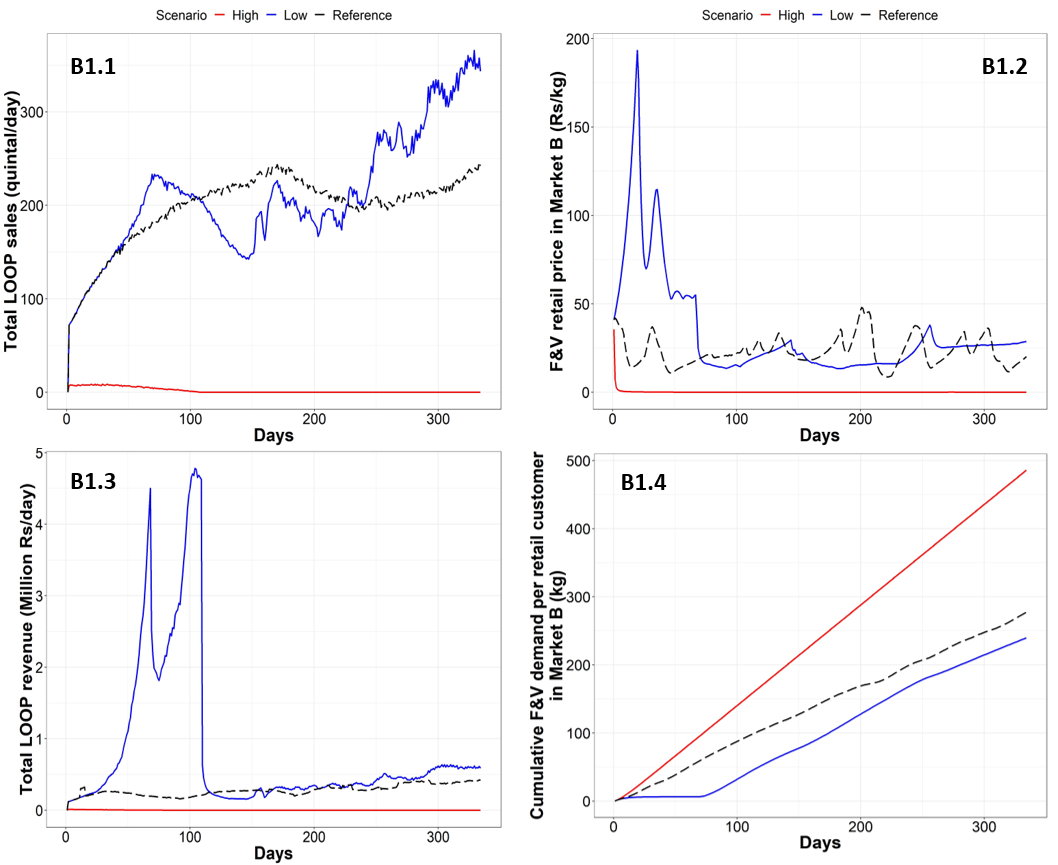 |
| Figure S3.1: Output timeseries from the sixth extreme scenario experiment (B1). |
| 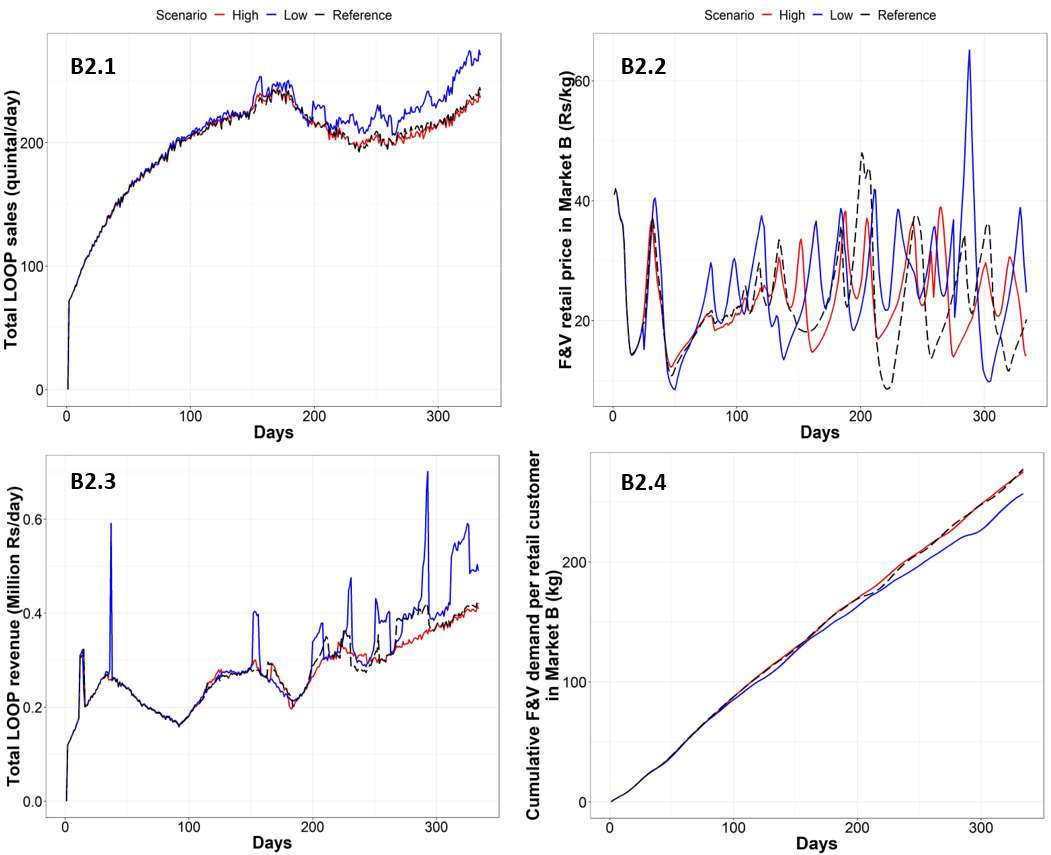 |
| Figure S3.2: Output timeseries from the sixth extreme scenario experiment (B2). |
| 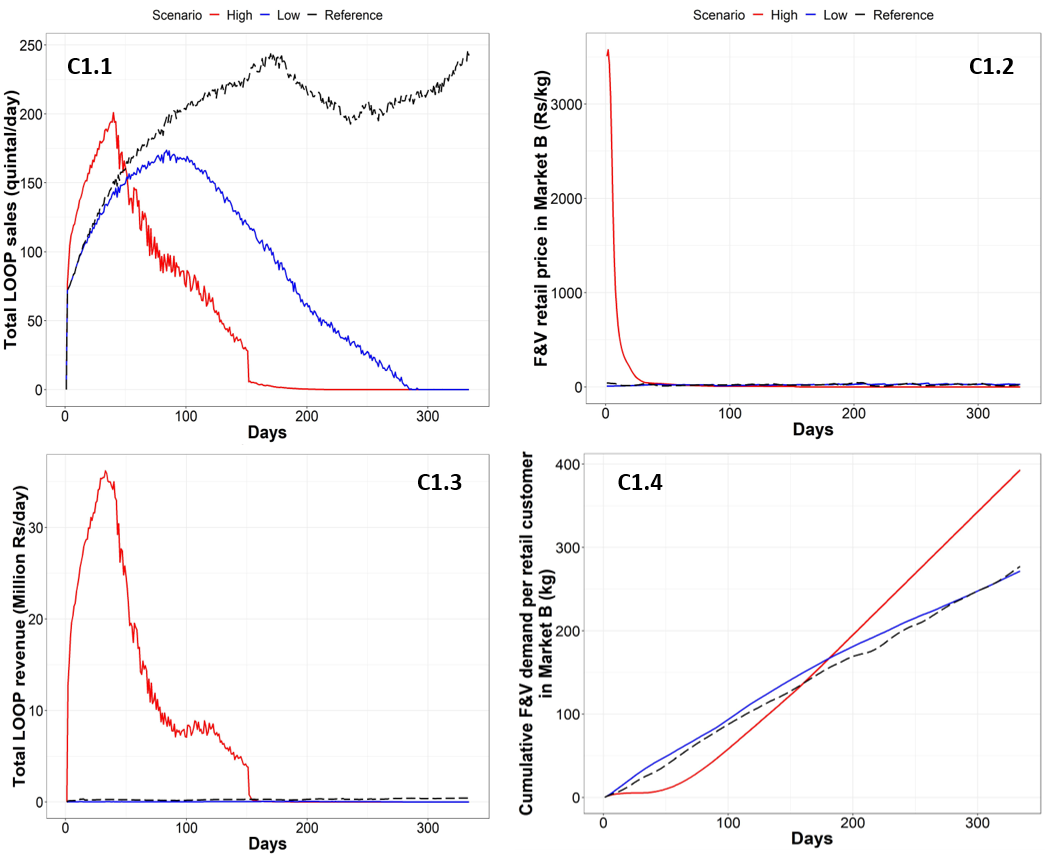 |
| Figure S4.1: Output timeseries from the seventh extreme scenario experiment (C1). |
| 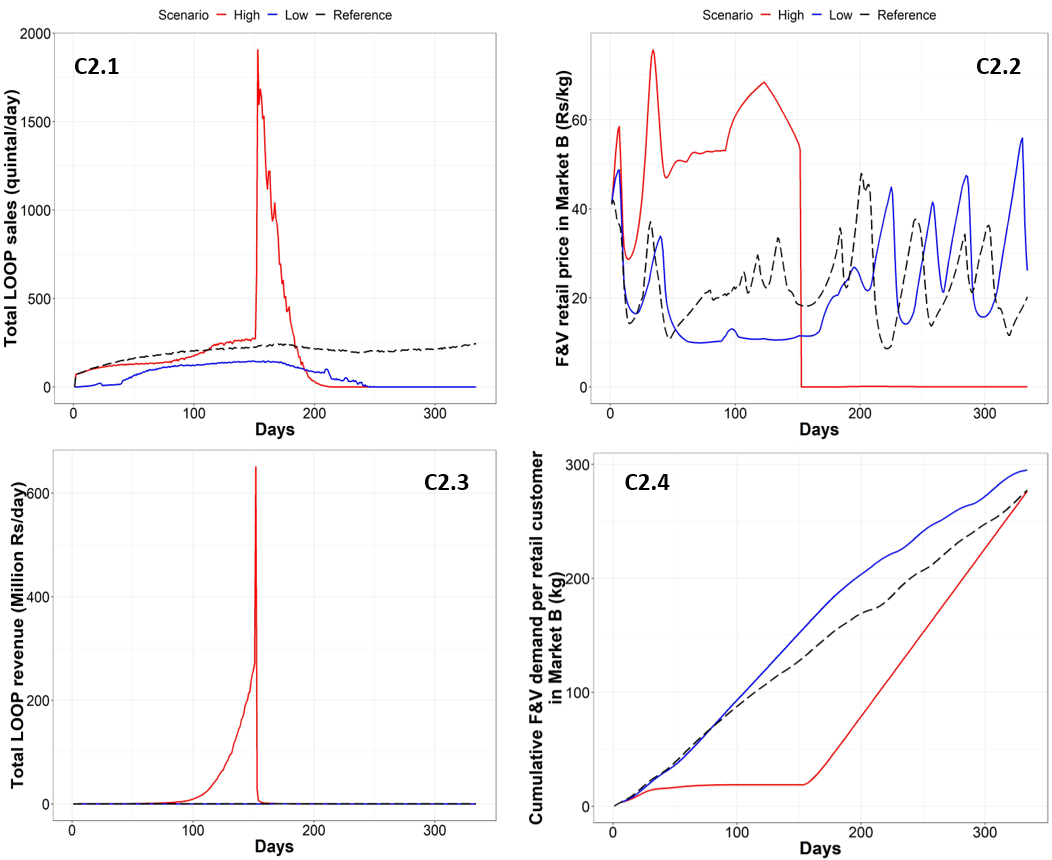 |
| Figure S4.2: Output timeseries from the eight extreme scenario experiment (C2). |
| 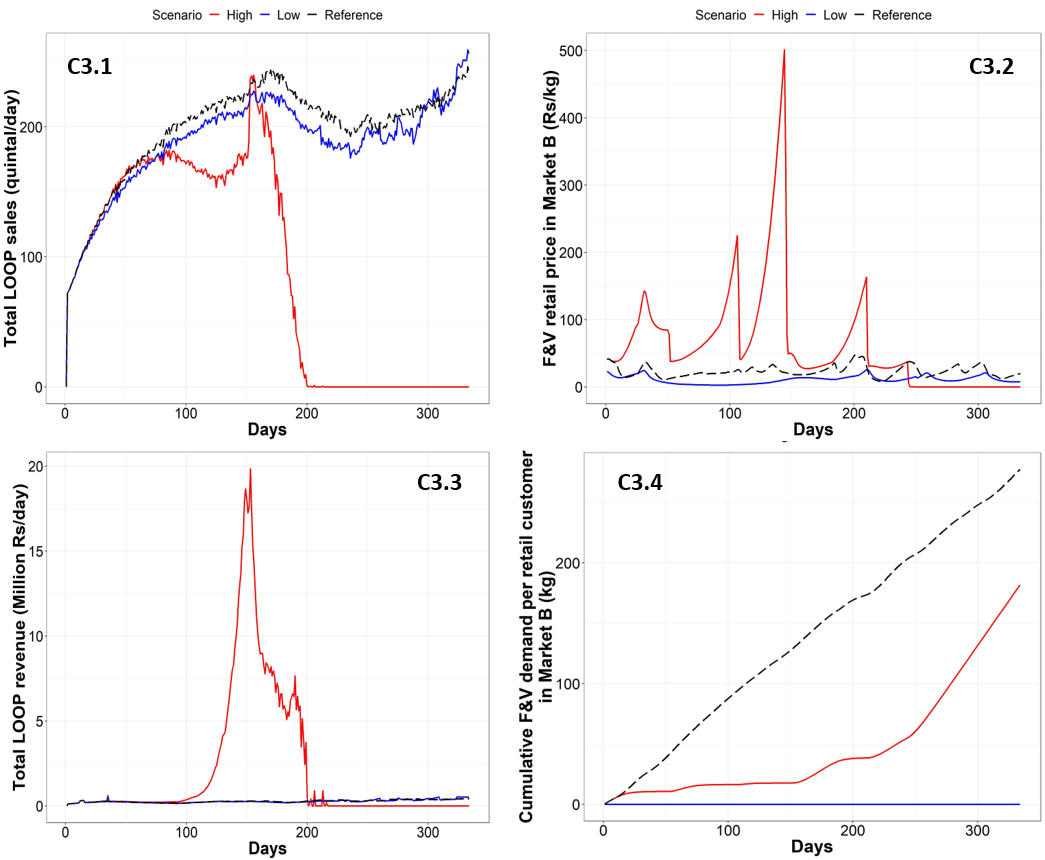 |
| Figure S4.3: Output timeseries from the ninth extreme scenario experiment (C3). |
| 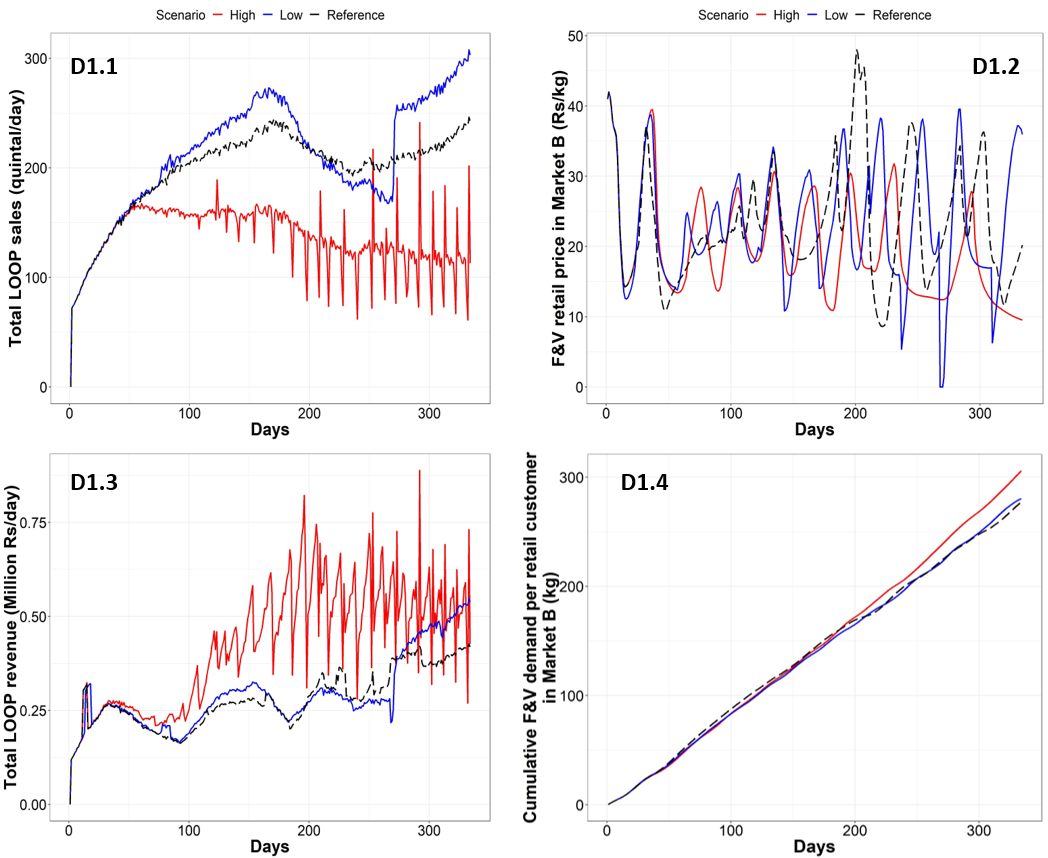 |
| Figure S5.1: Output timeseries from the tenth extreme scenario experiment (D1). |
| 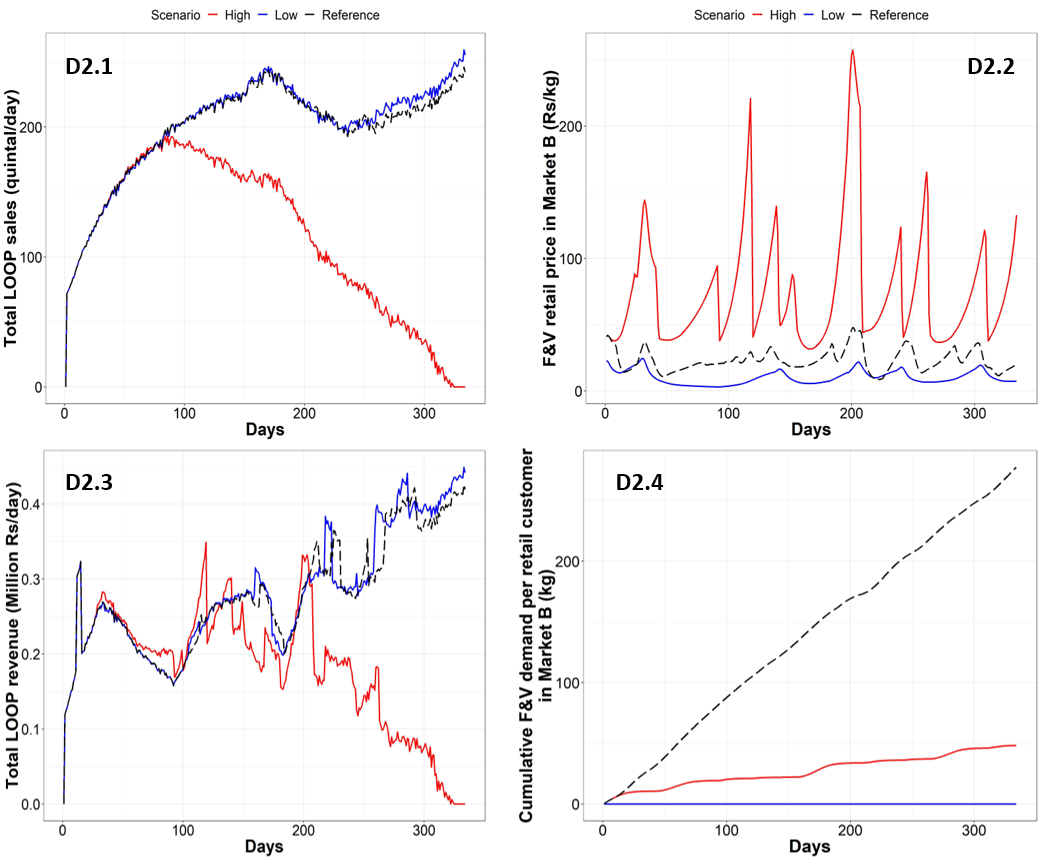 |
| Figure S5.2: Output timeseries from the eleventh extreme scenario experiment (D2). |

Supplementary Material D: Integration error test

The model presented in the main manuscript uses the ‘Euler’ integration method – recognised to be the most popular and simplest numerical technique employed to solve ordinary differential equations (Chichakly, 2010; Schwaninger and Groesser, 2016). However, the Euler method is not the only integration technique available in the modelling software STELLA (ISEES, 2018), and authors such as Seppelt and Richter (2005) and Chichakly (2010) have shown that outcomes deriving from the same model can be sensitive to the integration method employed. Given that the model has a number of time-sensitive variables (e.g. seasonal yields and land areas), we assess the impacts of changing the integration method from Euler to the higher-order ‘Runge-Kutta 4 (RK4)’ method on: (i) the absolute outcome values produced for ‘cumulative F&V purchases per retail customer in Market B’, ‘cumulative horticultural profits of Loop farmers’ and ‘average Loop ROI’ (Table S3), (ii) the general shape of the outcome space (Figure S6), and (iii) the scenario pathways to ‘win-win-win’ futures.

Given that the computational intensity of the RK4 method approximately triples the run-time of the model to 15 seconds per simulation, we simulate a smaller subset of 500 simulations for each of the two integration methods, under the scenario ranges detailed in Table 1 of the manuscript. To ensure that the same scenario trajectories are generated across both models (i.e. ‘Euler’ and ‘RK4’), each scenario type (e.g. Loop extension ‘Euler’, Loop extension ‘RK4’…) is given the same random number seed. As a consequence, any differences in outcomes are due to the differences in integration method employed.

| Table S3: Measures of the mean absolute percentage error (MAPE) and the coefficient of determination (R^2^) used to compare the three outcome dimensions produced by the ‘Euler’ and ‘RK4’ simulations (n = 500 each). MAPE is below 5% across all three outcomes and the coefficients of determination are all significant at the p < 0.001 significance level (***). | | | |  |
| --- | --- | --- | --- | --- |
|  | **Cumulative F&V purchases per retail customer in Market B** | **Cumulative horticultural profits of Loop farmers** | **Average Loop ROI** |  |
| **MAPE (%)** | 3.73 | 4.86 | 0.104 |  |
| **R^2^** | 0.89 *** | 0.94 *** | 0.97 *** |  |
| 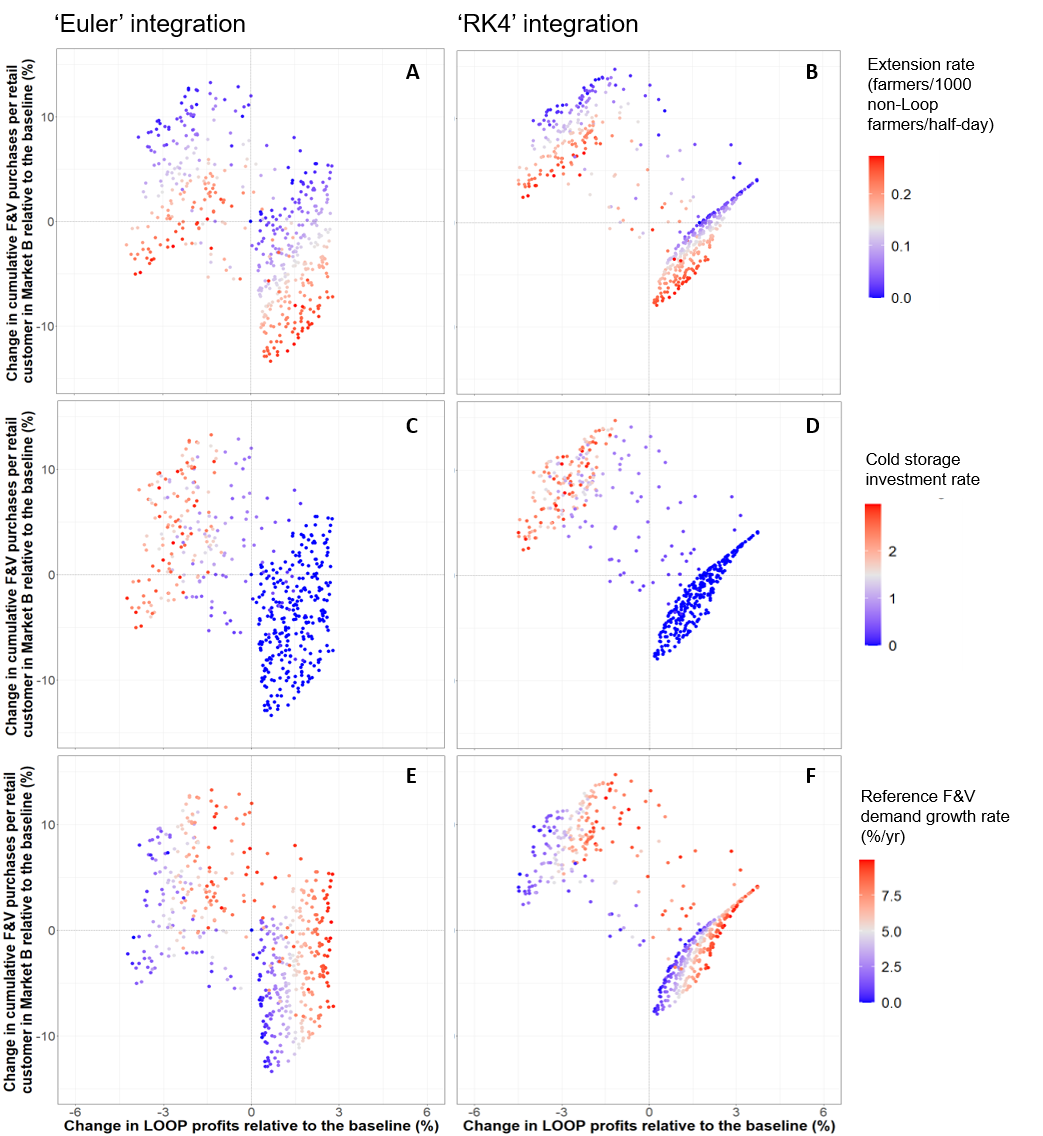 | | | | |
| Figure S6: Comparison of the outcome spaces produced by 500 iterations of the model using ‘Euler’ and ‘RK4’ integration. Outcomes from the ‘RK4’ simulations are more precisely clustered, reflecting the higher numerical precision of the ‘RK4’ integration method (Schwaninger and Groesser, 2016). However, the main features of the outcome space remain robust, including (i) the positive association between market-based cold storage and Market B retail purchases (subplot C-D), (ii) the negative association between Loop extension and F&V purchases from customers in Market B (subplot A-B), and (iii) the positive association between retail reference demand growth and Loop farmer profits (subplot E-F). | | | | |

| Table S4: Comparison of the ‘win-win-win’ scenario spaces emerging from the 500 ‘Euler’ and ‘RK4’ simulations. Overall, 7 ‘win-win-win’ outcomes were produced by the ‘Euler’ simulations, and 9 ‘win-win-win’ futures were produced by the ‘RK4’ simulations. Whilst the integration techniques introduce slight quantitative differences, the overarching scenarios towards ‘win-win-win’ futures remain robust (i.e. relatively low Loop extension, relatively low cold storage investment, and relatively high reference retail demand growth). Values in italics represent the ‘RK4’ dimensions. | | | | | |
| --- | --- | --- | --- | --- | --- |
| **Scenario** | | **‘Win-win-win’ space** | | | |
| Name | Trajectory range (units) | Mean | Std.dev | Lower limit | Upper limit |
| Loop extension | 0 🡪 0.274  (farmers/1000 non- Loop farmers/half-day) | 0.126  *0.087* | 0.048  *0.023* | 0.079  *0.056* | 0.195  *0.132* |
| Loop transport subsidy | 0 🡪 100  (%) | 24.2  *32.3* | 17.8  *23.6* | 4.21  *14.2* | 57.5  *84.4* |
| Cold storage investment rate | 0 🡪 3  (Unitless) | 0.367  *0* | 0.292  *0* | 0  *0* | 0.667  *0* |
| Reference retail demand growth rate | 0 🡪 10  (%/year) | 8.91  *8.00* | 0.663  *1.59* | 8.07  *5.66* | 9.80  *9.97* |

Supplementary Material E: Monte Carlo sensitivity analysis

E.1 One-at-a-time output table

| Table S4: Outputs of the first stage of the Monte Carlo sensitivity analysis detailed in Section 2.3. The coloured cells in the second column detail the percentage of daily Loop farmer values that are behaviour-giving (i.e. fall within the 95% error bounds of the observed data – see Figures S7 onwards); the equivalent cells in the third column detail the percentage of modelled daily Loop sales that are behaviour-giving (i.e. fall within the 95% error bounds of the observed data – see Figures S8 onwards). ‘ϵ’ – error range; ‘SD’ – standard deviation. Variables highlighted in yellow correspond to ‘yellow sensitivity’ variables – as one or more of their error ranges produces behaviour-giving percentages between 90-94.9%. Likewise, variables highlighted in red correspond to ‘red sensitivity’ variables – whereby one or more of their error ranges produce behaviour-giving percentages less than 90%. | | | | | | | | | | |
| --- | --- | --- | --- | --- | --- | --- | --- | --- | --- | --- |
| **Module** | **Sensitivity variable** |  | **Total Loop farmers in Koilwar block** | | | | **F&V sales in Market A (kg/day)** | | | |
|  |  | **Variable ID** | **SD of final value** | **± 5% ϵ** | **± 10% ϵ** | **± 25% ϵ** | **SD of final value** | **± 5% ϵ** | **± 10% ϵ** | **± 25% ϵ** |
| **Production & aggregation** | Non-Loop Market A prop. | B1 | 16.8 | 100 | 100 | 100 | 2430 | 90.0 | 84.6 | 65.1 |
|  | Non-Loop Market B prop. | B2 | 5.56 | 100 | 100 | 100 | 1080 | 96.6 | 88.7 | 80.7 |
|  | Non-Loop Market A random | B3 | 16.8 | 100 | 100 | 100 | 2440 | 90.0 | 84.6 | 65.2 |
|  | Non-Loop Market B random | B4 | 5.56 | 100 | 100 | 100 | 1080 | 96.6 | 88.7 | 80.9 |
|  | Market profit weight | B5 | 0.005 | 100 | 100 | 100 | 34.7 | 100 | 100 | 100 |
|  | Market ‘guarantee sales’ weight | B6 | 0.002 | 100 | 100 | 100 | 2.26 | 100 | 100 | 100 |
| **Market A dynamics** | Price perception time | C1 | 0.97 | 100 | 100 | 100 | 500 | 99.0 | 92.5 | 93.0 |
|  | Trust perception time | C2 | 0.40 | 100 | 100 | 100 | 180 | 99.9 | 99.8 | 99.6 |
|  | Distance trader price sensitivity to inventory coverage | C3 | 0.42 | 100 | 100 | 100 | 350 | 99.8 | 98.2 | 97.1 |
|  | Distance trader price sensitivity to costs | C4 | 0.56 | 100 | 100 | 100 | 270 | 99.9 | 99.9 | 97.4 |
|  | Trader cost perception time | C5 | 0.04 | 100 | 100 | 100 | 82.6 | 100 | 99.9 | 99.9 |
|  | Distance trader profit perception time | C6 | 0 | 100 | 100 | 100 | 0 | 100 | 100 | 100 |
|  | Local trader price sensitivity to inventory coverage | C7 | 1.67 | 100 | 100 | 100 | 420 | 99.0 | 95.8 | 90.6 |
|  | Local trader price sensitivity to costs | C8 | 7.99 | 100 | 100 | 100 | 320 | 97.4 | 99.1 | 99.8 |
|  | Local trader profit perception time | C9 | 1.76 | 100 | 100 | 100 | 550 | 99.4 | 98.9 | 91.9 |
|  | Retailer price sensitivity to inventory coverage | C10 | 3.25 | 100 | 100 | 100 | 630 | 98.7 | 92.3 | 88.1 |
|  | Retailer price sensitivity to costs | C11 | 2.50 | 100 | 100 | 100 | 520 | 98.0 | 95.6 | 88.6 |
|  | Retailer profit perception time | C12 | 0.31 | 100 | 100 | 100 | 350 | 100 | 100 | 94.8 |
| **Market B dynamics** | Price perception time | D1 | 0.40 | 100 | 100 | 100 | 320 | 99.7 | 98.8 | 98.6 |
|  | Trust perception time | D2 | 0.09 | 100 | 100 | 100 | 220 | 99.9 | 99.8 | 99.5 |
|  | Local trader price sensitivity to inventory coverage | D3 | 0.47 | 100 | 100 | 100 | 430 | 99.5 | 99.5 | 97.2 |
|  | Local trader price sensitivity to costs | D4 | 0.09 | 100 | 100 | 100 | 180 | 99.3 | 100 | 99.9 |
|  | Trader cost perception time | D5 | 0.03 | 100 | 100 | 100 | 190 | 94.3 | 97.3 | 97.0 |
|  | Local trader profit perception time | D6 | 0.10 | 100 | 100 | 100 | 230 | 99.8 | 99.5 | 99.7 |
|  | Retailer price sensitivity to inventory coverage | D7 | 1.35 | 100 | 100 | 100 | 590 | 99.2 | 97.8 | 90.0 |
|  | Retailer price sensitivity to costs | D8 | 0.11 | 100 | 100 | 100 | 330 | 99.5 | 100 | 99.9 |
|  | Retailer profit perception time | D9 | 0.09 | 100 | 100 | 100 | 170 | 99.9 | 99.2 | 99.9 |
| **Retail demand** | Market A consumption demand adjustment delay | E1 | 0.69 | 100 | 100 | 100 | 330 | 99.2 | 98.5 | 97.9 |
|  | Market A maximum retail consumption | E2 | 0 | 100 | 100 | 100 | 0 | 100 | 100 | 100 |
|  | Market A retail reference price | E3 | 8.57 | 100 | 100 | 100 | 690 | 91.1 | 92.8 | 90.3 |
|  | Market B consumption demand adjustment delay | E4 | 0.16 | 100 | 100 | 100 | 260 | 99.9 | 98.9 | 97.0 |
|  | Market B maximum retail consumption | E5 | 0 | 100 | 100 | 100 | 0 | 100 | 100 | 100 |
|  | Market B retail reference price | E6 | 2.58 | 100 | 100 | 100 | 980 | 95.8 | 87.2 | 82.9 |
| **Loop benefits and costs** | Loop yield investment rate | F1 | 0.16 | 100 | 100 | 100 | 370 | 100 | 100 | 99.7 |
|  | Conversion rate between yield and investment | F2 | 0.18 | 100 | 100 | 100 | 430 | 99.1 | 100 | 100 |
|  | Loop land investment rate | F3 | 0 | 100 | 100 | 100 | 0 | 100 | 100 | 100 |
|  | Loop utility perception time | F4 | 180 | 99.8 | 91.7 | 58.8 | 3900 | 88.5 | 83.3 | 55.9 |
|  | Loop profit utility weight | F5 | 0.55 | 100 | 100 | 100 | 150 | 99.6 | 100 | 99.9 |
|  | Loop sales utility weight | F6 | 6.45 | 100 | 100 | 100 | 140 | 99.6 | 100 | 100 |
|  | Loop profit weight in trust | F7 | 7.54 | 100 | 100 | 100 | 300 | 99.4 | 99.8 | 99.5 |
|  | Loop sales weight in trust | F8 | 0.73 | 100 | 100 | 100 | 160 | 100 | 99.4 | 99.9 |
| **Non-Loop benefits and costs** | Non-Loop yield investment rate morning | G1 | 0.44 | 100 | 100 | 100 | 120 | 99.9 | 100 | 99.8 |
|  | Non-Loop yield investment rate afternoon | G2 | 0.02 | 100 | 100 | 100 | 80 | 99.9 | 100 | 100 |
|  | Non-Loop yield investment conversion morning | G3 | 0.06 | 100 | 100 | 100 | 140 | 100 | 100 | 99.6 |
|  | Non-Loop yield investment conversion afternoon | G4 | 0.02 | 100 | 100 | 100 | 80 | 100 | 100 | 99.9 |
|  | Non-Loop land investment rate morning | G5 | 0 | 100 | 100 | 100 | 0 | 100 | 100 | 100 |
|  | Non-Loop land investment rate afternoon | G6 | 0 | 100 | 100 | 100 | 0 | 100 | 100 | 100 |

E.2 One-at-a-time output timeseries

| 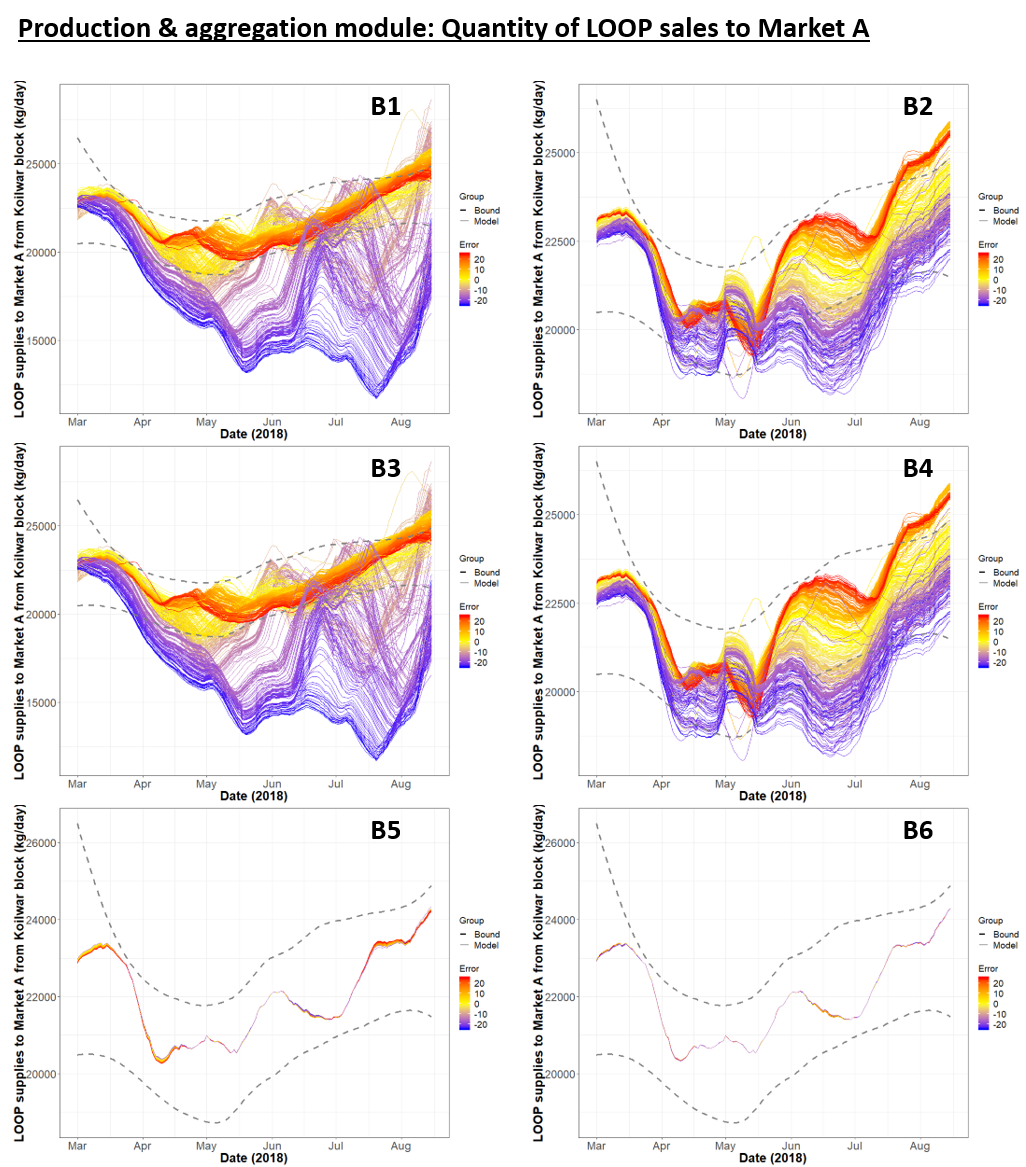 |
| --- |
| Figure S7: Sensitivity outputs from the ‘Production and aggregation’ (B1-B6) module. The dashed black lines equal the upper and lower 95% error bounds of the LOESS regression model for the daily Loop sales in Market A (March – August 2018).   \| 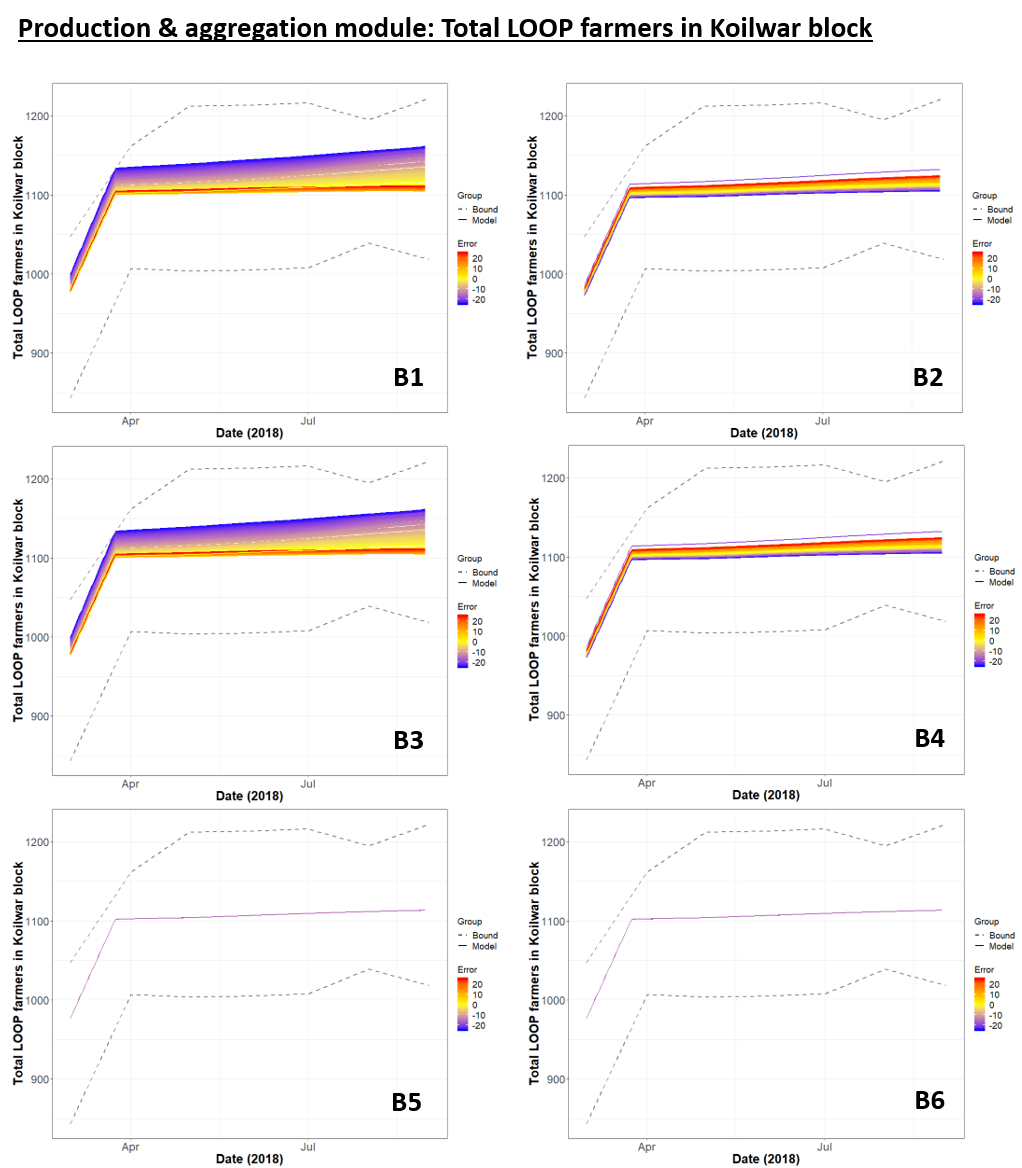 \| \| --- \| \| Figure S8: Sensitivity outputs from the ‘Production and aggregation’ (B1-B6) module. The dashed black lines equal the upper and lower 95% error bounds of the LOESS regression model for the total number of farmers in Loop (March – August 2018). \| |

| 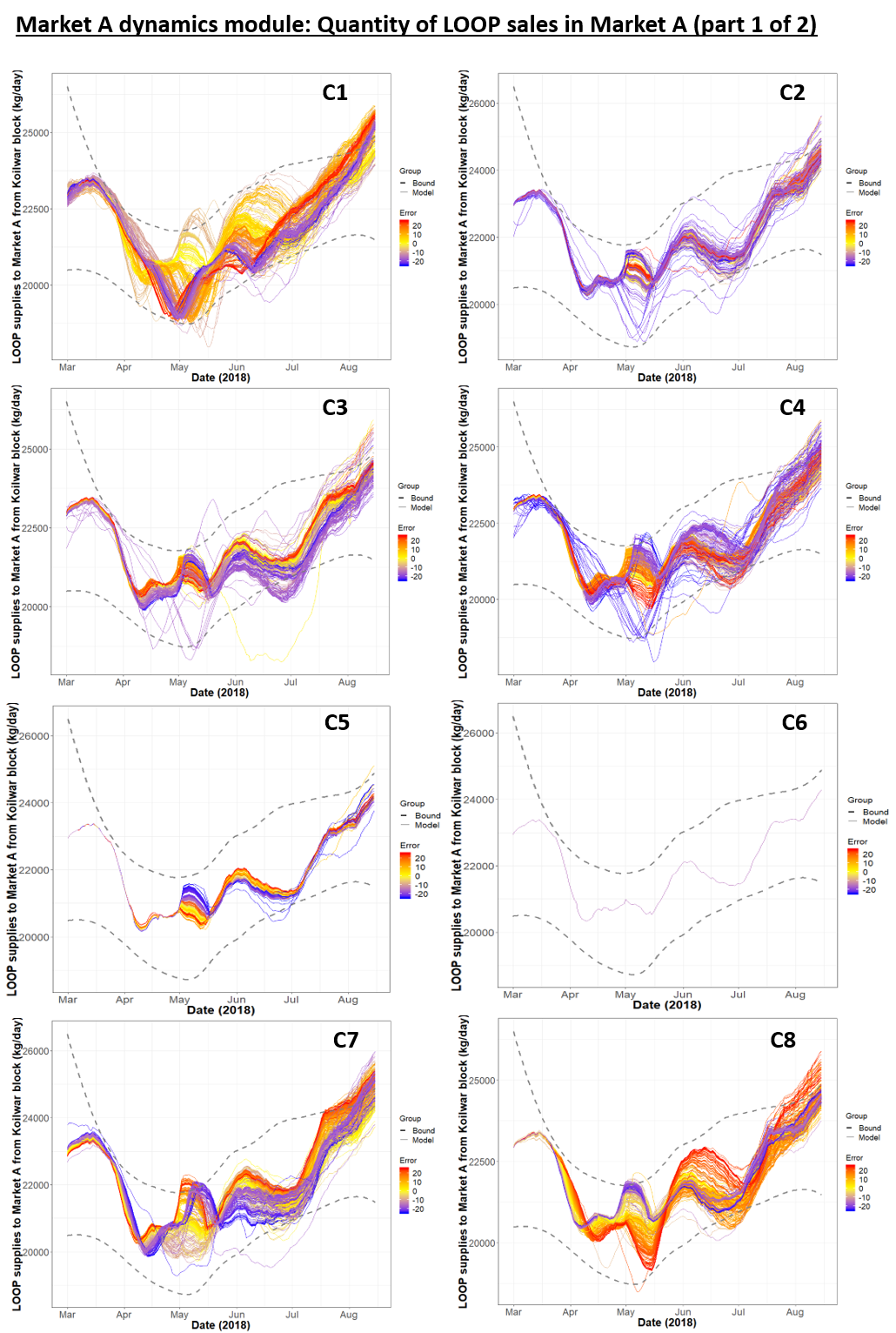 |
| --- |
| Figure S9a: First part of the sensitivity outputs from the ‘Market A dynamics’ module (C1-C8). The dashed black lines equal the upper and lower 95% error bounds of the LOESS regression model for the daily Loop sales in Market A (March – August 2018). |
| 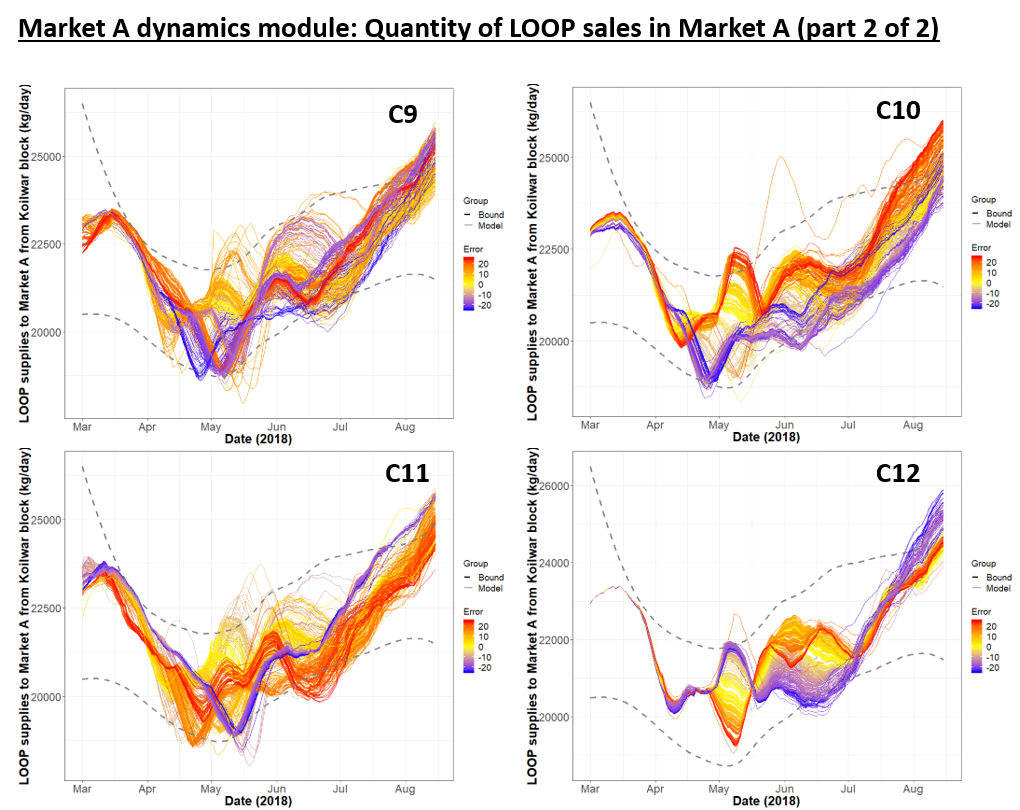 |
| Figure S9b: Second part of sensitivity outputs from the ‘Market A dynamics’ module (C9-C12). The dashed black lines equal the upper and lower 95% error bounds of the LOESS regression model for the daily Loop sales in Market A (March – August 2018). |
| 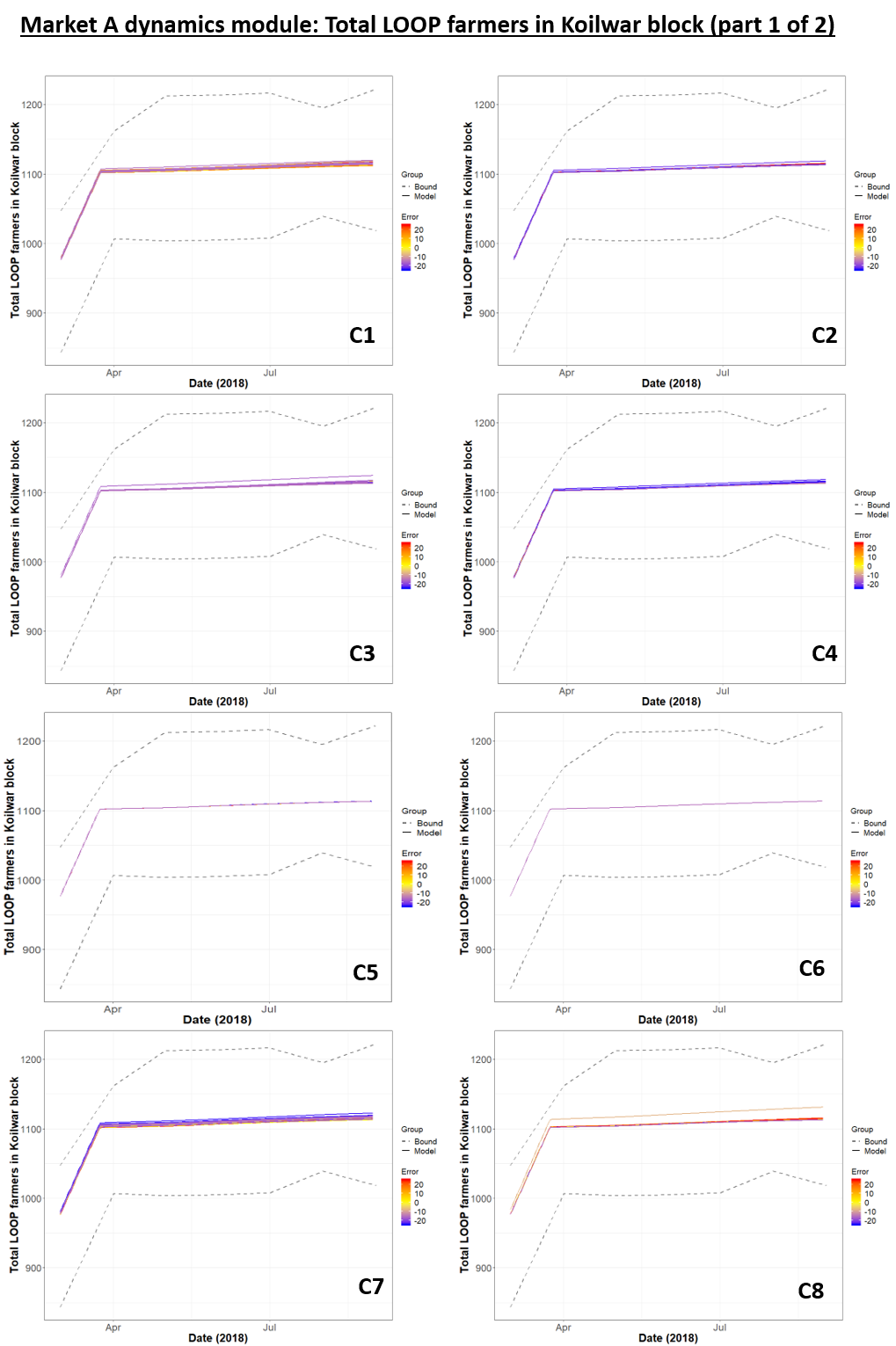 |
| Figure S10a: First part of the sensitivity outputs from the ‘Market A Dynamics’ module (C1-C8). As per figure S7, the dashed black lines equal the upper and lower 95% error bounds of the LOESS regression model for the total number of farmers in Loop (March – August 2018). |
| 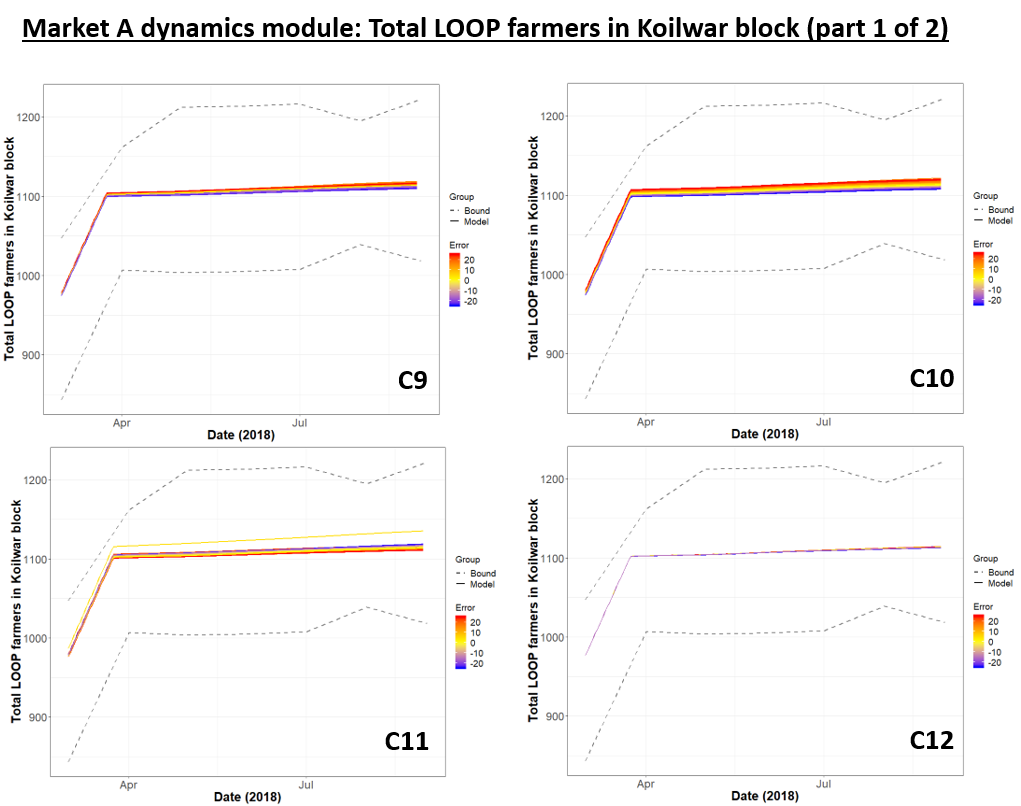 |
| Figure S10b: Second part of the sensitivity outputs from the ‘Market A Dynamics’ module (C1-C8). As per figure S9a, the dashed black lines equal the upper and lower 95% error bounds of the LOESS regression model for the total number of farmers in Loop (March – August 2018). |

| 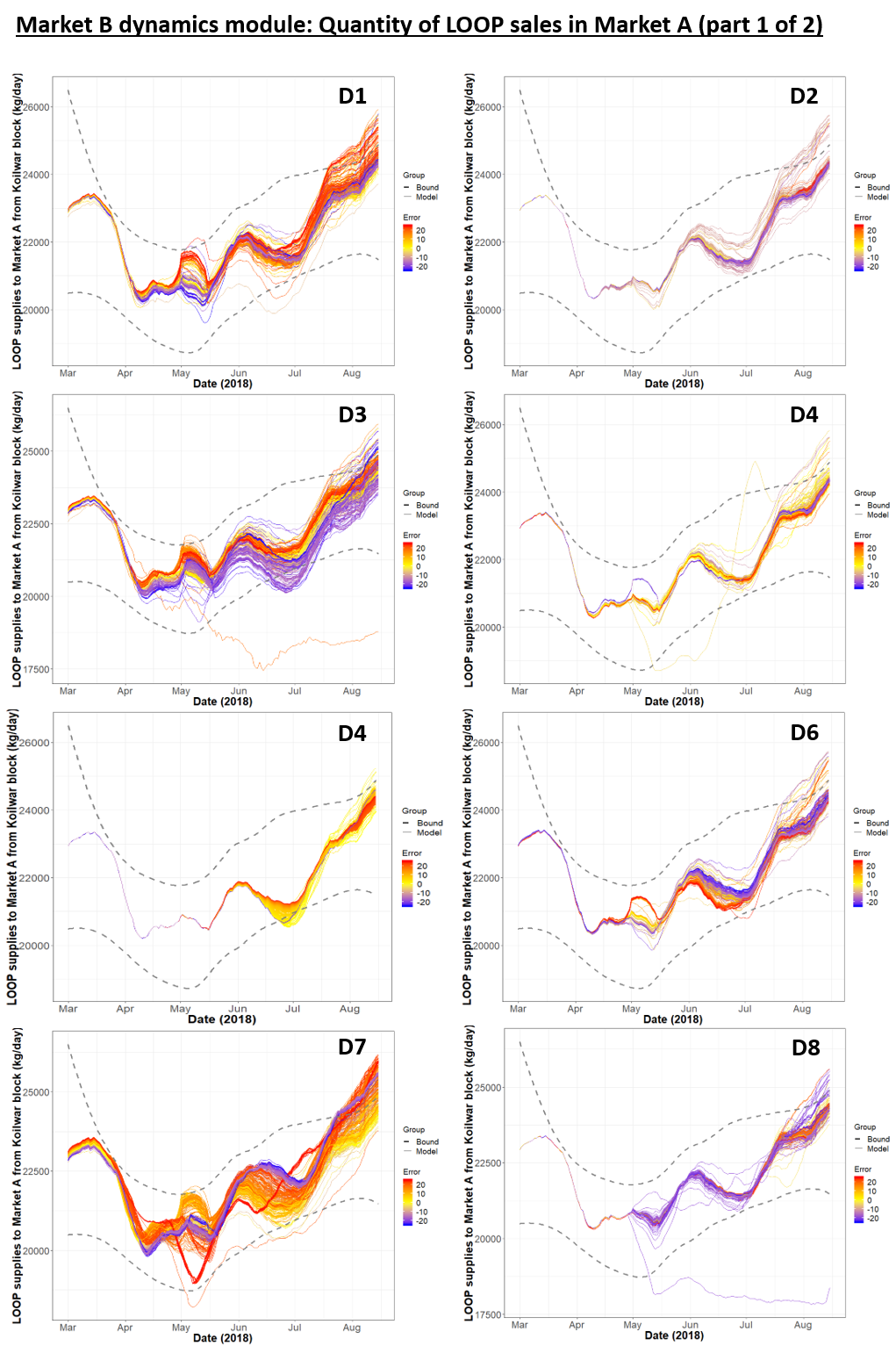 |
| --- |
| Figure S11a: First part of the sensitivity outputs from the ‘Market B Dynamics’ module (D1-D8) – visualised as the daily Loop sales in Market A. The black dashed lines have the same meaning as in figure S8a. |

| 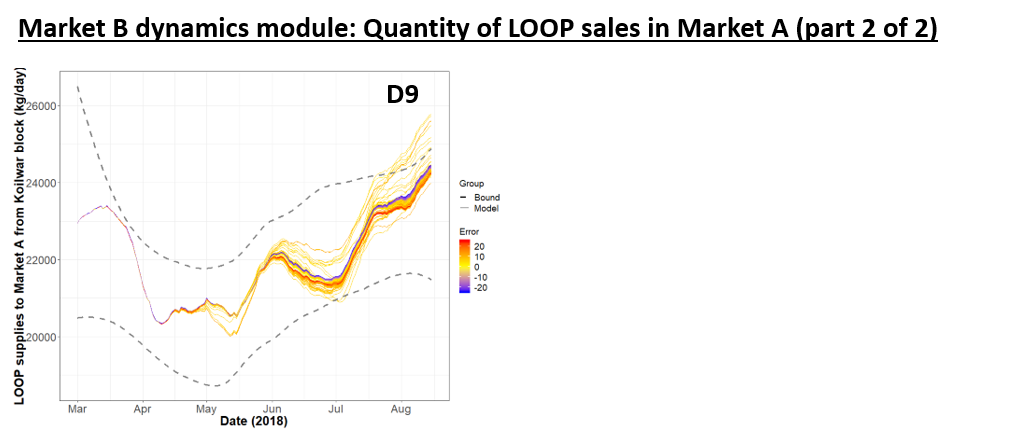 |
| --- |
| Figure S11b: Second part of the sensitivity outputs from the ‘Market B Dynamics’ module (D9) – visualised as the daily Loop sales in Market A. The black dashed lines have the same meaning as in figure S8a. |

| 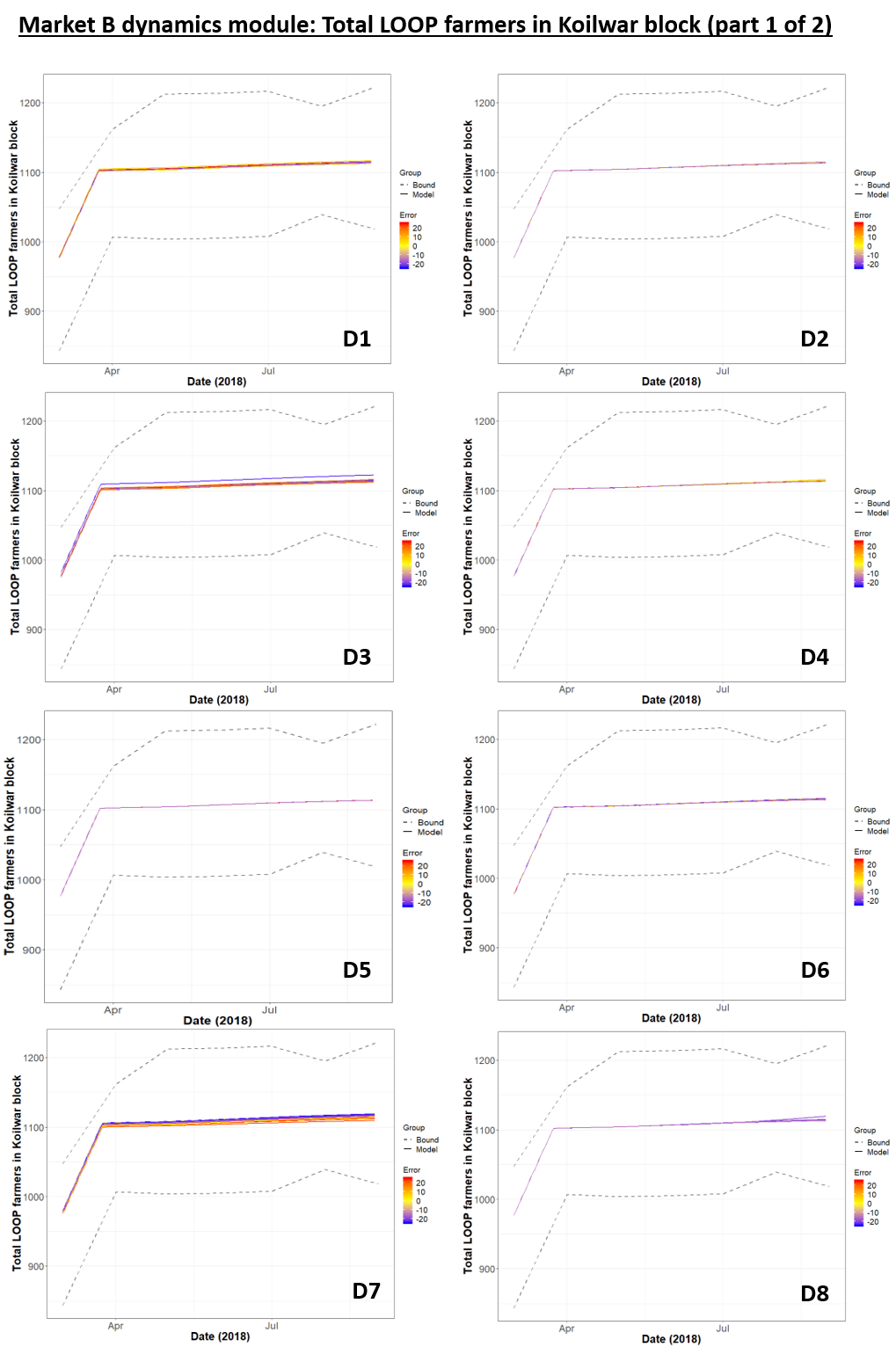 |
| --- |
| Figure S12a: First part of the sensitivity outputs from the ‘Market B Dynamics’ module (D1-D8) – visualised as the total number of farmers in Loop. The black dashed lines have the same meaning as in figure S9a. |
| 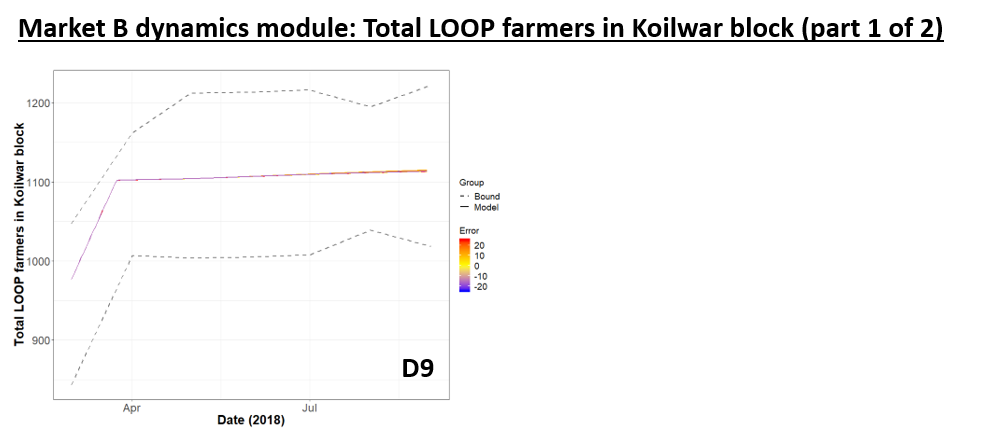 |
| Figure S12b: Second part of the sensitivity outputs from the ‘Market B Dynamics’ module (D9) – visualised as the total number of farmers in Loop. The black dashed lines have the same meaning as in figure S9a. |

| 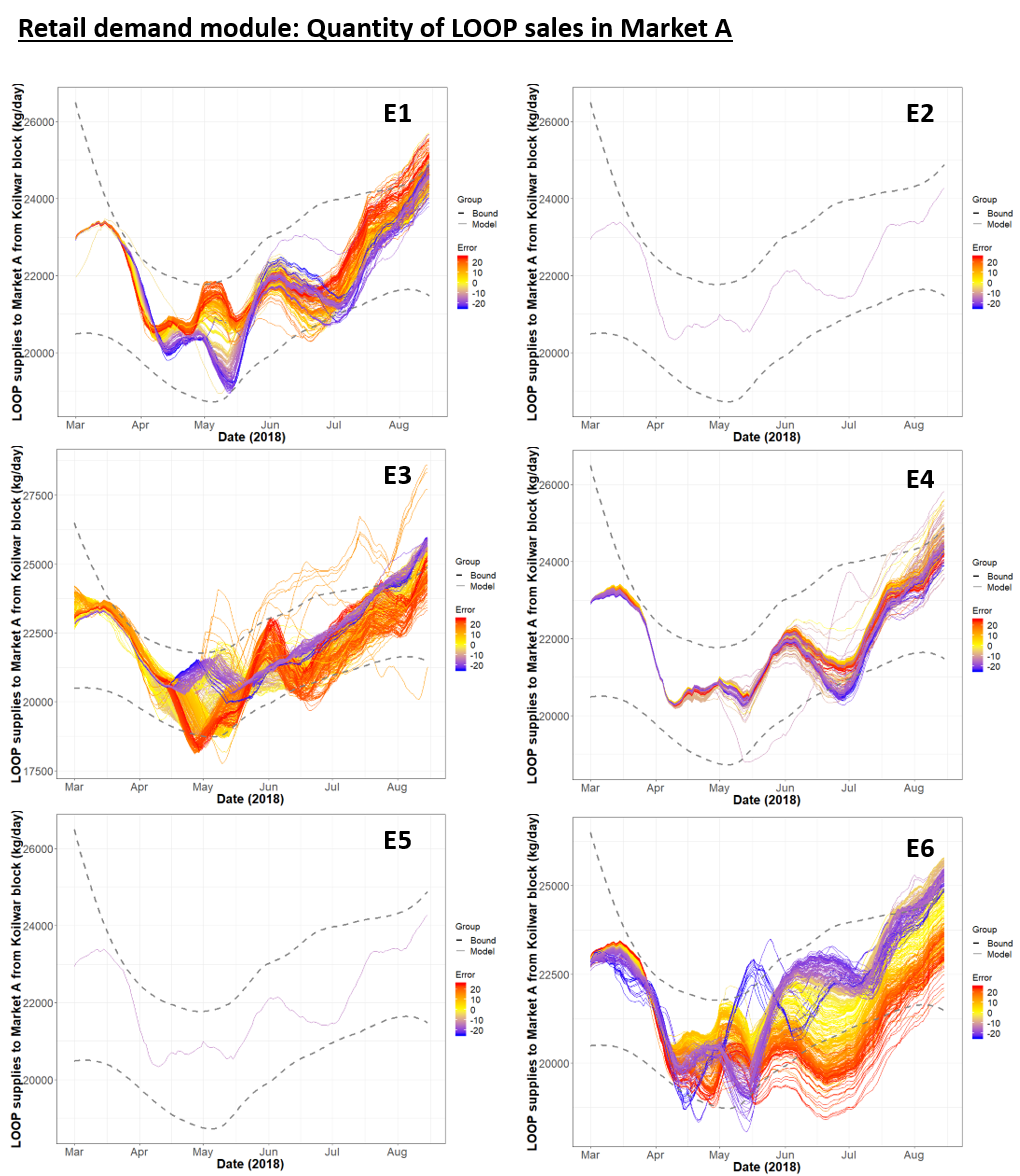 |
| --- |
| Figure S13: Sensitivity outputs from the ‘Retail demand’ module (E1-E6) – visualised as the daily Loop sales in Market A. The black dashed lines have the same meaning as in figure S8a. |

| 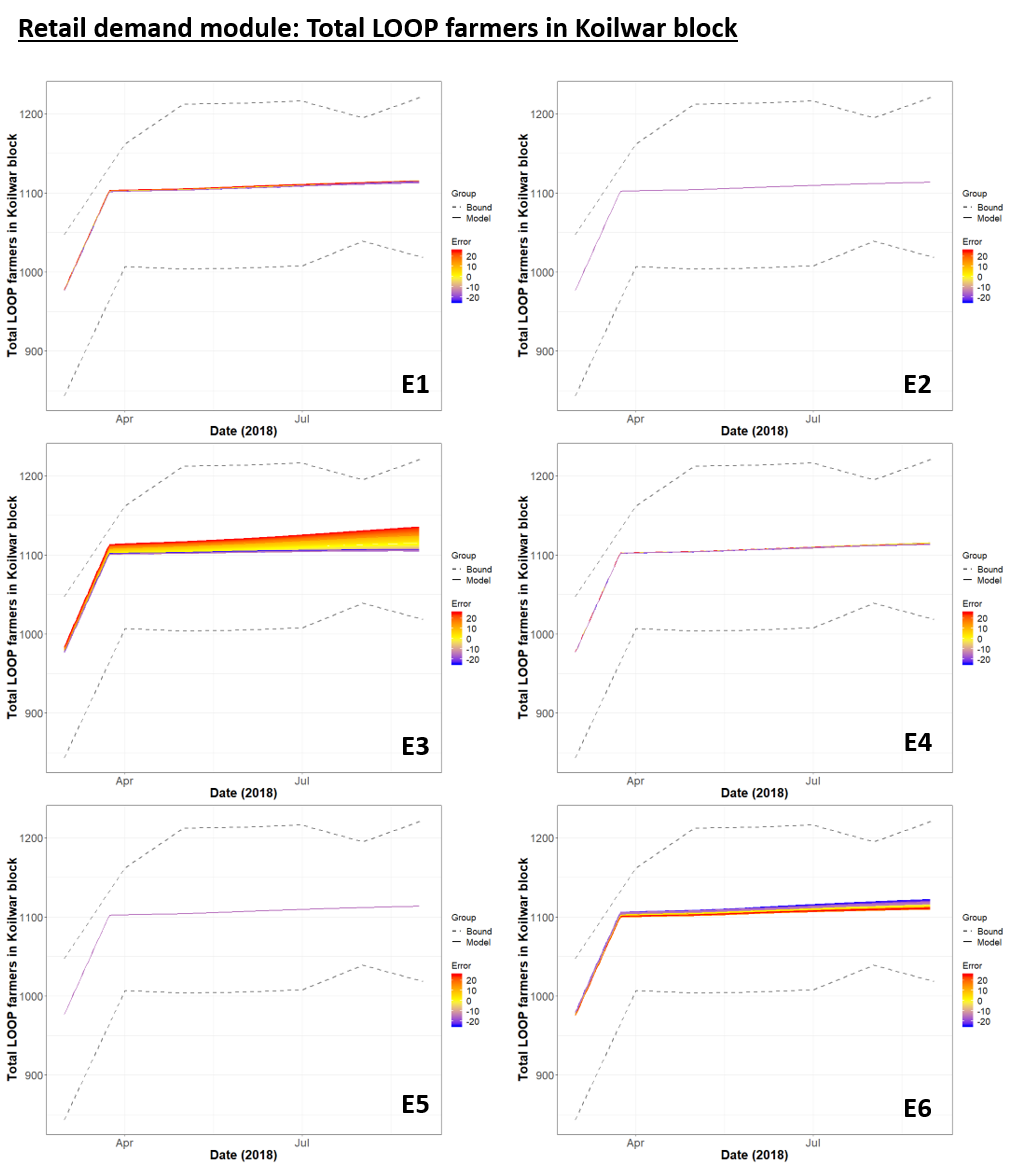 |
| --- |
| Figure S14: Sensitivity outputs from the ‘Retail demand’ module (E1-E6) – visualised as the total number of farmers in Loop. The black dashed lines have the same meaning as in figure S9a. |
| 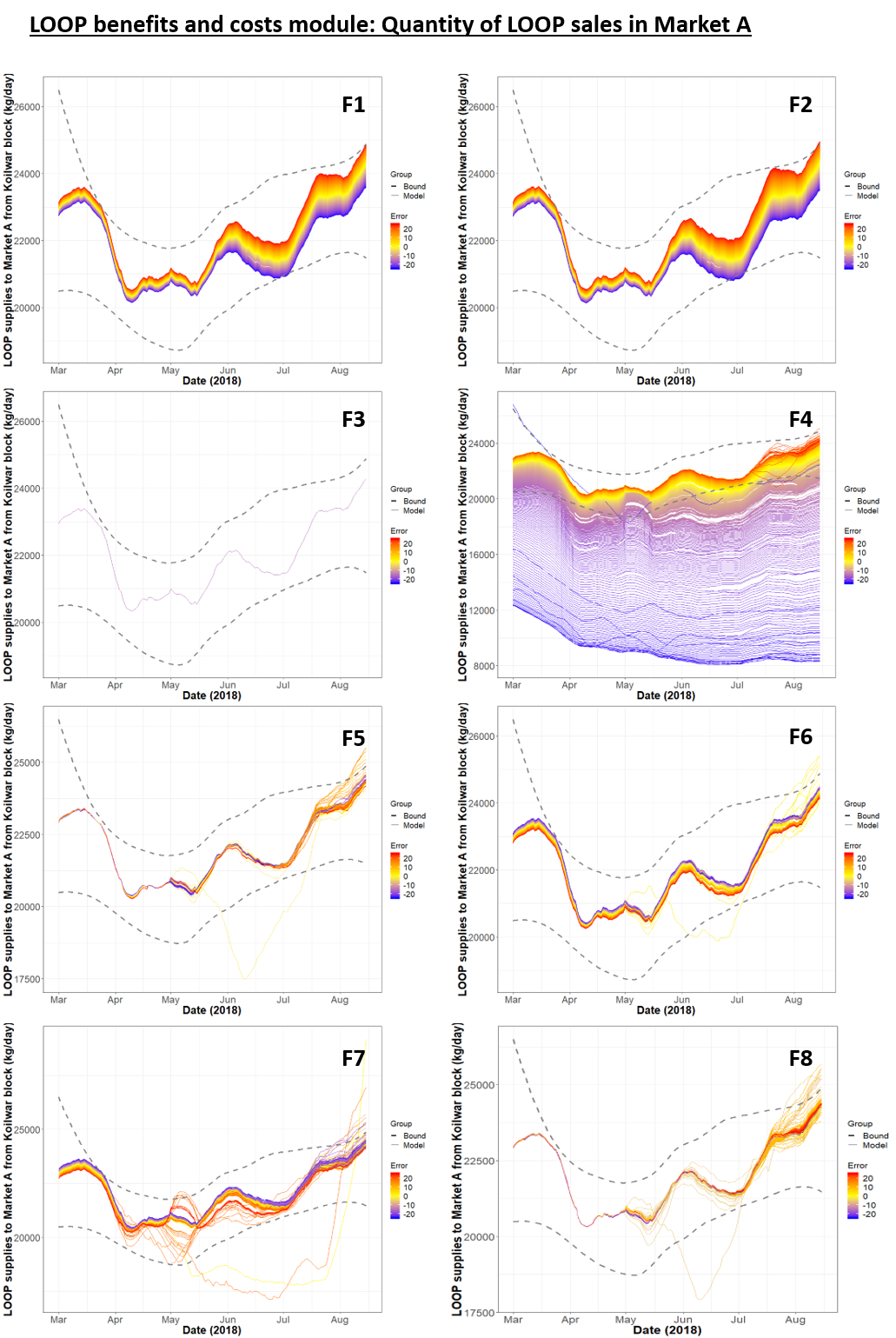 |
| Figure S15: Sensitivity outputs from the ‘Loop benefits and costs’ module (F1-F8) – visualised as the daily Loop sales in Market A. The black dashed lines have the same meaning as in figure S8a. |
| 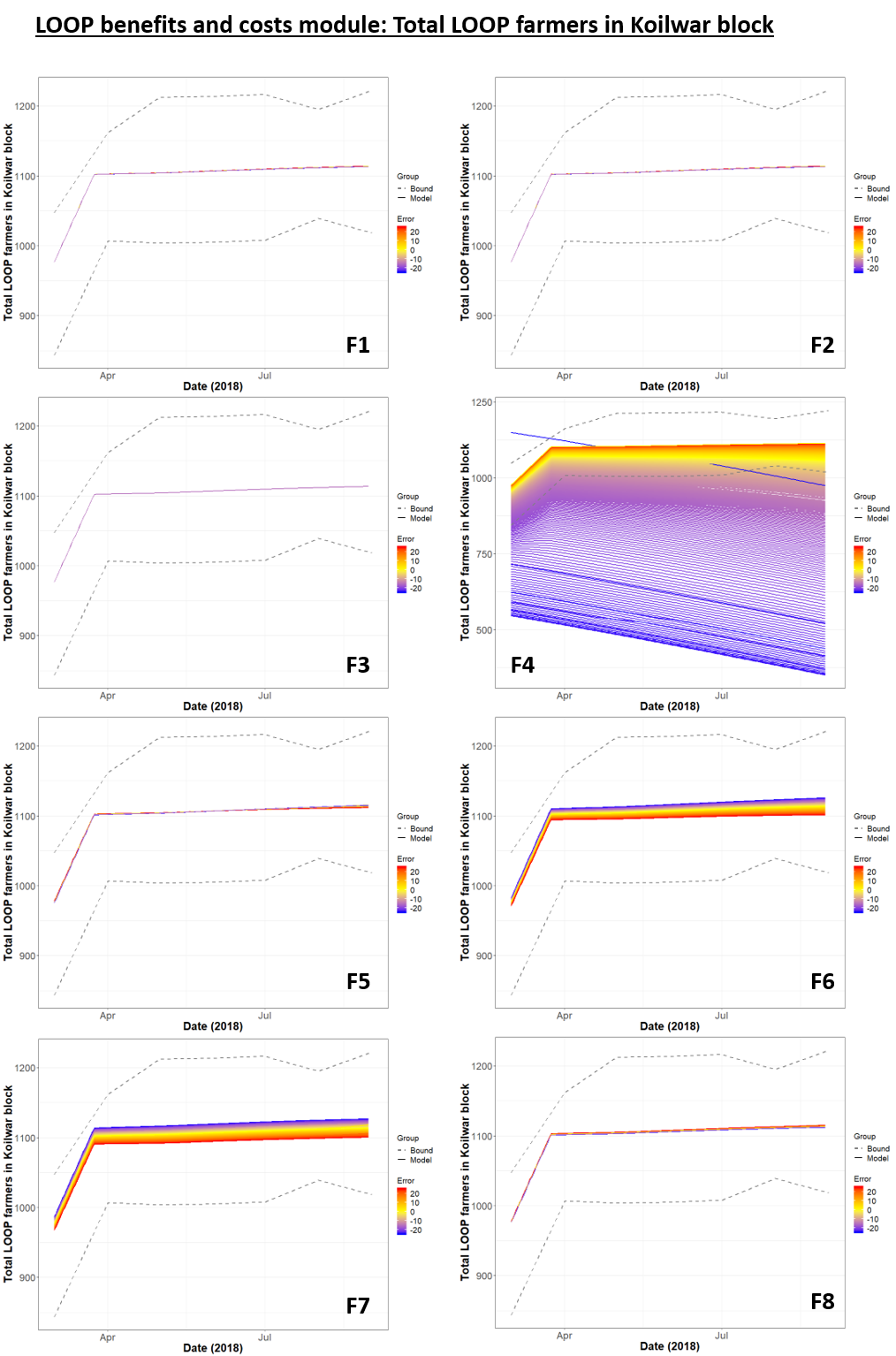 |
| Figure S16: Sensitivity outputs from the ‘Loop benefits and costs’ module (F1-F8) – visualised as the total number of farmers in Loop. The black dashed lines have the same meaning as in figure S9a. |

| 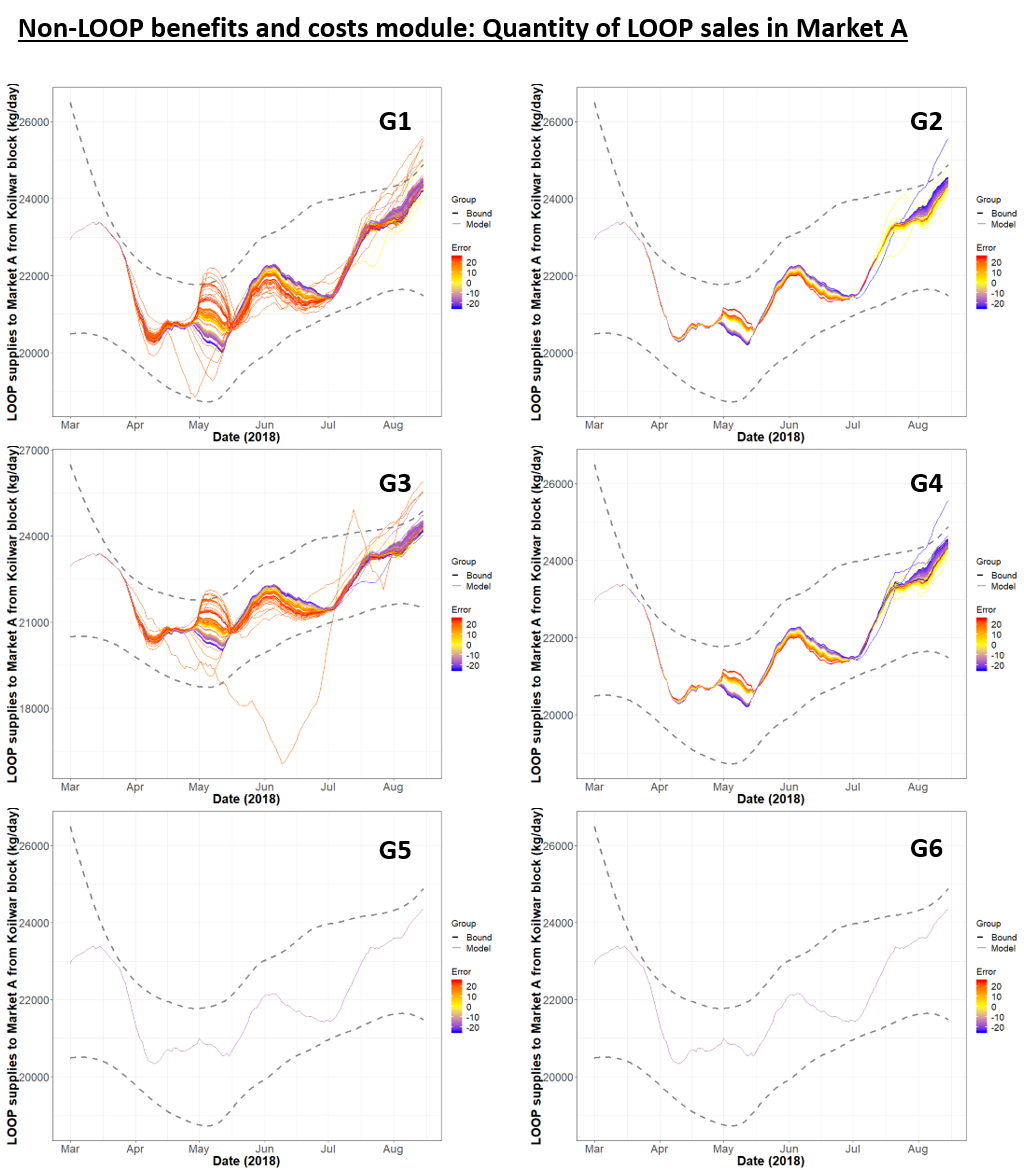 |
| --- |
| Figure S17: Sensitivity outputs from the ‘Non-Loop benefits and costs’ module (G1-G6) – visualised as the daily Loop sales in Market A. The black dashed lines have the same meaning as in figure S8a. |

| 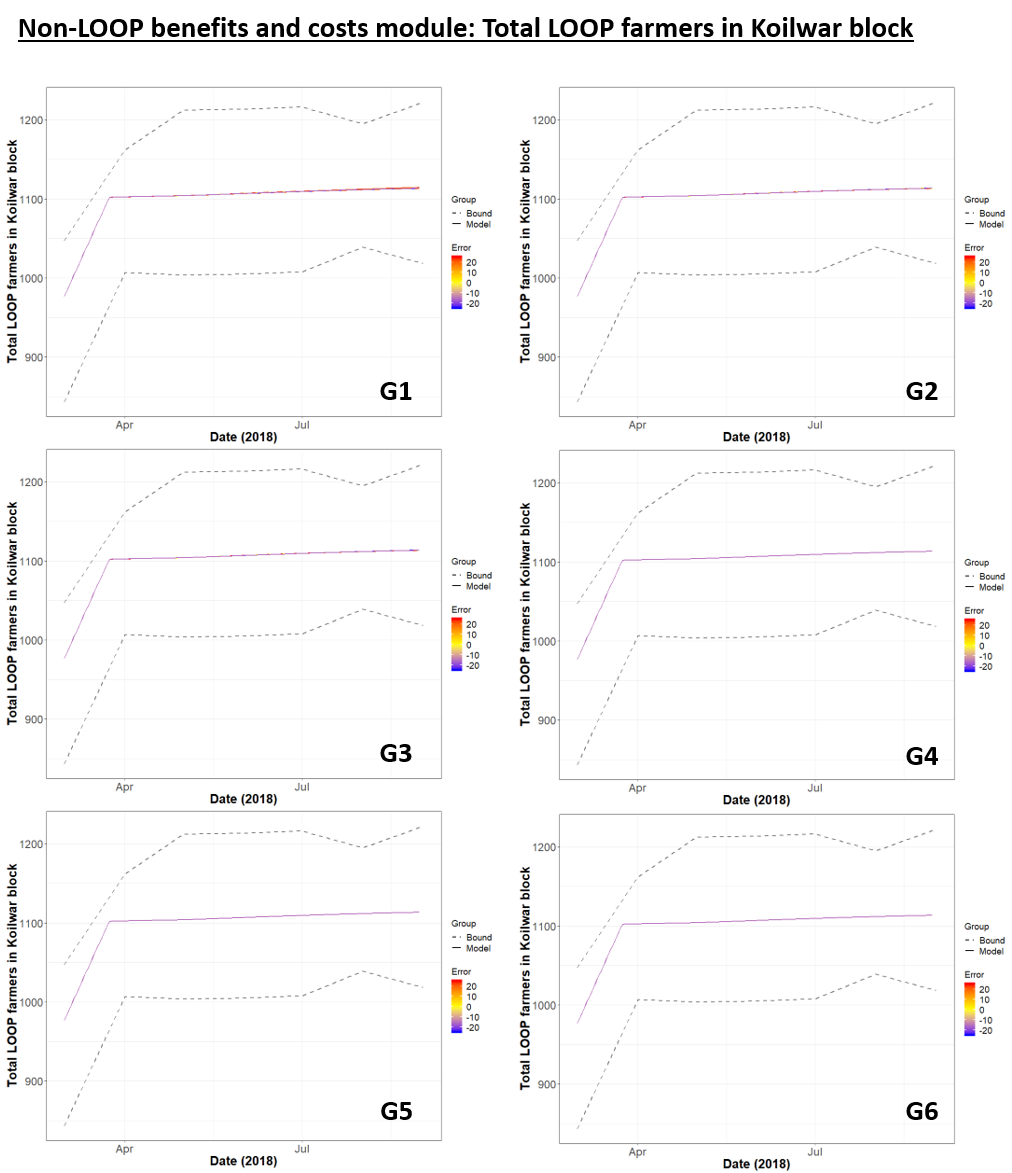 |
| --- |
| Figure S18: Sensitivity outputs from the ‘Non-Loop benefits and costs’ module (F1-F8) – visualised as the total number of farmers in Loop. The black dashed lines have the same meaning as in figure S9a. |

E.3 Kolmogorov-Smirnov tests

The list below orders the ‘yellow sensitivity’ variables by their Kolmogorov-Smirnov statistic, where the topmost variable has the greatest difference between its error values (ϵ) that generate behaviour-giving outcomes and its error ranges that do not.

Market A retail reference price: D-score = 0.413, p < 0.001.

Market A retailer profit perception time: D-score = 0.313, p < 0.001.

Market A price perception time: D-score = 0.288, p < 0.001.

Market B retailer price sensi. to inventory coverage: D-score = 0.279, p < 0.001.

Market A local trader price sensi. to inventory coverage: D-score = 0.261, p < 0.001.

Market A local trader profit perception time: D-score = 0.260, p < 0.001.

The list below orders the ‘red sensitivity’ variables by their Kolmogorov-Smirnov statistic, where the topmost variable has the greatest difference between its error values (ϵ) that generate behaviour-giving outcomes and its error ranges that do not.

Non-Loop market A random: D-score = 0.438, p < 0.001.

Non-Loop Market A proportion: D-score = 0.422, p < 0.001.

Market A retailer price sensitivity to inventory coverage: D-score = 0.313, p < 0.001.

Loop utility perception time: D-score = 0.297, p < 0.001.

Non-Loop Market B random: D-score = 0.294, p < 0.001.

Market B retail reference price: D-score = 0.286, p < 0.001.

Non-Loop Market B proportion: D-score = 0.254, p < 0.001.

Market A retailer price sensitivity to costs: D-score = 0.213, p < 0.001.

Supplmentary Material F: Model equations and parameter values

Note: the acronym ‘NL’ – ‘non-Loop’ (e.g. farmers, F&V supplies, revenues); ‘Rs’ – ‘Indian rupees’; ‘DT’ – ‘distance trader’; ‘LT’ – ‘local wholesale trader’; ‘Ret’ – ‘Retailer’

F.1 Scenario Hub

ColdStorageSCENARIO = IF TIME > 669 AND ColdStorageActiveSCENARIO >=0.5 THEN 1 ELSE 0

UNITS: Dimensionless

ColdStorageActiveSCENARIO = 0

UNITS: Dimensionless

ColdStorageInvestmentLevelSCENARIO = 0

UNITS: Dimensionless

ExternalRetailReferenceDemandSCENARIO = 0

UNITS: Dimensionless

LOOPExtensionRateSCENARIO = 0

UNITS: Dimensionless

MarketBSubsidySCENARIO = 0

UNITS: Dimensionless

F.2 Farmer Household

Farmer Population and Adoption

KoilwarFarmingHouseholds(t) = KoilwarFarmingHouseholds(t - dt)

INIT KoilwarFarmingHouseholds = 12087

UNITS: People

LOOPFarmersKoilwarBlock(t) = LOOPFarmersKoilwarBlock(t - dt) + (LOOPAdopters - LOOPDisadopters) * dt

INIT LOOPFarmersKoilwarBlock = 90

UNITS: People

INFLOWS:

LOOPAdopters = (AdoptingWordMouth + AdoptingExtension)*(Trust&Utility.LOOPTrust*Trust&Utility.LongtermLOOPUtility)

UNITS: People/halfday

OUTFLOWS:

LOOPDisadopters = (BaseDisadoptionRate+BaseDisadoptionRate*DirectDisadoption)

UNITS: People/halfday

AdoptingExtension = IF TIME < 669 THEN ExtensionEffectiveness * NonLOOPFarmersKoilwarBlock*ExtensionOn ELSE "A.ScenarioHub".LOOPExtensionRateSCENARIO*NonLOOPFarmersKoilwarBlock

UNITS: Dimensionless

AdoptingWordMouth = (ContactRate*AdoptionFraction*NonLOOPFarmersKoilwarBlock*LOOPFarmersKoilwarBlock)/KoilwarFarmingHouseholds

UNITS: People

AdoptionFraction = 0.0001

UNITS: Dimensionless

BaseDisadoptionRate = 0.2

UNITS: People

ContactRate = 2

UNITS: Dimensionless

DirectDisadoption = IF SMTH1(1-Trust&Utility.LongtermLOOPUtility, 240)+SMTH1(1-Trust&Utility.LOOPTrust, 240) < 0 THEN 0 ELSE SMTH1(1-Trust&Utility.LongtermLOOPUtility, 240)+SMTH1(Trust&Utility.LOOPTrust, 240)

UNITS: Dimensionless

ExtensionEffectiveness = 0.00025

UNITS: Dimensionless

ExtensionOn = IF TIME < 350 THEN 1 ELSE 0

UNITS: Dimensionless

HarvestFrequency = GRAPH(TIME) (1, 4.0), (152, 10.0), (394, 14.0), (610, 16.0), (882, 10.0), (1124, 14.0), (1340, 16.0), (1612, 10.0), (1854, 14.0), (2070, 16.0), (2342, 10.0), (2584, 14.0), (2800, 16.0), (3072, 10.0)

UNITS: Halfday

LOOPFarmersSupplyingAnyGivenDay = (LOOPFarmersKoilwarBlock)/HarvestFrequency

UNITS: People

NonLOOPFarmersGivenDay = NonLOOPFarmersKoilwarBlock/HarvestFrequency

UNITS: People

NonLOOPFarmersKoilwarBlock = KoilwarFarmingHouseholds-LOOPFarmersKoilwarBlock

UNITS: People

ProportionFemaleLOOPFarmers = 0.05

UNITS: Dimensionless

ProportionFemaleNLFarmers = 0.1

UNITS: Dimensionless

Farm Household Consumption

GenderConsumptionDifference = 0.1

UNITS: Dimensionless

LOOPDayConsumption = OriginalLOOPDayHHConsumption*(1+FarmerPopulationAndAdoption.ProportionFemaleLOOPFarmers*GenderConsumptionDifference)

UNITS: kg/capita/day

LOOPF&VDemand = LOOPHalfDayConsumption*FarmerPopulationAndAdoption.LOOPFarmersKoilwarBlock

UNITS: kg/halfday

LOOPHalfDayConsumption = LOOPDayConsumption/2

UNITS: kg/capita/halfday

NonLOOPDayConsumption = OriginalNonLOOPDayHHConsumption*(1+FarmerPopulationAndAdoption.ProportionFemaleNLFarmers*GenderConsumptionDifference)

UNITS: kg/capita/day

NonLOOPF&VDemand = NonLOOPHalfDayConsumption*FarmerPopulationAndAdoption.NonLOOPFarmersKoilwarBlock

UNITS: kg/halfday

NonLOOPHalfDayConsumption = NonLOOPDayConsumption/2

UNITS: kg/capita/halfday

OriginalLOOPDayHHConsumption = 0.380

UNITS: kg/capita/day

OriginalNonLOOPDayHHConsumption = 0.331

UNITS: kg/capita/day

F.3 Loop Benefits & Costs

Benefits and Costs Market A

AverageProfitPerLOOPFarmer = AverageRevenuePerLOOPFarmer-MarketACostsPerLOOPFarmer

UNITS: Rs

AverageRevenuePerLOOPFarmer = IF LOOPMarketable.LOOPMarketAFarmers=0 THEN 0 ELSE TotalLoopRevenueMarketA/LOOPMarketable.LOOPMarketAFarmers

UNITS: Rs

DTQuantityWeight = IF SumLOOPQuantity= 0 THEN 0 ELSE LOOPDTQuantity/SumLOOPQuantity

UNITS: Dimensionless

DTWeightedPrice = DTQuantityWeight*DistanceTrader.QualityAdjDTBuyingPrice

UNITS: Rs/kg

LOOPDTQuantity = DistanceTrader.DTLOOPSold

UNITS: kg

LOOPFarmersSupplyingMarketA = DistanceTrader.LOOPFarmersSupplyingDT+ LocalTrader.LOOPFarmersSupplyingLT+LocalRetailer.LOOPFarmersSupplyingRetail

UNITS: People

LOOPLTQuantity = LocalTrader.LTLOOPSold

UNITS: kg

LOOPRetailerQuantity = LocalRetailer.LOOPRetailerSold

UNITS: kg

LTQuantityWeight = IF SumLOOPQuantity = 0 THEN 0 ELSE LOOPLTQuantity/SumLOOPQuantity

UNITS: Dimensionless

LTWeightedPrice = LocalTrader.QualityAdjLTBuyingPrice*LTQuantityWeight

UNITS: Rs/kg

MarketACostsPerLOOPFarmer = IF LOOPMarketable.LOOPMarketAFarmers=0 THEN 0 ELSE TotalMarketALOOPCosts/(LOOPMarketable.LOOPMarketAFarmers)

UNITS: Rs

MarketALOOPProportionSold = IF LOOPMarketable.TotalMarketALOOP =0 THEN 1 ELSE (DistanceTrader.DTLOOPSold+LocalTrader.LTLOOPSold+LocalRetailer.LOOPRetailerSold) / LOOPMarketable.TotalMarketALOOP

UNITS: Dimensionless

MarketALOOPSoldUse = IF MarketALOOPProportionSold >1 THEN 1 ELSE MarketALOOPProportionSold

UNITS: Dimensionless

MarketALOOPUnsold = 1-MarketALOOPSoldUse

UNITS: Dimensionless

MarketAPerKgCost = IF SumLOOPQuantity=0 THEN 0 ELSE TotalTransportCost/SumLOOPQuantity

UNITS: Rs/kg

MarketATotalLOOP = DistanceTrader.DTLOOPSold+LocalTrader.LTLOOPSold+ LocalRetailer.LOOPRetailerSold

UNITS: kg

MarketAVehicleDemand = SumLOOPQuantity/MarketPreference.AggregatorDailyCapacity

UNITS: Vehicles

MarketAWeightedAveragePriceLOOP = DTWeightedPrice+LTWeightedPrice+RetailerWeightedPrice

UNITS: Rs/kg

MaxMarketALocalPrice = DELAY(MAX(LocalTrader.QualityAdjLTBuyingPrice, LocalRetailer.QualityAdjRetBuyingPrice), 2)

UNITS: Rs

MaxMarketAPrice = MAX(DELAY(DistanceTrader.QualityAdjDTBuyingPrice, 2), MaxMarketALocalPrice)

UNITS: Rs

RetailerQuantityWeight = IF SumLOOPQuantity = 0 THEN 0 ELSE LOOPRetailerQuantity/SumLOOPQuantity

UNITS: Dimensionless

RetailerWeightedPrice = LocalRetailer.QualityAdjRetBuyingPrice*RetailerQuantityWeight

UNITS: Rs/kg

SumLOOPQuantity = LOOPDTQuantity+LOOPLTQuantity+LOOPRetailerQuantity

UNITS: kg

TotalFarmersSupplyingMarketA = NonLOOPMarketable.NonLOOPFarmersMarketA+LOOPMarketable.LOOPMarketAFarmers

UNITS: People

TotalLOOPCommissionMarketA = GaddidarCommission.DTLOOPCommissionIncurred+GaddidarCommission.LTLOOPCommissionIncurred+GaddidarCommission.RetailerLOOPCommissionIncurred

UNITS: Rs

TotalLoopRevenueMarketA = DistanceTrader.DTLOOPRevenue+LocalTrader.LTLOOPRevenue+LocalRetailer.RetailLOOPRevenue

UNITS: Rs

TotalMarketALOOPCosts = TotalTransportCost+TotalLOOPCommissionMarketA

UNITS: Rs

TotalTransportCost = MarketAVehicleDemand*MarketPreference.MarketASingleVehicleCost

UNITS: Rs

WeightedMarketATransportCost = (MarketAPerKgCost*(LOOPMarketable.LOOPMarketAFarmers/TotalFarmersSupplyingMarketA))+(NonLOOPBenefitsMarketA.NonLOOPCostPerKg*(NonLOOPMarketable.NonLOOPFarmersMarketA/TotalFarmersSupplyingMarketA))

UNITS: Rs/kg

Benefits and Costs Market B

CumulativeLOOPProfitPerFarmer(t) = CumulativeLOOPProfitPerFarmer(t - dt) + (CumulativeLOOPProfitChange - LOOPLandInvestment - LOOPYieldInvestment) * dt

INIT CumulativeLOOPProfitPerFarmer = 0

UNITS: Rs

INFLOWS:

CumulativeLOOPProfitChange = WeightedLOOPProfitPerFarmer/FarmerPopulationAndAdoption.HarvestFrequency

UNITS: Rs/halfday

OUTFLOWS:

LOOPLandInvestment = IF NonLOOPBenefitsMarketB.SeasonChange = 0 OR CumulativeLOOPProfitPerFarmer < NonLOOPBenefitsMarketB.PriceTenthKatha/LOOPLandInvestmentRate OR SeasonalSmoothProfits <0 THEN 0 ELSE CumulativeLOOPProfitPerFarmer*LOOPLandInvestmentRate

UNITS: Rs/halfday

LOOPYieldInvestment = IF NonLOOPBenefitsMarketB.SeasonChange = 0 OR SeasonalSmoothProfits <0 THEN 0 ELSE CumulativeLOOPProfitPerFarmer*LOOPYieldInvestmentRate

UNITS: Rs/halfday

LOOPYieldChange(t) = LOOPYieldChange(t - dt) + (LOOPYieldChangeFlow) * dt

INIT LOOPYieldChange = 1

UNITS: Dimensionless

INFLOWS:

LOOPYieldChangeFlow = YieldInvestmentConversion

UNITS: Dimensionless/halfday

HalfDaysPerSeason = GRAPH(TIME) (0, 302.0), (302, 184.0), (486, 244.0), (730, 302.0), (1032, 184.0), (1216, 244.0), (1460, 302.0), (1762, 184.0), (1946, 244.0), (2190, 302.0), (2492, 184.0), (2676, 244.0), (2978, 302.0)

UNITS: Halfdays

LOOPLandExpansion = LOOPLandInvestment / NonLOOPBenefitsMarketB.PriceTenthKatha

UNITS: katha

LOOPLandInvestmentRate = 2/10

UNITS: Dimensionless

LOOPMarketBPerKgCost = IF MarketBSumLOOPQuantity= 0 THEN 0 ELSE TotalTransportCostMarketB/MarketBSumLOOPQuantity

UNITS: Rs/kg

LOOPMarketBSales = MarketBLocalTrader.MarketBLTLOOPSold+MarketBLocalRetailer.MarketBLOOPRetailerSold

UNITS: kg

LOOPYieldInvestmentRate = 1/10

UNITS: Dimensionless

MarketAProportion = IF LOOPMarketable.TotalMarketBLOOP = 0 AND LOOPMarketable.TotalMarketALOOP=0 THEN 0 ELSE LOOPMarketable.TotalMarketALOOP/(LOOPMarketable.TotalMarketBLOOP+LOOPMarketable.TotalMarketALOOP)

UNITS: Dimensionless

MarketAUnsoldWeighted = IF (LOOPMarketable.TotalMarketBLOOP+ LOOPMarketable.TotalMarketALOOP) = 0 THEN 0 ELSE BenefitsMarketA.MarketALOOPUnsold*(LOOPMarketable.TotalMarketALOOP/(LOOPMarketable.TotalMarketALOOP+LOOPMarketable.TotalMarketBLOOP))

UNITS: Dimensionless

MarketBAverageProfitPerLOOPFarmer = MarketBAverageRevenuePerLOOPFarmer-MarketBCostsPerLOOPFarmer

UNITS: Rs

MarketBAverageRevenuePerLOOPFarmer = IF MarketPreference.MarketAPreferenceOverBUse = 1 OR LOOPMarketable.LOOPMarketBFarmers =0 THEN 0 ELSE TotalLoopRevenueMarketB/LOOPMarketable.LOOPMarketBFarmers

UNITS: Rs

MarketBCostsPerLOOPFarmer = IF MarketPreference.MarketAPreferenceOverBUse=1 OR LOOPMarketable.LOOPMarketBFarmers=0 THEN 0 ELSE TotalMarketBLOOPCosts/LOOPMarketable.LOOPMarketBFarmers

UNITS: Rs

MarketBLOOPLTQuantity = MarketBLocalTrader.MarketBLTLOOPSold

UNITS: kg

MarketBLOOPRetailerQuantity = MarketBLocalRetailer.MarketBLOOPRetailerSold

UNITS: kg

MarketBLOOPSold = IF LOOPMarketable.TotalMarketBLOOP = 0 THEN 1 ELSE (MarketBLocalTrader.MarketBLTLOOPSold+MarketBLocalRetailer.MarketBLOOPRetailerSold)/LOOPMarketable.TotalMarketBLOOP

UNITS: Dimensionless

MarketBLOOPUnsold = IF LOOPMarketable.TotalMarketBLOOP= 0 THEN 0 ELSE 1-MarketBLOOPSold

UNITS: Dimensionless

MarketBLTQuantityWeight = IF MarketBSumLOOPQuantity = 0 THEN 0 ELSE MarketBLOOPLTQuantity/MarketBSumLOOPQuantity

UNITS: Dimensionless

MarketBMaxPrice = MAX(MarketBLocalTrader.QualityAdjLTBBuyingPrice, MarketBLocalRetailer.QualityAdjRetBBuyingPrice)

UNITS: Rs/kg

MarketBRetailerQuantityWeight = IF MarketBSumLOOPQuantity = 0 THEN 0 ELSE MarketBLOOPRetailerQuantity/MarketBSumLOOPQuantity

UNITS: Dimensionless

MarketBRetailerWeightedPrice = MarketBLocalRetailer.QualityAdjRetBBuyingPrice*MarketBRetailerQuantityWeight

UNITS: Rs/kg

MarketBSumLOOPQuantity = MarketBLOOPRetailerQuantity+MarketBLOOPLTQuantity

UNITS: kg

MarketBUnsoldWeighted = IF (LOOPMarketable.TotalMarketBLOOP+LOOPMarketable.TotalMarketALOOP) = 0 THEN 0 ELSE MarketBLOOPUnsold*(LOOPMarketable.TotalMarketBLOOP/(LOOPMarketable.TotalMarketBLOOP+LOOPMarketable.TotalMarketALOOP))

UNITS: Dimensionless

MarketBVehicleDemand = MarketBSumLOOPQuantity/MarketPreference.AggregatorDailyCapacity

UNITS: Vehicles

MarketBWeightedAveragePriceLOOP = MarketNLTWeightedPrice+MarketBRetailerWeightedPrice

UNITS: Rs/kg

MarketNLTWeightedPrice = MarketBLocalTrader.QualityAdjLTBBuyingPrice* MarketBLTQuantityWeight

UNITS: Rs/kg

SeasonalSmoothProfits = SMTH1(WeightedLOOPProfitPerFarmer-DELAY(WeightedLOOPProfitPerFarmer, HalfDaysPerSeason), HalfDaysPerSeason)

UNITS: Rs

TotalFarmersSupplyingMarketB = NonLOOPMarketable.NonLOOPFarmersMarketB+LOOPMarketable.LOOPMarketBFarmers

UNITS: People

TotalLOOPCommissionMarketB = MarketBGaddidarCommission.MarketBLTLOOPCommissionIncurred+MarketBGaddidarCommission.MarketBRetailerLOOPCommissionIncurred

UNITS: Rs

TotalLOOPFarmersMarketB = MarketBLocalTrader.LOOPFarmersSupplyingLT+MarketBLocalRetailer.MarketBRetailLOOPFarmersSupplying

UNITS: People

TotalLOOPFarmersWithSales = BenefitsMarketA.LOOPFarmersSupplyingMarketA+TotalLOOPFarmersMarketB

UNITS: People

TotalLOOPRevenue = BenefitsMarketA.TotalLoopRevenueMarketA+TotalLoopRevenueMarketB

UNITS: Rs

TotalLoopRevenueMarketB = MarketBLocalTrader.MarketBLTLOOPRevenue+MarketBLocalRetailer.MarketBRetailLOOPRevenue

UNITS: Rs

TotalLOOPSales = BenefitsMarketA.MarketATotalLOOP+LOOPMarketBSales

UNITS: kg

TotalLOOPSupply = MarketBSumLOOPQuantity+BenefitsMarketA.SumLOOPQuantity

UNITS: kg

TotalLOOPUnsoldWeighted = MarketBUnsoldWeighted+MarketAUnsoldWeighted

UNITS: Dimensionless

TotalMarketBLOOPCosts = TotalTransportCostMarketB+TotalLOOPCommissionMarketB

UNITS: Rs

TotalSalesPerLOOPFarmer = IF TotalLOOPFarmersWithSales < 1 THEN 0 ELSE TotalLOOPSales/TotalLOOPFarmersWithSales

UNITS: kg

TotalTransportCostMarketB = MarketBVehicleDemand*MarketPreference.MarketBSingleVehicleCost

UNITS: Rs

WeightedLOOPProfitMarketA = IF BenefitsMarketA.SumLOOPQuantity = 0 THEN 0 ELSE BenefitsMarketA.AverageProfitPerLOOPFarmer*(BenefitsMarketA.SumLOOPQuantity/TotalLOOPSupply)

UNITS: Rs

WeightedLOOPProfitMarketB = IF MarketBSumLOOPQuantity = 0 THEN 0 ELSE MarketBAverageProfitPerLOOPFarmer*(MarketBSumLOOPQuantity/TotalLOOPSupply)

UNITS: Rs

WeightedLOOPProfitPerFarmer = IF (WeightedLOOPProfitMarketB+WeightedLOOPProfitMarketA) = 0 THEN 0 ELSE (WeightedLOOPProfitMarketB+WeightedLOOPProfitMarketA) -OnfarmCosts.LOOPTotalHalfDayCosts

UNITS: Rs

WeightedLOOPTransportCost = WeightedTransportCostMarketB+WeightedTransportCostMarketA

UNITS: Rs/kg

WeightedMarketBTransportCost = (LOOPMarketBPerKgCost*(LOOPMarketable.LOOPMarketBFarmers/TotalFarmersSupplyingMarketB))+(NonLOOPBenefitsMarketA.NonLOOPCostPerKg*(NonLOOPMarketable.NonLOOPFarmersMarketB/TotalFarmersSupplyingMarketB))

UNITS: Rs/kg

WeightedMeanLOOPPrice = IF TotalLOOPSupply = 0 THEN 0 ELSE (BenefitsMarketA.MarketAWeightedAveragePriceLOOP*(BenefitsMarketA.MarketATotalLOOP/TotalLOOPSupply))+(MarketBWeightedAveragePriceLOOP*(MarketBSumLOOPQuantity/TotalLOOPSupply))

UNITS: Rs/kg

WeightedTransportCostMarketA = IF BenefitsMarketA.SumLOOPQuantity = 0 THEN 0 ELSE BenefitsMarketA.MarketAPerKgCost*(BenefitsMarketA.SumLOOPQuantity/TotalLOOPSupply)

UNITS: Rs/kg

WeightedTransportCostMarketB = IF MarketBSumLOOPQuantity = 0 THEN 0 ELSE LOOPMarketBPerKgCost*(MarketBSumLOOPQuantity/TotalLOOPSupply)

UNITS: Rs/kg

YieldInvestmentConversion = GRAPH(LOOPYieldInvestment)

(0, 0.000), (20000, 0.010)

UNITS: Dimensionless

Loop ROI

AggregatorEnumerationRate = 0.1

UNITS: Rs/kg

ROIfactor = IF TotalAggregationEnumeration = 0 THEN 0 ELSE TotalTransportCostsCollected/TotalAggregationEnumeration

UNITS: Dimensionless

ROInet = TotalTransportCostsCollected-TotalAggregationEnumeration

UNITS: Rs

TotalAggregationEnumeration = BenefitsMarketB.TotalLOOPSales*AggregatorEnumerationRate

UNITS: Rs

TotalTransportCostsCollected = BenefitsMarketB.TotalLOOPSales*BenefitsMarketB.WeightedLOOPTransportCost

UNITS: Rs

Trust & utility

LongtermLOOPUtility(t) = LongtermLOOPUtility(t - dt) + (ChangeLOOPUtility) * dt

INIT LongtermLOOPUtility = 1

UNITS: Dimensionless

INFLOWS:

ChangeLOOPUtility = IF LOOPMarketable.MorningIndicator < 1 THEN 0 ELSE (SalesUtilityWeight*LOOPNonLOOPSoldUtility+ProfitUtilityWeight*LOOPNonLOOPProfitUtility)/LOOPUtilityPerceptionTime

UNITS: Dimensionless/halfday

LOOPTrust(t) = LOOPTrust(t - dt) + (LOOPTrustChange) * dt

INIT LOOPTrust = 1

UNITS: Dimensionless

INFLOWS:

LOOPTrustChange = IF PerceivedChangeProfit = 0 AND PerceivedChangeInPct = 0 AND BenefitsMarketA.SumLOOPQuantity = 0 AND BenefitsMarketB.MarketBSumLOOPQuantity = 0 AND LOOPMarketable.MorningIndicator = 1 THEN -0.1/TrustPerceptionTime ELSE (LOOPProfitWeight*PerceivedChangeProfit+LOOPSalesWeight*PerceivedChangeInPct)/TrustPerceptionTime

UNITS: Dimensionless/halfday

LastMonthProfit = DELAY(BenefitsMarketB.WeightedLOOPProfitPerFarmer, 60)

UNITS: Dimensionless

LastMonthsSoldPct = DELAY(LOOPSoldPct, 60)

UNITS: Dimensionless

LOOPNonLOOPProfitUtility = IF BenefitsMarketB.WeightedLOOPProfitPerFarmer = 0 OR NonLOOPBenefitsMarketB.WeightedNonLOOPProfitPerFarmer = 0 OR LOOPMarketable.MorningIndicator <1 THEN 0 ELSE (BenefitsMarketB.WeightedLOOPProfitPerFarmer/(BenefitsMarketB.WeightedLOOPProfitPerFarmer+NonLOOPBenefitsMarketB.WeightedNonLOOPProfitPerFarmer))-0.5

UNITS: Dimensionless

LOOPNonLOOPSoldUtility = IF LOOPSoldPct = 0 THEN 0 ELSE (LOOPSoldPct/(LOOPSoldPct+NonLOOPSoldPct))-0.5

UNITS: Dimensionless

LOOPProfitWeight = 0.5

UNITS: Dimensionless

LOOPSalesWeight = 1

UNITS: Dimensionless

LOOPSoldPct = IF LOOPMarketable.TotalMarketALOOP=0 AND LOOPMarketable.TotalMarketBLOOP=0 THEN 1 ELSE (BenefitsMarketA.SumLOOPQuantity+BenefitsMarketB.MarketBSumLOOPQuantity)/(LOOPMarketable.TotalMarketALOOP+LOOPMarketable.TotalMarketBLOOP)

UNITS: Dimensionless

LOOPUtilityPerceptionTime = 60

UNITS: halfday

NonLOOPSoldPct = IF NonLOOPMarketable.TotalNonLOOPMarketB=0 OR NonLOOPMarketable.TotalNonLOOPMarketA=0 THEN 1 ELSE (NonLOOPBenefitsMarketA.SumNonLOOPQuantity+NonLOOPBenefitsMarketB.SumNonLOOPQuantityMarketB)/(NonLOOPMarketable.TotalNonLOOPMarketA+NonLOOPMarketable.TotalNonLOOPMarketB)

UNITS: Dimensionless

PerceivedChangeInPct = IF LOOPSoldPct = 0 OR LastMonthsSoldPct = 0 THEN 0 ELSE (LOOPSoldPct-LastMonthsSoldPct)/LastMonthsSoldPct

UNITS: Dimensionless

PerceivedChangeProfit = IF BenefitsMarketB.WeightedLOOPProfitPerFarmer = 0 OR LastMonthProfit = 0 THEN 0 ELSE (BenefitsMarketB.WeightedLOOPProfitPerFarmer - LastMonthProfit)/LastMonthProfit

UNITS: Rs

ProfitUtilityWeight = 0.5

UNITS: Dimensionless

SalesUtilityWeight = 1

UNITS: Dimensionless

TrustPerceptionTime = 60

UNITS: halfday

F.4 Market Supply

Large External Market

DistanceMarketPrice = GRAPH(TIME) (1, 38.0), (62, 39.0), (122, 24.0), (184, 16.0), (246, 24.0), (302, 25.0), (364, 17.0), (424, 30.0), (486, 28.0), (546, 33.0), (608, 35.0), (670, 35.0), (730, 38.0), (792, 39.0), (852, 24.0), (914, 16.0), (976, 40.0), (1038, 39.0), (1100, 24.0), (1162, 16.0), (1224, 24.0), (1286, 25.0), (1348, 17.0), (1410, 30.0), (1472, 28.0), (1534, 33.0), (1596, 35.0), (1658, 35.0), (1720, 38.0), (1782, 39.0), (1844, 24.0), (1906, 16.0), (1968, 40.0), (2030, 39.0), (2092, 24.0), (2154, 16.0), (2216, 24.0), (2278, 25.0), (2340, 17.0), (2402, 30.0), (2464, 28.0), (2526, 33.0), (2588, 35.0), (2650, 35.0), (2712, 38.0), (2774, 39.0), (2836, 24.0), (2898, 16.0), (2960, 35.0)

UNITS: Rs/kg

DistanceTraderExpectedPrice = DistanceMarketPrice

UNITS: Rs/kg

Market A

***Distance Trader***

DistanceTrader:

CumulativeDTCosts(t) = CumulativeDTCosts(t - dt) + (DTCostsBiflow) * dt

INIT CumulativeDTCosts = 1

UNITS: Rs

INFLOWS:

DTCostsBiflow = SMTH1(CostPerDT, 60)

UNITS: Rs/halfday

CumulativeDTRevenue(t) = CumulativeDTRevenue(t - dt) + (DTRevenueBiflow) * dt

INIT CumulativeDTRevenue = 1

UNITS: Rs

INFLOWS:

DTRevenueBiflow = RevenuePerDT

UNITS: Rs/halfday

DistanceTraderSupplyStock(t) = DistanceTraderSupplyStock(t - dt) + (MarketADistanceTraderLOOP + MarketADistanceNonLOOP - DistanceTraderDownstreamFlow - ExcessDistanceTraderSupply) * dt

INIT DistanceTraderSupplyStock = MarketADTDemand

UNITS: kg

INFLOWS:

MarketADistanceTraderLOOP = PULSE(LOOPMarketableVolume*MarketPreference.MarketAPreferenceOverBUse*TraderPreference.LOOPDistanceTraderSupplyPreferenceUSE, 1, 2)

UNITS: kg/halfday

MarketADistanceNonLOOP = IF LOOPMarketable.TotalLOOPAggregation > 0 THEN NonLOOPMarketableF&V*NonLOOPMarketAProportion*TraderPreference.NonLOOPDTSupplyPreferenceUSE ELSE 0

UNITS: kg/halfday

OUTFLOWS:

DistanceTraderDownstreamFlow = MarketADTDemand*(1-DistanceTraderWastageRate)

UNITS: kg/halfday

ExcessDistanceTraderSupply = IF TIME > 669 AND "A.ScenarioHub".ColdStorageSCENARIO >= 0.5 THEN (((1-DTAColdUtilityRate)*DistanceTraderSupplyStock)/4)+((DTAColdUtilityRate*DistanceTraderSupplyStock)/LocalRetailer.ColdStorageDuration) ELSE DistanceTraderSupplyStock/4

UNITS: kg/halfday

ColdStorageRentPerDT = IF DTHalfDay = 0 THEN 0 ELSE DTColdStorageRent/DTHalfDay

UNITS: Rs

CommissionPerDT = IF DTHalfDay = 0 THEN 0 ELSE GaddidarCommission.DTCommissionIncurred/DTHalfDay

UNITS: Rs

CostPerDT = LTOtherCostsPerDT+CommissionPerDT+VehicleCostsPerDT+FVExpenditurePerDT+ColdStorageRentPerDT

UNITS: Rs

CoveragePerceptionTime = 14

UNITS: halfday

DistanceTraderBuyingPrice = (DistanceTraderExpectedPrice*EffectCostsOnPrice*EffectInventoryCoveragePrice)/2

UNITS: Rs/kg

DistanceTraderCapacity = 2000

UNITS: kg

DistanceTraderExpectedPrice = LargeExternalMarket.DistanceTraderPrice

UNITS: Rs/kg

DistanceTraderRevenue = (DistanceTraderDownstreamFlow*DistanceTraderExpectedPrice)*(1-WastageRateEnRoute)

UNITS: Rs

DistanceTraderWastageRate = 0.05

UNITS: Dimensionless

DTAColdUtilityRate = IF DTAStockAccomodated < 1 THEN DTAStockAccomodated ELSE 1

UNITS: Dimensionless

DTAStockAccomodated = IF DistanceTraderSupplyStock = 0 THEN 0 ELSE (LocalRetailer.MarketAColdStorageCapacity*DTColdStorageProp)/DistanceTraderSupplyStock

UNITS: Dimensionless

DTColdStorageProp = IF LocalRetailer.TotalMarketASupply = 0 THEN 0 ELSE TotalDTSupply/LocalRetailer.TotalMarketASupply

UNITS: Dimensionless

DTColdStorageRent = DTAColdUtilityRate*DistanceTraderSupplyStock*MarketBLocalRetailer.TraderColdStorageRentRate

UNITS: Rs

DTFrequency = 1

UNITS: halfday

DTHalfDay = DTTotal/DTFrequency

UNITS: People

DTLOOPRevenue = ProportionLOOPDTSupply*DistanceTraderDownstreamFlow*QualityAdjDTBuyingPrice

UNITS: Rs

DTLOOPRevenuePerFarmer = IF LOOPFarmersSupplyingDT = 0 THEN 0 ELSE DTLOOPRevenue/LOOPFarmersSupplyingDT

UNITS: Rs

DTLOOPSold = IF DistanceTraderDownstreamFlow*ProportionLOOPDTSupply > DELAY(LOOPMarketable.MarketADistanceTraderLOOP, 2) + LOOPMarketable.MarketADistanceTraderLOOP THEN DELAY(LOOPMarketable.MarketADistanceTraderLOOP, 2) + LOOPMarketable.MarketADistanceTraderLOOP ELSE DistanceTraderDownstreamFlow*ProportionLOOPDTSupply

UNITS: kg

DTNonLOOPRevenue = DistanceTraderDownstreamFlow*(1-ProportionLOOPDTSupply)*QualityAdjDTBuyingPrice

UNITS: Rs

DTNonLOOPRevenuePerFarmer = IF NonLOOPFarmersSupplyingDT = 0 THEN 0 ELSE DTNonLOOPRevenue/NonLOOPFarmersSupplyingDT

UNITS: Rs

DTNonLOOPSold = DistanceTraderDownstreamFlow-DTLOOPSold

UNITS: kg

DTProfitPerceptionTime = 14

UNITS: halfday

DTSeasonal = GRAPH(TIME) (1, 90.0), (184, 100.0), (364, 90.0), (730, 110.0), (914, 100.0), (1094, 90.0), (1460, 110.0), (1644, 100.0), (1824, 90.0), (2190, 110.0), (2374, 100.0), (2554, 90.0), (2920, 110.0), (3104, 100.0)

UNITS: People

DTTotal = EffectofExpectedProfitDesiredCap*DTSeasonal

UNITS: People

EffectCostsOnPrice = 1+SensiPriceToCosts*((SmthDTCosts/LargeExternalMarket.DistanceTraderPrice)-1)

UNITS: Dimensionless

EffectInventoryCoveragePrice = PerceivedInventoryCoverage^SensiPriceInventoryCoverage

UNITS: Dimensionless

EffectofExpectedProfitDesiredCap = GRAPH(SMTH1(InvesmentExpectedProfitability,DTProfitPerceptionTime)) (-1.000, 0.000), (-0.500, 0.300), (0.000, 1.000), (0.500, 1.500), (1.000, 1.600)

UNITS: Dimensionless

ExpectedCosts = IF DistanceTraderDownstreamFlow = 0 THEN 0 ELSE ((VehicleCostsPerDT+LTOtherCostsPerDT+CommissionPerDT+ColdStorageRentPerDT)*DTHalfDay)/DistanceTraderDownstreamFlow

UNITS: Rs/kg

ExpectedLongRunCosts = CumulativeDTCosts-DELAY(CumulativeDTCosts, 1)

UNITS: Rs

ExpectedLongRunRevenue = CumulativeDTRevenue- DELAY(CumulativeDTRevenue, 1)

UNITS: Rs

FVExpenditurePerDT = IF DTHalfDay = 0 THEN 0 ELSE (TotalDTFVExpenditure)/DTHalfDay

UNITS: Rs

InventoryCoverage = IF DistanceTraderDownstreamFlow = 0 THEN 1 ELSE DistanceTraderSupplyStock/(DistanceTraderDownstreamFlow)

UNITS: Dimensionless

InvesmentExpectedProfitability = IF ExpectedLongRunCosts = 0 OR ExpectedLongRunRevenue =0 THEN 1 ELSE (ExpectedLongRunRevenue-ExpectedLongRunCosts)/ExpectedLongRunCosts

UNITS: Rs

LongRunProfits = SMTH1(ProfitPerDT - DELAY(ProfitPerDT, DTProfitPerceptionTime), DTProfitPerceptionTime)

UNITS: Rs

LOOPDTExcess = IF ProportionLOOPDTSupply*ExcessDistanceTraderSupply > DELAY(LOOPMarketable.MarketADistanceTraderLOOP, 1) THEN LOOPMarketable.MarketADistanceTraderLOOP ELSE ProportionLOOPDTSupply*ExcessDistanceTraderSupply

UNITS: kg

LOOPDTFarmerExcess = IF LOOPMarketable.LOOPMarketAPerFarmer = 0 THEN 0 ELSE LOOPDTExcess/LOOPMarketable.LOOPMarketAPerFarmer

UNITS: People

LOOPFarmersSupplyingDT = IF LOOPMarketable.MarketADistanceTraderLOOP = 0 THEN 0 ELSE (LOOPMarketable.LOOPMarketAFarmers*TraderPreference.NonLOOPDTSupplyPreferenceUSE)-LOOPDTFarmerExcess

UNITS: People

MarketADTDemand = DTHalfDay*DistanceTraderCapacity

UNITS: kg

NonLOOPDTExcess = LOOPDTExcess-ExcessDistanceTraderSupply

UNITS: kg

NonLOOPDTFarmerExcess = IF NonLOOPMarketable.NonLOOPMarketAPerFarmer = 0 THEN 0 ELSE NonLOOPDTExcess/NonLOOPMarketable.NonLOOPMarketAPerFarmer

UNITS: People

NonLOOPFarmersSupplyingDT = (TraderPreference.NonLOOPDTSupplyPreferenceUSE*NonLOOPMarketable.NonLOOPFarmersMarketA)-NonLOOPDTFarmerExcess

UNITS: People

NonLOOPProportionSupply = 1-ProportionLOOPDTSupply

UNITS: Dimensionless

OtherCostsPerDT = NORMAL(175, 25, 43)

UNITS: Rs

PerceivedInventoryCoverage = SMTH1(InventoryCoverage, CoveragePerceptionTime)

UNITS: Dimensionless

ProfitPerDT = RevenuePerDT-CostPerDT

UNITS: Rs

ProportionLOOPDTSupply = IF NonLOOPMarketable.MarketADistanceNonLOOP =0 AND LOOPMarketable.MarketADistanceTraderLOOP = 0 THEN 0 ELSE LOOPMarketable.MarketADistanceTraderLOOP/(LOOPMarketable.MarketADistanceTraderLOOP+NonLOOPMarketable.MarketADistanceNonLOOP)

UNITS: Dimensionless

QualityAdjDTBuyingPrice = (DistanceTraderBuyingPrice*(1-ProportionLOOPDTSupply)*NonLOOPLand&Yield.SmoothNonLOOPSupplyQuality) + ((DistanceTraderBuyingPrice*QualityPriceDifference)*(1-ProportionLOOPDTSupply)*(1-NonLOOPLand&Yield.SmoothNonLOOPSupplyQuality)) + (DistanceTraderBuyingPrice*ProportionLOOPDTSupply*LOOPLand&Yield.SmoothLOOPSupplyQuality) + ((DistanceTraderBuyingPrice*QualityPriceDifference)*ProportionLOOPDTSupply*(1-LOOPLand&Yield.SmoothLOOPSupplyQuality))

UNITS: Rs/kg

QualityPriceDifference = 0.5

UNITS: Dimensionless

RevenuePerDT = IF DTHalfDay =0 THEN 0 ELSE DistanceTraderRevenue/DTHalfDay

UNITS: Rs

SensiPriceInventoryCoverage = -0.3

UNITS: Dimensionless

SensiPriceToCosts = -0.1

UNITS: Dimensionless

SmthDTCosts = SMTH1(ExpectedCosts, TraderCostPercepTime)

UNITS: Rs/kg

TotalDTFVExpenditure = DTLOOPRevenue+DTNonLOOPRevenue

UNITS: Rs

TotalDTSupply = LOOPMarketable.MarketADistanceTraderLOOP+NonLOOPMarketable.MarketADistanceNonLOOP

UNITS: kg

TraderCostPercepTime = 14

UNITS: halfday

VehicleCostsPerDT = NORMAL(550, 200, 44)

UNITS: Rs

WastageRateEnRoute = RANDOM(0.05, 0.10, 40)

UNITS: Dimensionless

***Gaddidar commission***

DTCommissionIncurred = DistanceTrader.DistanceTraderDownstreamFlow*GaddidarOutsideActorCommission

UNITS: Rs

DTFarmerCommissionIncurred = DistanceTrader.DistanceTraderDownstreamFlow*GaddidarLocalActorCommission

UNITS: Rs

DTLOOPCommissionIncurred = DTFarmerCommissionIncurred*DistanceTrader.ProportionLOOPDTSupply

UNITS: Rs

DTNonLOOPCommissionIncurred = DTFarmerCommissionIncurred-DTLOOPCommissionIncurred

UNITS: Rs

GaddidarLocalActorCommission = 0.05

UNITS: Rs/kg

GaddidarOutsideActorCommission = 0.08

UNITS: Rs/kg

LTCommissionIncurred = GaddidarLocalActorCommission*LocalTrader.LocalTraderDownstreamFlow

UNITS: Rs

LTFarmerCommissionIncurred = GaddidarLocalActorCommission*LocalTrader.LocalTraderDownstreamFlow

UNITS: Rs

LTLOOPCommissionIncurred = LocalTrader.ProportionLOOPLTSupply*LTFarmerCommissionIncurred

UNITS: Rs

LTNonLOOPCommissionIncurred = LTFarmerCommissionIncurred - LTLOOPCommissionIncurred

UNITS: Rs

MarketAMeanWeightedPrice = IF MarketATotalSales = 0 THEN 0 ELSE (DistanceTrader.QualityAdjDTBuyingPrice*(DistanceTrader.DistanceTraderDownstreamFlow/MarketATotalSales))+(LocalTrader.QualityAdjLTBuyingPrice*(LocalTrader.LocalTraderDownstreamFlow/MarketATotalSales))+(LocalRetailer.QualityAdjRetBuyingPrice*(LocalRetailer.LocalRetailerDownstreamFlow/MarketATotalSales))

UNITS: Rs/kg

MarketATotalSales = DistanceTrader.DistanceTraderDownstreamFlow+LocalTrader.LocalTraderDownstreamFlow+LocalRetailer.LocalRetailerDownstreamFlow

UNITS: kg

RetailerCommissionIncurred = LocalRetailer.LocalRetailerDownstreamFlow*GaddidarLocalActorCommission

UNITS: Rs

RetailerFarmerCommissionIncurred = GaddidarLocalActorCommission*LocalRetailer.LocalRetailerDownstreamFlow

UNITS: Rs

RetailerLOOPCommissionIncurred = LocalRetailer.ProportionLOOPRetailSupply*RetailerFarmerCommissionIncurred

UNITS: Rs

RetailerNonLOOPCommissionIncurred = RetailerFarmerCommissionIncurred-RetailerLOOPCommissionIncurred

UNITS: Rs

Local Retailer

CumulativeRetCosts(t) = CumulativeRetCosts(t - dt) + (RetCostsBiflow) * dt

INIT CumulativeRetCosts = 1

UNITS: Rs

INFLOWS:

RetCostsBiflow = SMTH1(CostPerRet, 60)

UNITS: Rs/halfday

CumulativeRetRevenue(t) = CumulativeRetRevenue(t - dt) + (RetRevenueBiflow) * dt

INIT CumulativeRetRevenue = 100

UNITS: Rs

INFLOWS:

RetRevenueBiflow = RevenuePerRet

UNITS: Rs/halfday

LocalRetailerStock(t) = LocalRetailerStock(t - dt) + (MarketARetailNonLOOP + MarketARetailLOOP + ExcessLocalTraderSupply - LocalRetailerDownstreamFlow - RetailerUnsold) * dt

INIT LocalRetailerStock = MarketARetailerDemand

UNITS: kg

INFLOWS:

MarketARetailNonLOOP = IF LOOPMarketable.TotalLOOPAggregation > 0 THEN NonLOOPMarketableF&V*TraderPreference.NonLOOPMorningRetailerPreferenceUSE*NonLOOPMarketAProportion ELSE NonLOOPMarketableF&V*NonLOOPMarketAProportion*TraderPreference.NonLOOPAfternoonRetailerSupplyPreferenceUSE

UNITS: kg/halfday

MarketARetailLOOP = PULSE(LOOPMarketableVolume*TraderPreference.LOOPMorningRetailerSupplyPrefUSE*MarketPreference.MarketAPreferenceOverBUse, 1, 2)

UNITS: kg/halfday

ExcessLocalTraderSupply = IF TIME > 669 AND "A.ScenarioHub".ColdStorageSCENARIO >= 0.5 THEN (((1-LTAColdUtilityRate)*LocalTraderStock)/4)+((LTAColdUtilityRate*LocalTraderStock)/LocalRetailer.ColdStorageDuration) ELSE LocalTraderStock/4

UNITS: kg/halfday

OUTFLOWS:

LocalRetailerDownstreamFlow = MarketARetailerDemand

UNITS: kg/halfday

RetailerUnsold = IF TIME > 669 AND "A.ScenarioHub".ColdStorageActiveSCENARIO >= 0.5 THEN (((1-RetAColdUtilityRate)*LocalRetailerStock)/4)+((RetAColdUtilityRate*LocalRetailerStock)/ColdStorageDuration) ELSE LocalRetailerStock/4

UNITS: kg/halfday

ColdStorageDuration = 42

UNITS: halfday

CommissionPerRet = IF RetailersHalfday = 0 THEN 0 ELSE GaddidarCommission.RetailerCommissionIncurred/RetailersHalfday

UNITS: Rs

CostPerRet = RetOtherCostsPerRet+CommissionPerRet+FVExpenditurePerRet+RetAStorageRentPerRetB

UNITS: Rs

EffectofExpectedProfitDesiredCap = GRAPH(SMTH1(InvesmentExpectedProfitability,RetProfitPerceptionTime)) (-1.000, 0.000), (-0.500, 0.300), (0.000, 1.000), (0.500, 1.500), (1.000, 1.600)

UNITS: Dimensionless

ExpectedLongRunCosts = CumulativeRetCosts-DELAY(CumulativeRetCosts, 1)

UNITS: Rs

ExpectedLongRunRevenue = CumulativeRetRevenue- DELAY(CumulativeRetRevenue, 1)

UNITS: Rs

FVExpenditurePerRet = IF RetailersHalfday = 0 THEN 0 ELSE (TotalRetailerFVExpenditure)/RetailersHalfday

UNITS: Rs

InvesmentExpectedProfitability = IF ExpectedLongRunCosts = 0 OR ExpectedLongRunRevenue =0 THEN 1 ELSE (ExpectedLongRunRevenue-ExpectedLongRunCosts)/ExpectedLongRunCosts

UNITS: Rs

LocalEffectCostsOnPrice = 1+RetailerSensiPriceToCosts*((SmthRetExpCosts/DistanceTrader.QualityAdjDTBuyingPrice)-1)

UNITS: Dimensionless

LongRunProfits = SMTH1(ProfitPerRet - DELAY(ProfitPerRet, RetProfitPerceptionTime), RetProfitPerceptionTime)

UNITS: Rs

LOOPFarmersSupplyingRetail = IF LOOPMarketable.MorningIndicator = 1 THEN (TraderPreference.NonLOOPMorningRetailerPreferenceUSE*LOOPMarketable.LOOPMarketAFarmers)+LocalTrader.LOOPLTFarmerExcess - LOOPRetailerFarmerExcess ELSE 0

UNITS: People

LOOPRetailerExcess = IF ProportionLOOPRetailSupply*RetailerUnsold > DELAY(LOOPMarketable.MarketARetailLOOP, 1) THEN LOOPMarketable.MarketARetailLOOP ELSE ProportionLOOPRetailSupply*RetailerUnsold

UNITS: kg

LOOPRetailerFarmerExcess = IF LOOPMarketable.LOOPMarketAPerFarmer = 0 THEN 0 ELSE LOOPRetailerExcess/LOOPMarketable.LOOPMarketAPerFarmer

UNITS: People

LOOPRetailerSold = IF ProportionLOOPRetailSupply*LocalRetailerDownstreamFlow > DELAY(LOOPMarketable.MarketARetailLOOP, 2) + LOOPMarketable.MarketARetailLOOP THEN DELAY(LOOPMarketable.MarketARetailLOOP, 2) + LOOPMarketable.MarketARetailLOOP ELSE ProportionLOOPRetailSupply*LocalRetailerDownstreamFlow

UNITS: kg

MarketAColdStorageCapacity = IF "A.ScenarioHub".ColdStorageSCENARIO >= 0.5 THEN "A.ScenarioHub".ColdStorageInvestmentLevelSCENARIO*MarketBLocalRetailer.ColdStorageUnitCapacity*MarketInvestmentDifference ELSE 0

UNITS: kg

MarketARetailerDemand = RetailersHalfday*MarketADemand.MarketAPerRetailerDemands

UNITS: kg

MarketInvestmentDifference = 5

UNITS: Dimensionless

NonLOOPFarmersSupplyingRetail = IF LOOPMarketable.MorningIndicator = 1 THEN (NonLOOPMarketable.NonLOOPFarmersMarketA*TraderPreference.NonLOOPMorningRetailerPreferenceUSE)+LocalTrader.NonLOOPLTFarmerExcess - NonLOOPLTFarmerExcess ELSE (NonLOOPMarketable.NonLOOPFarmersMarketA*TraderPreference.NonLOOPAfternoonRetailerSupplyPreferenceUSE)+LocalTrader.NonLOOPLTFarmerExcess - NonLOOPLTFarmerExcess

UNITS: People

NonLOOPLTFarmerExcess = IF NonLOOPMarketable.NonLOOPMarketAPerFarmer= 0 THEN 0 ELSE NonLOOPRetailerExcess/NonLOOPMarketable.NonLOOPMarketAPerFarmer

UNITS: People

NonLOOPRetailerExcess = RetailerUnsold-LOOPRetailerExcess

UNITS: kg

NonLOOPRetailerSold = LocalRetailerDownstreamFlow-LOOPRetailerSold

UNITS: kg

PerceivedRetailerInventoryCoverage = SMTH1(RetailerInventoryCoverage, RetailerCoveragePerceptionTime)

UNITS: Dimensionless

ProfitPerRet = RevenuePerRet-CostPerRet

UNITS: Rs

ProportionLOOPRetailSupply = IF LOOPMarketable.MarketARetailLOOP = 0 AND LocalTrader.LOOPLTExcess = 0 THEN 0 ELSE (LOOPMarketable.MarketARetailLOOP+ LocalTrader.LOOPLTExcess)/(LOOPMarketable.MarketARetailLOOP+NonLOOPMarketable.MarketARetailNonLOOP+LocalTrader.LOOPLTExcess+LocalTrader.NonLOOPLTExcess)

UNITS: Dimensionless

QualityAdjRetBuyingPrice = (RetailerBuyingPrice*(1-ProportionLOOPRetailSupply)* NonLOOPLand&Yield.SmoothNonLOOPSupplyQuality) + ((RetailerBuyingPrice* DistanceTrader.QualityPriceDifference)*(1-ProportionLOOPRetailSupply)*(1-NonLOOPLand&Yield.SmoothNonLOOPSupplyQuality)) + (RetailerBuyingPrice* ProportionLOOPRetailSupply*LOOPLand&Yield.SmoothLOOPSupplyQuality) + ((RetailerBuyingPrice*DistanceTrader.QualityPriceDifference)* ProportionLOOPRetailSupply*(1-LOOPLand&Yield.SmoothLOOPSupplyQuality))

UNITS: Rs/kg

RetAColdStorageRent = LocalRetailerStock*RetAColdUtilityRate*MarketBLocalRetailer.TraderColdStorageRentRate

UNITS: Rs

RetAColdUtilityRate = IF RetAStockAccomodated < 1 THEN RetAStockAccomodated ELSE 1

UNITS: Dimensionless

RetAGovtCostsHalfDay = ("A.ScenarioHub".ColdStorageInvestmentLevelSCENARIO*MarketInvestmentDifference*MarketBLocalRetailer.AnnualCostOneColdUnit)/730

UNITS: Rs

RetailAColdStorageProp = IF TotalMarketASupply = 0 THEN 0 ELSE TotalRetailSupply/TotalMarketASupply

UNITS: Dimensionless

RetailerBuyingPrice = RetailerExpectedPrice*LocalEffectCostsOnPrice*RetailerEffectInventoryCoveragePrice

UNITS: Rs/kg

RetailerCoveragePerceptionTime = 14

UNITS: halfday

RetailerEffectInventoryCoveragePrice = PerceivedRetailerInventoryCoverage^RetailerSensiPriceInventoryCoverage

UNITS: Dimensionless

RetailerExpectedCosts = IF LocalRetailerDownstreamFlow = 0 THEN 0 ELSE ((RetOtherCostsPerRet+CommissionPerRet+RetAStorageRentPerRetB)*RetailersHalfday)/LocalRetailerDownstreamFlow

UNITS: Rs/kg

RetailerExpectedPrice = (DistanceTrader.QualityAdjDTBuyingPrice)

UNITS: Rs/kg

RetailerFreq = 1

UNITS: halfday

RetailerInventoryCoverage = IF LocalRetailerDownstreamFlow = 0 THEN 0 ELSE LocalRetailerStock/LocalRetailerDownstreamFlow

UNITS: Dimensionless

RetailerMarginGraphical = GRAPH(PerceivedRetailerInventoryCoverage)

(0.000, 8.000), (1.000, 0.000)

UNITS: Rs/kg

RetailerRevenue = LocalRetailerDownstreamFlow*RetailerSellingPrice

UNITS: Rs

Retailers = GRAPH(TIME) (1, 60.0), (184, 80.0), (364, 65.0), (730, 50.0), (914, 80.0), (1094, 65.0), (1460, 50.0), (1644, 80.0), (1824, 65.0), (2190, 50.0), (2374, 80.0), (2554, 65.0), (2920, 50.0), (3104, 80.0)

UNITS: People

RetailersDay = Retailers*EffectofExpectedProfitDesiredCap

UNITS: People

RetailerSellingPrice = RetailerMarginGraphical+QualityAdjRetBuyingPrice

UNITS: Rs/kg

RetailerSensiPriceInventoryCoverage = -0.3

UNITS: Dimensionless

RetailerSensiPriceToCosts = -0.1

UNITS: Dimensionless

RetailersHalfday = RetailersDay/RetailerFreq

UNITS: People

RetailLOOPRevenue = ProportionLOOPRetailSupply*LocalRetailerDownstreamFlow*QualityAdjRetBuyingPrice

UNITS: Rs

RetailLOOPRevenuePerFarmer = IF LOOPFarmersSupplyingRetail = 0 THEN 0 ELSE RetailLOOPRevenue/LOOPFarmersSupplyingRetail

UNITS: Rs

RetailNonLOOPRevenue = LocalRetailerDownstreamFlow*(1-ProportionLOOPRetailSupply)*QualityAdjRetBuyingPrice

UNITS: Rs

RetailNonLOOPRevenuePerFarmer = IF NonLOOPFarmersSupplyingRetail = 0 THEN 0 ELSE RetailNonLOOPRevenue/NonLOOPFarmersSupplyingRetail

UNITS: Rs

RetAStockAccomodated = IF LocalRetailerStock = 0 THEN 0 ELSE (MarketAColdStorageCapacity*RetailAColdStorageProp)/LocalRetailerStock

UNITS: Dimensionless

RetAStorageRentPerRetB = IF RetailersHalfday = 0 THEN 0 ELSE RetAColdStorageRent/RetailersHalfday

UNITS: Rs

RetOtherCostsPerRet = NORMAL(10, 2.5, 45)

UNITS: Rs

RetProfitPerceptionTime = 240

UNITS: halfday

RevenuePerRet = IF RetailersHalfday =0 THEN 0 ELSE RetailerRevenue/RetailersHalfday

UNITS: Rs

SmthRetExpCosts = SMTH1(RetailerExpectedCosts, DistanceTrader.TraderCostPercepTime)

UNITS: Rs/kg

TotalMarketASupply = TotalRetailSupply+DistanceTrader.TotalDTSupply+LocalTrader.TotalLocalSupply

UNITS: kg

TotalRetailerFVExpenditure = RetailLOOPRevenue+RetailNonLOOPRevenue

UNITS: Rs

TotalRetailSupply = LOOPMarketable.MarketARetailLOOP+NonLOOPMarketable.MarketARetailNonLOOP

UNITS: kg

**Local Trader**

CumulativeLTCosts(t) = CumulativeLTCosts(t - dt) + (LTCostsBiflow) * dt

INIT CumulativeLTCosts = 1

UNITS: Rs

INFLOWS:

LTCostsBiflow = SMTH1(CostPerLT, 60)

UNITS: Rs/halfday

CumulativeLTRevenue(t) = CumulativeLTRevenue(t - dt) + (LRevenueBiflow) * dt

INIT CumulativeLTRevenue = 1

UNITS: Rs

INFLOWS:

LRevenueBiflow = RevenuePerLT

UNITS: Rs/halfday

LocalTraderStock(t) = LocalTraderStock(t - dt) + (MarketALocalNonLOOP + MarketALocalLOOP + ExcessDistanceTraderSupply - LocalTraderDownstreamFlow - ExcessLocalTraderSupply) * dt

INIT LocalTraderStock = MarketALTDemand

UNITS: kg

INFLOWS:

MarketALocalNonLOOP = IF LOOPMarketable.TotalLOOPAggregation >0 THEN NonLOOPMarketableF&V*NonLOOPMarketAProportion*TraderPreference.NonLOOPMorningLTSupplyPreferenceUSE ELSE NonLOOPMarketableF&V*NonLOOPMarketAProportion*TraderPreference.NonLOOPAfternoonLTSupplyPreferenceUSE

UNITS: kg/halfday

MarketALocalLOOP = PULSE(LOOPMarketableVolume*MarketPreference.MarketAPreferenceOverBUse*TraderPreference.LOOPMorningLTSupplyPreferenceUSE, 1, 2)

UNITS: kg/halfday

ExcessDistanceTraderSupply = IF TIME > 669 AND "A.ScenarioHub".ColdStorageSCENARIO >= 0.5 THEN (((1-DTAColdUtilityRate)*DistanceTraderSupplyStock)/4)+((DTAColdUtilityRate*DistanceTraderSupplyStock)/LocalRetailer.ColdStorageDuration) ELSE DistanceTraderSupplyStock/4

UNITS: kg/halfday

OUTFLOWS:

LocalTraderDownstreamFlow = MarketALTDemand*(1-LocalTraderHandlingWastageRate)

UNITS: kg/halfday

ExcessLocalTraderSupply = IF TIME > 669 AND "A.ScenarioHub".ColdStorageSCENARIO >= 0.5 THEN (((1-LTAColdUtilityRate)*LocalTraderStock)/4)+((LTAColdUtilityRate*LocalTraderStock)/LocalRetailer.ColdStorageDuration) ELSE LocalTraderStock/4

UNITS: kg/halfday

ColdStorageRentPerLTA = IF LTHalfDay = 0 THEN 0 ELSE LTAColdStorageRent/LTHalfDay

UNITS: Rs

CommissionPerLT = IF LTHalfDay = 0 THEN 0 ELSE GaddidarCommission.LTCommissionIncurred/LTHalfDay

UNITS: Rs

CostPerLT = LTOtherCostsPerLT+CommissionPerLT+VehicleCostsPerLT+FVExpenditurePerLT+ColdStorageRentPerLTA

UNITS: Rs

EffectofExpectedProfitDesiredCap = GRAPH(SMTH1(InvesmentExpectedProfitability, LTProfitPerceptionTime)) (-1.000, 0.000), (-0.500, 0.300), (0.000, 1.000), (0.500, 1.500), (1.000, 1.600)

UNITS: Dimensionless

ExpectedLongRunCosts = CumulativeLTCosts-DELAY(CumulativeLTCosts, 1)

UNITS: Rs

ExpectedLongRunRevenue = CumulativeLTRevenue- DELAY(CumulativeLTRevenue, 1)

UNITS: Rs

FVExpenditurePerLT = IF LTHalfDay = 0 THEN 0 ELSE (TotalLTFVExpenditure)/LTHalfDay

UNITS: Rs

InvesmentExpectedProfitability = IF ExpectedLongRunCosts = 0 THEN 1 ELSE (ExpectedLongRunRevenue-ExpectedLongRunCosts)/ExpectedLongRunCosts

UNITS: Rs

LocalCoveragePerceptionTime = 14

UNITS: halfday

LocalEffectCostsOnPrice = IF DistanceTrader.QualityAdjDTBuyingPrice = 0 THEN 1+LocalSensiPriceToCosts*((SmthLTExpCosts/DELAY(DistanceTrader.QualityAdjDTBuyingPrice, 1)-1)) ELSE 1+LocalSensiPriceToCosts*((SmthLTExpCosts/DistanceTrader.QualityAdjDTBuyingPrice)-1)

UNITS: Dimensionless

LocalEffectInventoryCoveragePrice = PerceivedLocalInventoryCoverage^LocalSensiPriceInventoryCoverage

UNITS: Dimensionless

LocalExpectedCosts = IF LocalTraderDownstreamFlow = 0 THEN 0 ELSE ((VehicleCostsPerLT+LTOtherCostsPerLT+CommissionPerLT+ColdStorageRentPerLTA)*LTHalfDay)/LocalTraderDownstreamFlow

UNITS: Rs/kg

LocalInventoryCoverage = IF LocalTraderDownstreamFlow = 0 THEN 1 ELSE LocalTraderStock/LocalTraderDownstreamFlow

UNITS: Dimensionless

LocalSensiPriceInventoryCoverage = -0.3

UNITS: Dimensionless

LocalSensiPriceToCosts = -0.1

UNITS: Dimensionless

LocalTraderBuyingPrice = LocalTraderExpectedPrice*LocalEffectCostsOnPrice*LocalEffectInventoryCoveragePrice

UNITS: Rs/kg

LocalTraderCapacity = 600

UNITS: kg/halfday

LocalTraderExpectedPrice = (DistanceTrader.QualityAdjDTBuyingPrice)

UNITS: Rs/kg

LocalTraderHandlingWastageRate = 0.05

UNITS: Dimensionless

LongRunProfits = SMTH1(ProfitPerLT - DELAY(ProfitPerLT, LTProfitPerceptionTime), LTProfitPerceptionTime)

UNITS: Rs

LOOPFarmersSupplyingLT = IF LOOPMarketable.MorningIndicator = 1 THEN (LOOPMarketable.LOOPMarketAFarmers*TraderPreference.NonLOOPMorningLTSupplyPreferenceUSE)+DistanceTrader.LOOPDTFarmerExcess - LOOPLTFarmerExcess ELSE 0

UNITS: People

LOOPLTExcess = IF ProportionLOOPLTSupply*ExcessLocalTraderSupply > DELAY(LOOPMarketable.MarketALocalLOOP, 1) THEN LOOPMarketable.MarketALocalLOOP ELSE ProportionLOOPLTSupply*ExcessLocalTraderSupply

UNITS: kg

LOOPLTFarmerExcess = IF LOOPMarketable.LOOPMarketAPerFarmer = 0 THEN 0 ELSE LOOPLTExcess/LOOPMarketable.LOOPMarketAPerFarmer

UNITS: People

LTAColdStorageRent = LTAColdUtilityRate*LocalTraderStock*MarketBLocalRetailer.TraderColdStorageRentRate

UNITS: Rs

LTAColdUtilityRate = IF LTAStockAccomodated < 1 THEN LTAStockAccomodated ELSE 1

UNITS: Dimensionless

LTAStockAccomodated = IF LocalTraderStock = 0 THEN 0 ELSE (LocalRetailer.MarketAColdStorageCapacity*LTColdStorageProp)/LocalTraderStock

UNITS: Dimensionless

LTColdStorageProp = IF LocalRetailer.TotalMarketASupply = 0 THEN 0 ELSE TotalLocalSupply/LocalRetailer.TotalMarketASupply

UNITS: Dimensionless

LTDay = EffectofExpectedProfitDesiredCap*LTSeasonal

UNITS: People

LTFrequency = 2

UNITS: halfday

LTHalfDay = LTDay/LTFrequency

UNITS: People

LTLOOPRevenue = ProportionLOOPLTSupply*LocalTraderDownstreamFlow*QualityAdjLTBuyingPrice

UNITS: Rs

LTLOOPRevenuePerFarmer = IF LOOPFarmersSupplyingLT = 0 THEN 0 ELSE LTLOOPRevenue/LOOPFarmersSupplyingLT

UNITS: Rs

LTLOOPSold = IF LocalTraderDownstreamFlow*ProportionLOOPLTSupply > DELAY(LOOPMarketable.MarketALocalLOOP, 2) + LOOPMarketable.MarketALocalLOOP THEN DELAY(LOOPMarketable.MarketALocalLOOP, 2) + LOOPMarketable.MarketALocalLOOP ELSE LocalTraderDownstreamFlow*ProportionLOOPLTSupply

UNITS: kg

LTMarginGraphical = GRAPH(PerceivedLocalInventoryCoverage) (0.000, 8.000), (1.000, 0.000)

UNITS: Rs/kg

LTNonLOOPRevenue = QualityAdjLTBuyingPrice*(1-ProportionLOOPLTSupply)*LocalTraderDownstreamFlow

UNITS: Rs

LTNonLOOPRevenuePerFarmer = IF NonLOOPFarmersSupplyingLT = 0 THEN 0 ELSE LTNonLOOPRevenue/NonLOOPFarmersSupplyingLT

UNITS: Rs

LTNonLOOPSold = LocalTraderDownstreamFlow-LTLOOPSold

UNITS: kg

LTOtherCostsPerLT = NORMAL(10, 2.5, 43)

UNITS: Rs

LTProfitPerceptionTime = 60

UNITS: halfday

LTRevenue = (LocalTraderDownstreamFlow*LTSellingPrice)*(1-WastageRateEnRoute)

UNITS: Rs

LTSeasonal = GRAPH(TIME) (1, 20.0), (184, 30.0), (364, 30.0), (730, 25.0), (914, 30.0), (1094, 30.0), (1460, 25.0), (1644, 30.0), (1824, 30.0), (2190, 25.0), (2374, 30.0), (2554, 30.0), (2920, 25.0), (3104, 30.0)

UNITS: People

LTSellingPrice = LTMarginGraphical+QualityAdjLTBuyingPrice

UNITS: Rs/kg

MarketALTDemand = LTHalfDay*LocalTraderCapacity

UNITS: kg

NonLOOPFarmersSupplyingLT = IF LOOPMarketable.MorningIndicator = 1 THEN (NonLOOPMarketable.NonLOOPFarmersMarketA*TraderPreference.NonLOOPMorningLTSupplyPreferenceUSE)+DistanceTrader.NonLOOPDTFarmerExcess - NonLOOPLTFarmerExcess ELSE (NonLOOPMarketable.NonLOOPFarmersMarketA*TraderPreference.NonLOOPAfternoonLTSupplyPreferenceUSE)+DistanceTrader.NonLOOPDTFarmerExcess - NonLOOPLTFarmerExcess

UNITS: People

NonLOOPLTExcess = ExcessLocalTraderSupply-LOOPLTExcess

UNITS: kg

NonLOOPLTFarmerExcess = IF NonLOOPMarketable.NonLOOPMarketAPerFarmer= 0 THEN 0 ELSE NonLOOPLTExcess/NonLOOPMarketable.NonLOOPMarketAPerFarmer

UNITS: People

PerceivedLocalInventoryCoverage = SMTH1(LocalInventoryCoverage, LocalCoveragePerceptionTime)

UNITS: Dimensionless

ProfitPerLT = RevenuePerLT-CostPerLT

UNITS: Rs

ProportionLOOPLTSupply = IF LOOPMarketable.MarketALocalLOOP = 0 OR DistanceTrader.LOOPDTExcess = 0 THEN 0 ELSE (LOOPMarketable.MarketALocalLOOP+DistanceTrader.LOOPDTExcess)/(LOOPMarketable.MarketALocalLOOP+DistanceTrader.LOOPDTExcess+NonLOOPMarketable.MarketALocalNonLOOP+DistanceTrader.NonLOOPDTExcess)

UNITS: Dimensionless

QualityAdjLTBuyingPrice = (LocalTraderBuyingPrice*(1-ProportionLOOPLTSupply)*NonLOOPLand&Yield.SmoothNonLOOPSupplyQuality) + ((LocalTraderBuyingPrice*DistanceTrader.QualityPriceDifference)*(1-ProportionLOOPLTSupply)*(1-NonLOOPLand&Yield.SmoothNonLOOPSupplyQuality)) + (LocalTraderBuyingPrice*ProportionLOOPLTSupply*LOOPLand&Yield.SmoothLOOPSupplyQuality) + ((LocalTraderBuyingPrice*DistanceTrader.QualityPriceDifference)* ProportionLOOPLTSupply*(1-LOOPLand&Yield.SmoothLOOPSupplyQuality))

UNITS: Rs

RevenuePerLT = IF LTHalfDay = 0 THEN 0 ELSE LTRevenue/LTHalfDay

UNITS: Rs

SmthLTExpCosts = SMTH1(LocalExpectedCosts, DistanceTrader.TraderCostPercepTime)

UNITS: Rs/kg

TotalLocalSupply = LOOPMarketable.MarketALocalLOOP+NonLOOPMarketable.MarketALocalNonLOOP

UNITS: kg

TotalLTFVExpenditure = LTLOOPRevenue+LTNonLOOPRevenue

UNITS: Rs

VehicleCostsPerLT = NORMAL(100, 25, 44)

UNITS: Rs

WastageRateEnRoute = RANDOM(0.05, 0.10, 40)

UNITS: Dimensionless

***Trader Preference***

LocalTraderSupplyPreference(t) = LocalTraderSupplyPreference(t - dt) + (LTPreferenceIncrease + NonLOOPLTTrustChange) * dt

INIT LocalTraderSupplyPreference = 0.9

UNITS: Dimensionless

INFLOWS:

LTPreferenceIncrease = SMTH1((LocalTrader.QualityAdjLTBuyingPrice/(LocalTrader.QualityAdjLTBuyingPrice+LocalRetailer.QualityAdjRetBuyingPrice))-0.5, PricePerceptionTime)

UNITS: Dimensionless/halfday

NonLOOPLTTrustChange = IF SMTH1(DELAY(LocalTrader.NonLOOPLTFarmerExcess, TrustPerceptionTime)-LocalTrader.NonLOOPLTFarmerExcess, TrustPerceptionTime) = 0 THEN 0 ELSE ProtoNonLOOPLTTrustChange

UNITS: Dimensionless/halfday

LOOPDistanceTraderSupplyPreference(t) = LOOPDistanceTraderSupplyPreference(t - dt) + (LOOPDTPreferenceChange + LOOPDTTrustChange) * dt

INIT LOOPDistanceTraderSupplyPreference = 1

UNITS: Dimensionless

INFLOWS:

LOOPDTPreferenceChange = SMTH1((DistanceTrader.QualityAdjDTBuyingPrice/(DistanceTrader.QualityAdjDTBuyingPrice+MaxLocalWalaPrice))-0.5, PricePerceptionTime)

UNITS: Dimensionless/halfday

LOOPDTTrustChange = IF SMTH1(DELAY(DistanceTrader.LOOPDTFarmerExcess, TrustPerceptionTime)-DistanceTrader.LOOPDTFarmerExcess, TrustPerceptionTime) = 0 THEN 0 ELSE ProtoLOOPDTTrustChange

UNITS: Dimensionless/halfday

LOOPLTSupplyPreference(t) = LOOPLTSupplyPreference(t - dt) + (LOOPLTPreferenceIncrease + LOOPLTTrustChange) * dt

INIT LOOPLTSupplyPreference = 0.9

UNITS: Dimensionless

INFLOWS:

LOOPLTPreferenceIncrease = SMTH1((LocalTrader.QualityAdjLTBuyingPrice/(LocalTrader.QualityAdjLTBuyingPrice+LocalRetailer.QualityAdjRetBuyingPrice))-0.5, PricePerceptionTime)

UNITS: Dimensionless/halfday

LOOPLTTrustChange = IF SMTH1(DELAY(LocalTrader.LOOPLTFarmerExcess, TrustPerceptionTime)-LocalTrader.LOOPLTFarmerExcess, TrustPerceptionTime) = 0 THEN 0 ELSE ProtoLOOPLTTrustChange

UNITS: Dimensionless/halfday

NonLOOPDTSupplyPreference(t) = NonLOOPDTSupplyPreference(t - dt) + (NonLOOPDTPreferenceIncrease + NonLOOPDTTrustChange) * dt

INIT NonLOOPDTSupplyPreference = 0.9

UNITS: Dimensionless

INFLOWS:

NonLOOPDTPreferenceIncrease = SMTH1((DistanceTrader.QualityAdjDTBuyingPrice/(DistanceTrader.QualityAdjDTBuyingPrice+MaxLocalWalaPrice))-0.5, PricePerceptionTime)

UNITS: Dimensionless/halfday

NonLOOPDTTrustChange = IF SMTH1(DELAY(DistanceTrader.NonLOOPDTFarmerExcess, TrustPerceptionTime)-DistanceTrader.NonLOOPDTFarmerExcess, TrustPerceptionTime) = 0 THEN 0 ELSE ProtoNonLOOPDTTrustChange

UNITS: Dimensionless/halfday

LOOPCombinedPrefs = LOOPMorningLTSupplyPreferenceUSE+LOOPMorningRetailerSupplyPrefUSE+LOOPDistanceTraderSupplyPreferenceUSE

UNITS: Dimensionless

LOOPDistanceTraderSupplyPreferenceUSE = IF LOOPDistanceTraderSupplyPreference > 1 THEN 1 ELSE LOOPDistanceTraderSupplyPreference

UNITS: Dimensionless

LOOPMorningLTSupplyPreferenceUSE = IF LOOPMorningLTSupplyPrefProto > 1 THEN 1 ELSE LOOPMorningLTSupplyPrefProto

UNITS: Dimensionless

LOOPMorningLTSupplyPrefProto = (1-LOOPDistanceTraderSupplyPreferenceUSE)*LOOPLTSupplyPreference

UNITS: Dimensionless

LOOPMorningRetailerSupplyPrefUSE = IF 1-LOOPMorningLTSupplyPreferenceUSE-LOOPDistanceTraderSupplyPreferenceUSE < 0 THEN 0 ELSE 1-LOOPMorningLTSupplyPreferenceUSE-LOOPDistanceTraderSupplyPreferenceUSE

UNITS: Dimensionless

MaxLocalWalaPrice = MAX(LocalTrader.QualityAdjLTBuyingPrice, LocalRetailer.QualityAdjRetBuyingPrice)

UNITS: Rs

NonLOOPAfternoonLTSupplyPreferenceUSE = IF LocalTraderSupplyPreference <= 0.9 THEN LocalTraderSupplyPreference ELSE 0.9

UNITS: Dimensionless

NonLOOPAfternoonRetailerSupplyPreferenceUSE = 1-NonLOOPAfternoonLTSupplyPreferenceUSE

UNITS: Dimensionless

NonLOOPDTSupplyPreferenceProto = IF NonLOOPDTSupplyPreference < 0.1 THEN 0.1 ELSE NonLOOPDTSupplyPreference

UNITS: Dimensionless

NonLOOPDTSupplyPreferenceUSE = IF NonLOOPDTSupplyPreferenceProto > 0.9 THEN 0.9 ELSE NonLOOPDTSupplyPreferenceProto

UNITS: Dimensionless

NonLOOPMorningLTSupplyPreferenceUSE = (1-NonLOOPDTSupplyPreferenceUSE)*ProtoNLLTMorningPref

UNITS: Dimensionless

NonLOOPMorningRetailerPreferenceUSE = 1-NonLOOPMorningLTSupplyPreferenceUSE-NonLOOPDTSupplyPreferenceUSE

UNITS: Dimensionless

PricePerceptionTime = 14

UNITS: halfday

ProtoLOOPDTTrustChange = IF DistanceTrader.LOOPDTFarmerExcess= 0 THEN 0 ELSE SMTH1(DistanceTrader.LOOPDTFarmerExcess/(DistanceTrader.LOOPDTFarmerExcess+DELAY(DistanceTrader.LOOPDTFarmerExcess, TrustPerceptionTime)-0.5), TrustPerceptionTime)

UNITS: Dimensionless

ProtoLOOPLTTrustChange = IF LocalTrader.LOOPLTFarmerExcess=0 THEN 0 ELSE SMTH1(LocalTrader.LOOPLTFarmerExcess/(LocalTrader.LOOPLTFarmerExcess+DELAY(LocalTrader.LOOPLTFarmerExcess, TrustPerceptionTime)-0.5), TrustPerceptionTime)

UNITS: Dimensionless

ProtoNLLTMorningPref = IF LocalTraderSupplyPreference > 1 THEN 1 ELSE LocalTraderSupplyPreference

UNITS: Dimensionless

ProtoNonLOOPDTTrustChange = IF DistanceTrader.NonLOOPDTFarmerExcess= 0 THEN 0 ELSE SMTH1(DistanceTrader.NonLOOPDTFarmerExcess/(DistanceTrader.NonLOOPDTFarmerExcess+DELAY(DistanceTrader.NonLOOPDTFarmerExcess, TrustPerceptionTime)-0.5), TrustPerceptionTime)

UNITS: Dimensionless

ProtoNonLOOPLTTrustChange = IF LocalTrader.NonLOOPLTFarmerExcess=0 THEN 0 ELSE SMTH1(LocalTrader.NonLOOPLTFarmerExcess/(LocalTrader.NonLOOPLTFarmerExcess+DELAY(LocalTrader.NonLOOPLTFarmerExcess, TrustPerceptionTime)-0.5), TrustPerceptionTime)

UNITS: Dimensionless

TrustPerceptionTime = 60

UNITS: halfday

Market B

**Market B Gaddidar commission**

MarketBGaddidarLocalActorCommission = 0.05

UNITS: Rs/kg

MarketBLTFarmerCommissionIncurred = MarketBGaddidarLocalActorCommission*MarketBLocalTrader.MarketBLTDownstreamFlows

UNITS: Rs

MarketBLTLOOPCommissionIncurred = MarketBLocalTrader.MarketBLTProportionLOOP*MarketBLTFarmerCommissionIncurred

UNITS: Rs

MarketBLTNonLOOPCommissionIncurred = MarketBLTFarmerCommissionIncurred - MarketBLTLOOPCommissionIncurred

UNITS: Rs

MarketBLTTraderCommissionIncurred = MarketBGaddidarLocalActorCommission*MarketBLocalTrader.MarketBLTDownstreamFlows

UNITS: Rs

MarketBMeanWeightedPrice = IF TotalSalesMarketB = 0 THEN 0 ELSE (MarketBLocalTrader.QualityAdjLTBBuyingPrice*(MarketBLocalTrader.MarketBLTDownstreamFlows/TotalSalesMarketB))+(MarketBLocalRetailer.QualityAdjRetBBuyingPrice*(MarketBLocalRetailer.MarketBRetailerDownstreamFlow/TotalSalesMarketB))

UNITS: Rs/kg

MarketBRetailerCommissionIncurred = MarketBLocalRetailer.MarketBRetailerDownstreamFlow* MarketBGaddidarLocalActorCommission

UNITS: Rs

MarketBRetailerFarmerCommissionIncurred = MarketBGaddidarLocalActorCommission* MarketBLocalRetailer.MarketBRetailerDownstreamFlow

UNITS: Rs

MarketBRetailerLOOPCommissionIncurred = MarketBLocalRetailer.MarketBLOOPRetailerProportion* MarketBRetailerFarmerCommissionIncurred

UNITS: Rs

MarketBRetailerNonLOOPCommissionIncurred = MarketBRetailerFarmerCommissionIncurred-MarketBRetailerLOOPCommissionIncurred

UNITS: Rs

TotalSalesMarketB = MarketBLocalRetailer.MarketBRetailerDownstreamFlow+ MarketBLocalTrader.MarketBLTDownstreamFlows

UNITS: kg

Market B Local Retailer

CumulativeRetCosts(t) = CumulativeRetCosts(t - dt) + (RetCostsBiflow) * dt

INIT CumulativeRetCosts = 1

UNITS: Rs

INFLOWS:

RetCostsBiflow = SMTH1(CostPerRet, 60)

UNITS: Rs/halfday

CumulativeRetRevenue(t) = CumulativeRetRevenue(t - dt) + (RetRevenueBiflow) * dt

INIT CumulativeRetRevenue = 100

UNITS: Rs

INFLOWS:

RetRevenueBiflow = RevenuePerRet

UNITS: Rs/halfday

MarketBRetailerStock(t) = MarketBRetailerStock(t - dt) + (MarketBRetailLOOP + MarketBRetailLOOP + ExcessLTSupply - MarketBRetailerDownstreamFlow - RetailerUnsold) * dt

INIT MarketBRetailerStock = MarketBRetailerDemand

UNITS: kg

INFLOWS:

MarketBRetailLOOP = NonLOOPMarketableF&V*NonLOOPMarketBProportion*(1-MarketBWhichTrader.NonLOOPMarketBLTPreferenceUSE)

UNITS: kg/halfday

MarketBRetailLOOP = PULSE(LOOPMarketableVolume*(1-MarketPreference.MarketAPreferenceOverBUse)*(1-MarketBWhichTrader.NonLOOPMarketBLTPreferenceUSE), 1, 2)

UNITS: kg/halfday

ExcessLTSupply = IF TIME > 669 OR "A.ScenarioHub".ColdStorageSCENARIO >= 0.5 THEN (((1-LTBUtilityRate)*MarketBLTStock)/4)+((LTBUtilityRate*MarketBLTStock)/LocalRetailer.ColdStorageDuration) ELSE MarketBLTStock/4

UNITS: kg/halfday

OUTFLOWS:

MarketBRetailerDownstreamFlow = MarketBRetailerDemand

UNITS: kg/halfday

RetailerUnsold = IF TIME > 669 OR "A.ScenarioHub".ColdStorageSCENARIO >= 0.5 THEN (((1-RetBUtilityRate)*MarketBRetailerStock)/4)+((RetBUtilityRate*MarketBRetailerStock)/LocalRetailer.ColdStorageDuration) ELSE MarketBRetailerStock/4

UNITS: kg/halfday

AnnualCostOneColdUnit = 11.7E5

UNITS: Rs

ColdStorageRentPerRetB = IF RetailersHalfDay = 0 THEN 0 ELSE RetBColdStorageRent/RetailersHalfDay

UNITS: Rs

ColdStorageUnitCapacity = 5000

UNITS: kg

CommissionPerRetailer = IF RetailersHalfDay = 0 THEN 0 ELSE MarketBGaddidarCommission.MarketBRetailerCommissionIncurred/RetailersHalfDay

UNITS: Rs

CostPerRet = RetOtherCostsPerRet+CommissionPerRetailer+FVExpenditurePerRet+ColdStorageRentPerRetB

UNITS: Rs

EffectofExpectedProfitDesiredCap = GRAPH(SMTH1(InvesmentExpectedProfitability,RetProfitPerceptionTime)) (-1.000, 0.000), (-0.500, 0.300), (0.000, 1.000), (0.500, 1.500), (1.000, 1.600)

UNITS: Dimensionless

ExpectedLongRunCosts = CumulativeRetCosts-DELAY(CumulativeRetCosts, 1)

UNITS: Rs

ExpectedLongRunRevenue = CumulativeRetRevenue- DELAY(CumulativeRetRevenue, 1)

UNITS: Rs

Frequency = 1

UNITS: halfday

FVExpenditurePerRet = IF RetailersHalfDay = 0 THEN 0 ELSE (MarketBRetailNonLOOPRevenue+ MarketBRetailLOOPRevenue)/RetailersHalfDay

UNITS: Rs

GovtRentRecouped = MarketBLocalTrader.LTBColdStorageRent+ColdStorageRentPerRetB+LocalRetailer.RetAColdStorageRent+DistanceTrader.DTColdStorageRent+LocalTrader.LTAColdStorageRent

UNITS: Rs

InvesmentExpectedProfitability = IF ExpectedLongRunCosts = 0 OR ExpectedLongRunRevenue =0 THEN 1 ELSE (ExpectedLongRunRevenue-ExpectedLongRunCosts)/ExpectedLongRunCosts

UNITS: Rs

LongRunProfits = SMTH1(ProfitPerRet - DELAY(ProfitPerRet, RetProfitPerceptionTime), RetProfitPerceptionTime)

UNITS: Rs

LOOPLTFarmerExcess = IF LOOPMarketable.LOOPMarketBPerFarmer= 0 THEN 0 ELSE LOOPRetailerExcess/LOOPMarketable.LOOPMarketBPerFarmer

UNITS: People

LOOPRetailerExcess = IF MarketBLOOPRetailerProportion*RetailerUnsold > DELAY(LOOPMarketable.MarketBRetailLOOP, 1) THEN LOOPMarketable.MarketBRetailLOOP ELSE MarketBLOOPRetailerProportion*RetailerUnsold

UNITS: kg

MarketBLOOPRetailerProportion = IF LOOPMarketable.MarketBRetailLOOP+ NonLOOPMarketable.MarketBRetailLOOP= 0 THEN 0 ELSE (LOOPMarketable.MarketBRetailLOOP+MarketBLocalTrader.MarketBLTLOOPExcess)/(LOOPMarketable.MarketBRetailLOOP+NonLOOPMarketable.MarketBRetailLOOP+MarketBLocalTrader.MarketBLTLOOPExcess+MarketBLocalTrader.MarketBLTNonLOOPExcess)

UNITS: Dimensionless

MarketBLOOPRetailerSold = IF MarketBRetailerDownstreamFlow*MarketBLOOPRetailerProportion > DELAY(LOOPMarketable.MarketBRetailLOOP, 2) + LOOPMarketable.MarketBRetailLOOP THEN DELAY(LOOPMarketable.MarketBRetailLOOP, 2) + LOOPMarketable.MarketBRetailLOOP ELSE MarketBLOOPRetailerProportion*MarketBRetailerDownstreamFlow

UNITS: kg

MarketBNonLOOPFarmersSupplyingRetail = ((1-MarketBWhichTrader.NonLOOPMarketBLTPreferenceUSE)* NonLOOPMarketable.NonLOOPFarmersMarketB) + MarketBLocalTrader.NonLOOPFarmersLTExcess-NonLOOPLTFarmerExcess

UNITS: People

MarketBNonLOOPRetailRevenuePerFarmer = IF MarketBNonLOOPFarmersSupplyingRetail = 0 THEN 0 ELSE MarketBRetailNonLOOPRevenue / MarketBNonLOOPFarmersSupplyingRetail

UNITS: Rs

MarketBRetailerDemand = MarketBDemand.MarketBPerRetailerDemands*RetailersHalfDay

UNITS: kg

MarketBRetailerBuyingPrice = MarketBRetailerExpectedPrice* MarketBRetailerEffectCostsOnPrice* RetailerEffectInventoryCoveragePrice

UNITS: Rs/kg

MarketBRetailerCoveragePerceptionTime = 14

UNITS: halfday

MarketBRetailerEffectCostsOnPrice = 1+MarketBRetailerSensiPriceToCosts* ((SmthRetExpCosts/LocalTrader.LTSellingPrice)-1)

UNITS: Dimensionless

MarketBRetailerExpectedPrice = LocalTrader.LTSellingPrice

UNITS: Rs/kg

MarketBRetailerInventoryCoverage = IF MarketBRetailerDownstreamFlow = 0 THEN 1 ELSE MarketBRetailerStock/MarketBRetailerDownstreamFlow

UNITS: Dimensionless

MarketBRetailerMargin = GRAPH(MarketBRetailerPerceivedInventoryCoverage) (0.000, 8.000), (1.000, 0.000)

UNITS: Rs/kg

MarketBRetailerPerceivedInventoryCoverage = SMTH1(MarketBRetailerInventoryCoverage, MarketBRetailerCoveragePerceptionTime)

UNITS: Dimensionless

MarketBRetailerSellingPrice = QualityAdjRetBBuyingPrice+MarketBRetailerMargin

UNITS: Rs/kg

MarketBRetailerSensiPriceToCosts = -0.1

UNITS: Dimensionless

MarketBRetailLOOPFarmersSupplying = IF LOOPMarketable.MorningIndicator = 1 THEN ((1-MarketBWhichTrader.LOOPMarketBLTPreferenceUSE)*LOOPMarketable.LOOPMarketBFarmers)+MarketBLocalTrader.LOOPFarmersLTExcess-LOOPLTFarmerExcess ELSE 0

UNITS: People

MarketBRetailLOOPRevenue = MarketBRetailerDownstreamFlow* MarketBLOOPRetailerProportion* MarketBRetailerBuyingPrice

UNITS: Rs

MarketBRetailLOOPRevenuePerFarmer = IF MarketBRetailLOOPFarmersSupplying = 0 THEN 0 ELSE MarketBRetailLOOPRevenue/MarketBRetailLOOPFarmersSupplying

UNITS: Rs

MarketBRetailNonLOOPRevenue = MarketBRetailerDownstreamFlow*(1-MarketBLOOPRetailerProportion)*MarketBRetailerBuyingPrice

UNITS: Rs

MarktBNonLOOPRetailerSold = MarketBRetailerDownstreamFlow-MarketBLOOPRetailerSold

UNITS: kg

NonLOOPLTFarmerExcess = IF NonLOOPMarketable.NonLOOPMarketBPerFarmer= 0 THEN 0 ELSE NonLOOPRetailerExcess/NonLOOPMarketable.NonLOOPMarketBPerFarmer

UNITS: People

NonLOOPRetailerExcess = RetailerUnsold-LOOPRetailerExcess

UNITS: kg

ProfitPerRet = RevenuePerRet-CostPerRet

UNITS: Rs

QualityAdjRetBBuyingPrice = (MarketBRetailerBuyingPrice*(1-MarketBLOOPRetailerProportion)* NonLOOPLand&Yield.SmoothNonLOOPSupplyQuality) + ((MarketBRetailerBuyingPrice* MarketBLocalTrader.MarketBQualityPriceDifference)*(1-MarketBLOOPRetailerProportion)* (1-NonLOOPLand&Yield.SmoothNonLOOPSupplyQuality)) + (MarketBRetailerBuyingPrice *MarketBLOOPRetailerProportion*LOOPLand&Yield.SmoothLOOPSupplyQuality) + ((MarketBRetailerBuyingPrice*MarketBLocalTrader.MarketBQualityPriceDifference) *MarketBLOOPRetailerProportion*(1-LOOPLand&Yield.SmoothLOOPSupplyQuality))

UNITS: Rs/kg

RetailBGovtColdStorageCostHalfDay = ("A.ScenarioHub".ColdStorageInvestmentLevelSCENARIO* AnnualCostOneColdUnit)/730

UNITS: Rs

RetailerEffectInventoryCoveragePrice = MarketBRetailerPerceivedInventoryCoverage^ RetailerSensiPriceInventoryCoverage

UNITS: Dimensionless

RetailerExpCosts = IF MarketBRetailerDownstreamFlow = 0 THEN 0 ELSE ((RetOtherCostsPerRet+CommissionPerRetailer)*RetailersHalfDay+ColdStorageRentPerRetB)/MarketBRetailerDownstreamFlow

UNITS: Rs/kg

RetailerSensiPriceInventoryCoverage = -0.3

UNITS: Dimensionless

RetailersHalfDay = TotalMarketBRetailers/Frequency

UNITS: People

RetailRevenueMarketB = MarketBRetailerSellingPrice*MarketBRetailerDownstreamFlow

UNITS: Rs

RetBColdProp = IF TotalMarketBSupply = 0 THEN 0 ELSE TotalRetailerSupply/TotalMarketBSupply

UNITS: Dimensionless

RetBColdStorageCapacity = IF "A.ScenarioHub".ColdStorageSCENARIO >= 0.5 THEN "A.ScenarioHub".ColdStorageInvestmentLevelSCENARIO*ColdStorageUnitCapacity ELSE 0

UNITS: kg

RetBColdStorageRent = MarketBRetailerStock*RetBUtilityRate*TraderColdStorageRentRate

UNITS: Rs

RetBStockAccomodated = IF MarketBRetailerStock = 0 THEN 0 ELSE (RetBColdStorageCapacity*RetBColdProp)/MarketBRetailerStock

UNITS: Dimensionless

RetBUtilityRate = IF RetBStockAccomodated < 1 THEN RetBStockAccomodated ELSE 1

UNITS: Dimensionless

RetOtherCostsPerRet = NORMAL(10, 2.5, 45)

UNITS: Rs

RetProfitPerceptionTime = 240

UNITS: halfday

RevenuePerRet = IF RetailersHalfDay =0 THEN 0 ELSE RetailRevenueMarketB/RetailersHalfDay

UNITS: Rs

SmthRetExpCosts = SMTH1(RetailerExpCosts, DistanceTrader.TraderCostPercepTime)

UNITS: Rs/kg

TotalGovtColdStorageExpenditure = RetailBGovtColdStorageCostHalfDay+LocalRetailer.RetAGovtCostsHalfDay

UNITS: Rs

TotalMarketBRetailers = 10*MarketBLocalTrader.SmallMarkets*EffectofExpectedProfitDesiredCap

UNITS: People

TotalMarketBSupply = TotalRetailerSupply+MarketBLocalTrader.TotalMarketBLTSupply

UNITS: kg

TotalRetailerSupply = NonLOOPMarketable.MarketBRetailLOOP+LOOPMarketable.MarketBRetailLOOP

UNITS: kg

TraderColdStorageRentRate = 0.15

UNITS: Rs/kg/halfday

***Market B Local Trader***

CumulativeLTCosts(t) = CumulativeLTCosts(t - dt) + (LTCostsBiflow) * dt

INIT CumulativeLTCosts = 1

UNITS: Rs

INFLOWS:

LTCostsBiflow = SMTH1(CostPerLT, 60)

UNITS: Rs/halfday

CumulativeLTRevenue(t) = CumulativeLTRevenue(t - dt) + (LRevenueBiflow) * dt

INIT CumulativeLTRevenue = 1

UNITS: Rs

INFLOWS:

LRevenueBiflow = RevenuePerLT

UNITS: Rs/halfday

MarketBLTStock(t) = MarketBLTStock(t - dt) + (MarketBLocalNonLOOP + MarketBLocalLOOP - MarketBLTDownstreamFlows - ExcessLTSupply) * dt

INIT MarketBLTStock = MarketBLocalTraderDemand

UNITS: kg

INFLOWS:

MarketBLocalNonLOOP = NonLOOPMarketBProportion* MarketBWhichTrader.NonLOOPMarketBLTPreferenceUSE*NonLOOPMarketableF&V

UNITS: kg/halfday

MarketBLocalLOOP = PULSE(LOOPMarketableVolume*(1-MarketPreference.MarketAPreferenceOverBUse)*MarketBWhichTrader.NonLOOPMarketBLTPreferenceUSE, 1, 2)

UNITS: kg/halfday

OUTFLOWS:

MarketBLTDownstreamFlows = MarketBLocalTraderDemand*(1-LocalTraderWastageRate)

UNITS: kg/halfday

ExcessLTSupply = IF TIME > 669 OR "A.ScenarioHub".ColdStorageSCENARIO >= 0.5 THEN (((1-LTBUtilityRate)*MarketBLTStock)/4)+((LTBUtilityRate*MarketBLTStock)/LocalRetailer.ColdStorageDuration) ELSE MarketBLTStock/4

UNITS: kg/halfday

ColdStorageRentPerLTB = LTBColdStorageRent/HalfDayMarketBLT

UNITS: Dimensionless

CommissionPerLT = IF HalfDayMarketBLT = 0 THEN 0 ELSE MarketBGaddidarCommission.MarketBLTTraderCommissionIncurred/HalfDayMarketBLT

UNITS: Rs

CostPerLT = OtherCostsPerLT+CommissionPerLT+VehicleCostsPerLT+FVExpenditurePerLT+ColdStorageRentPerLTB

UNITS: Rs

EffectofExpectedProfitDesiredCap = GRAPH(SMTH1(InvesmentExpectedProfitability, LTProfitPerceptionTime)) (-1.000, 0.000), (-0.500, 0.300), (0.000, 1.000), (0.500, 1.500), (1.000, 1.600)

UNITS: Dimensionless

ExpectedCostsLT = IF MarketBLTDownstreamFlows = 0 THEN 0 ELSE ((CommissionPerLT+ OtherCostsPerLT+VehicleCostsPerLT+ColdStorageRentPerLTB)*HalfDayMarketBLT)/MarketBLTDownstreamFlows

UNITS: Rs/kg

ExpectedLongRunCosts = CumulativeLTCosts-DELAY(CumulativeLTCosts, 1)

UNITS: Rs

ExpectedLongRunRevenue = CumulativeLTRevenue- DELAY(CumulativeLTRevenue, 1)

UNITS: Rs

Frequency = 2

UNITS: halfday

FVExpenditurePerLT = IF HalfDayMarketBLT=0 THEN 0 ELSE (TotalLTFVExpenditure)/HalfDayMarketBLT

UNITS: Rs

HalfDayMarketBLT = TotalMarketBLT/Frequency

UNITS: People

InvesmentExpectedProfitability = IF ExpectedLongRunCosts = 0 THEN 1 ELSE (ExpectedLongRunRevenue-ExpectedLongRunCosts)/ExpectedLongRunCosts

UNITS: Rs

LocalEffectInventoryCoveragePrice = MarketBLTPerceivedInventoryCoverage^LocalSensiPriceInventoryCoverage

UNITS: Dimensionless

LocalSensiPriceInventoryCoverage = -0.3

UNITS: Dimensionless

LocalTraderWastageRate = 0.05

UNITS: Dimensionless

LongRunProfits = SMTH1(ProfitPerLT - DELAY(ProfitPerLT, LTProfitPerceptionTime), LTProfitPerceptionTime)

UNITS: Rs

LOOPFarmersLTExcess = IF LOOPMarketable.LOOPMarketBPerFarmer= 0 THEN 0 ELSE MarketBLTLOOPExcess/LOOPMarketable.LOOPMarketBPerFarmer

UNITS: People

LOOPFarmersSupplyingLT = IF LOOPMarketable.MorningIndicator = 1 THEN (LOOPMarketable.LOOPMarketBFarmers*MarketBWhichTrader.LOOPMarketBLTPreferenceUSE) - LOOPFarmersLTExcess ELSE 0

UNITS: People

LTBColdStorageRent = MarketBLTStock*LTBUtilityRate* MarketBLocalRetailer.TraderColdStorageRentRate

UNITS: Rs

LTBStockAccomodated = IF MarketBLTStock = 0 THEN 0 ELSE (MarketBLocalRetailer.RetBColdStorageCapacity*MarketBLTColdProp)/MarketBLTStock

UNITS: Dimensionless

LTBUtilityRate = IF LTBStockAccomodated < 1 THEN LTBStockAccomodated ELSE 1

UNITS: Dimensionless

LTLOOPRevenuePerFarmer = IF LOOPFarmersSupplyingLT = 0 THEN 0 ELSE MarketBLTLOOPRevenue/LOOPFarmersSupplyingLT

UNITS: Rs

LTNonLOOPRevenuePerFarmer = IF NonLOOPFarmersSupplyingLT = 0 THEN 0 ELSE MarketBLTNonLOOPRevenue/NonLOOPFarmersSupplyingLT

UNITS: Rs

LTProfitPerceptionTime = 60

UNITS: halfday

MarketBLocalTraderDemand = MarketBLTCapacity*HalfDayMarketBLT

UNITS: kg

MarketBLTBuyingPrice = MarketBLTExpectedPrice*MarketBLTEffectCostsOnPrice* LocalEffectInventoryCoveragePrice

UNITS: Rs/kg

MarketBLTCapacity = 300

UNITS: kg/halfday

MarketBLTColdProp = IF TotalMarketBLTSupply = 0 THEN 0 ELSE MarketBLocalRetailer.TotalMarketBSupply/TotalMarketBLTSupply

UNITS: Dimensionless

MarketBLTCoveragePerceptionTime = 14

UNITS: halfday

MarketBLTEffectCostsOnPrice = 1+MarketBLTSensiPriceToCosts*((SmthLTExpCosts/ DistanceTrader.QualityAdjDTBuyingPrice)-1)

UNITS: Dimensionless

MarketBLTExpectedPrice = (DistanceTrader.QualityAdjDTBuyingPrice)

UNITS: Rs/kg

MarketBLTInventoryCoverage = IF MarketBLTDownstreamFlows = 0 THEN 1 ELSE MarketBLTStock/(MarketBLTDownstreamFlows)

UNITS: Dimensionless

MarketBLTLOOPExcess = IF ExcessLTSupply*MarketBLTProportionLOOP > DELAY(LOOPMarketable.MarketBLocalLOOP, 1) THEN LOOPMarketable.MarketBLocalLOOP ELSE MarketBLTProportionLOOP*ExcessLTSupply

UNITS: kg

MarketBLTLOOPRevenue = MarketBLTProportionLOOP*MarketBLTDownstreamFlows* QualityAdjLTBBuyingPrice

UNITS: Rs

MarketBLTLOOPSold = IF MarketBLTDownstreamFlows*MarketBLTProportionLOOP > DELAY(LOOPMarketable.MarketBLocalLOOP, 2) + LOOPMarketable.MarketBLocalLOOP THEN DELAY(LOOPMarketable.MarketBLocalLOOP, 2) + LOOPMarketable.MarketBLocalLOOP ELSE MarketBLTDownstreamFlows*MarketBLTProportionLOOP

UNITS: kg

MarketBLTMargin = GRAPH(MarketBLTInventoryCoverage)

(0.000, 8.000), (1.000, 0.000)

UNITS: Rs/kg

MarketBLTNonLOOPExcess = ExcessLTSupply-MarketBLTLOOPExcess

UNITS: kg

MarketBLTNonLOOPRevenue = MarketBLTDownstreamFlows*(1-MarketBLTProportionLOOP)*QualityAdjLTBBuyingPrice

UNITS: Rs

MarketBLTPerceivedInventoryCoverage = SMTH1(MarketBLTInventoryCoverage, MarketBLTCoveragePerceptionTime)

UNITS: Dimensionless

MarketBLTProportionLOOP = IF LOOPMarketable.MarketBLocalLOOP+NonLOOPMarketable.MarketBLocalNonLOOP = 0 THEN 0 ELSE LOOPMarketable.MarketBLocalLOOP/(NonLOOPMarketable.MarketBLocalNonLOOP+LOOPMarketable.MarketBLocalLOOP)

UNITS: Dimensionless

MarketBLTRevenue = MarketBLTSellingPrice*MarketBLTDownstreamFlows*(1-WastageRateEnRoute)

UNITS: Rs

MarketBLTSellingPrice = MarketBLTMargin+QualityAdjLTBBuyingPrice

UNITS: Rs/kg

MarketBLTSensiPriceToCosts = -0.1

UNITS: Dimensionless

MarketBNonLOOPLTSold = MarketBLTDownstreamFlows-MarketBLTLOOPSold

UNITS: kg

MarketBQualityPriceDifference = 0.5

UNITS: Dimensionless

NonLOOPFarmersLTExcess = IF NonLOOPMarketable.NonLOOPMarketBPerFarmer= 0 THEN 0 ELSE MarketBLTNonLOOPExcess/NonLOOPMarketable.NonLOOPMarketBPerFarmer

UNITS: People

NonLOOPFarmersSupplyingLT = (NonLOOPMarketable.NonLOOPFarmersMarketB* MarketBWhichTrader.NonLOOPMarketBLTPreferenceUSE)-NonLOOPFarmersLTExcess

UNITS: People

OtherCostsPerLT = NORMAL(10, 2.5, 43)

UNITS: Rs

ProfitPerLT = RevenuePerLT-CostPerLT

UNITS: Rs

QualityAdjLTBBuyingPrice = (MarketBLTBuyingPrice*(1-MarketBLTProportionLOOP)* NonLOOPLand&Yield.SmoothNonLOOPSupplyQuality) + ((MarketBLTBuyingPrice* MarketBQualityPriceDifference)*(1-MarketBLTProportionLOOP)*(1-NonLOOPLand&Yield.SmoothNonLOOPSupplyQuality)) + (MarketBLTBuyingPrice* MarketBLTProportionLOOP*LOOPLand&Yield.SmoothLOOPSupplyQuality) + ((MarketBLTBuyingPrice*MarketBQualityPriceDifference)*MarketBLTProportionLOOP*(1-LOOPLand&Yield.SmoothLOOPSupplyQuality))

UNITS: Rs/kg

RevenuePerLT = IF HalfDayMarketBLT=0 THEN 0 ELSE MarketBLTRevenue/HalfDayMarketBLT

UNITS: Rs

SmallMarkets = 4

UNITS: Markets

SmthLTExpCosts = SMTH1(DistanceTrader.TraderCostPercepTime, ExpectedCostsLT)

UNITS: Rs/kg

TotalLTFVExpenditure = MarketBLTNonLOOPRevenue+MarketBLTLOOPRevenue

UNITS: Rs

TotalMarketBLT = SmallMarkets*2*EffectofExpectedProfitDesiredCap

UNITS: People

TotalMarketBLTSupply = LOOPMarketable.MarketBLocalLOOP+ NonLOOPMarketable.MarketBLocalNonLOOP

UNITS: kg

VehicleCostsPerLT = NORMAL(100, 25, 44)

UNITS: Rs

WastageRateEnRoute = RANDOM(0.05, 0.10, 40)

UNITS: Dimensionless

*Trader Preference*

LOOPMarketBLTOverRetailer(t) = LOOPMarketBLTOverRetailer(t - dt) + (LOOPLTPreferenceChange + LOOPMarketBLTTrustChange) * dt

INIT LOOPMarketBLTOverRetailer = 0.8

UNITS: Dimensionless

INFLOWS:

LOOPLTPreferenceChange = SMTH1((LocalTrader.QualityAdjLTBuyingPrice/(LocalTrader.QualityAdjLTBuyingPrice+MarketBLocalRetailer.QualityAdjRetBBuyingPrice))-0.5, PricePerceptionTime)

UNITS: Dimensionless/halfday

LOOPMarketBLTTrustChange = IF SMTH1(DELAY(MarketBLocalTrader.LOOPFarmersLTExcess, 60)-MarketBLocalTrader.LOOPFarmersLTExcess, MarketBTrustPerceptionTime) = 0 THEN 0 ELSE ProtoLOOPMarketBLTTrustChange

UNITS: Dimensionless/halfday

NonLOOPMarketBLTOverRetailer(t) = NonLOOPMarketBLTOverRetailer(t - dt) + (NonLOOPLTPreferenceChange + NonLOOPMarketBLTTrustChange) * dt

INIT NonLOOPMarketBLTOverRetailer = 0.8

UNITS: Dimensionless

INFLOWS:

NonLOOPLTPreferenceChange = SMTH1((LocalTrader.QualityAdjLTBuyingPrice/(LocalTrader.QualityAdjLTBuyingPrice+MarketBLocalRetailer.QualityAdjRetBBuyingPrice))-0.5, PricePerceptionTime)

UNITS: Dimensionless/halfday

NonLOOPMarketBLTTrustChange = IF SMTH1(DELAY(MarketBLocalTrader.NonLOOPFarmersLTExcess, 60)-MarketBLocalTrader.NonLOOPFarmersLTExcess, MarketBTrustPerceptionTime) = 0 THEN 0 ELSE ProtoNonLOOPMarketBLTTrustChange

UNITS: Dimensionless/halfday

LOOPMarketBLTPreferenceUSE = IF LOOPMarketBLTOverRetailer >0.9 THEN 0.9 ELSE LOOPMarketBLTOverRetailer

UNITS: Dimensionless

MarketBTrustPerceptionTime = 60

UNITS: halfday

NonLOOPMarketBLTPreference = IF NonLOOPMarketBLTOverRetailer < 0.3 THEN 0.3 ELSE NonLOOPMarketBLTOverRetailer

UNITS: Dimensionless

NonLOOPMarketBLTPreferenceUSE = IF NonLOOPMarketBLTPreference >0.9 THEN 0.9 ELSE NonLOOPMarketBLTPreference

UNITS: Dimensionless

PricePerceptionTime = 14

UNITS: halfday

ProtoLOOPMarketBLTTrustChange = IF MarketBLocalTrader.LOOPFarmersLTExcess= 0 THEN 0 ELSE SMTH1(MarketBLocalTrader.LOOPFarmersLTExcess/ (MarketBLocalTrader.LOOPFarmersLTExcess+DELAY(MarketBLocalTrader.LOOPFarmersLTExcess, MarketBTrustPerceptionTime)-0.5), MarketBTrustPerceptionTime)

UNITS: Dimensionless

ProtoNonLOOPMarketBLTTrustChange = IF MarketBLocalTrader.NonLOOPFarmersLTExcess= 0 THEN 0 ELSE SMTH1(MarketBLocalTrader.NonLOOPFarmersLTExcess/ (MarketBLocalTrader.NonLOOPFarmersLTExcess+DELAY(MarketBLocalTrader.NonLOOPFarmersLTExcess, MarketBTrustPerceptionTime)-0.5), MarketBTrustPerceptionTime)

UNITS: Dimensionless

F.5 NonLoop Benefits & Costs

Non-Loop Benefits Market A

AverageProfitPerNonLOOPFarmer = AverageRevenuePerNonLOOPFarmer-MarketACostsPerNonLOOPFarmer

UNITS: Rs

AverageRevenuePerNonLOOPFarmer = TotalNonLoopRevenueMarketA/ NonLOOPMarketable.NonLOOPFarmersMarketA

UNITS: Rs

DTQuantityWeightNonLOOP = IF SumNonLOOPQuantity= 0 THEN 0 ELSE NonLOOPDTQuantity/SumNonLOOPQuantity

UNITS: Dimensionless

DTWeightedPriceNonLOOP = DTQuantityWeightNonLOOP*DistanceTrader.QualityAdjDTBuyingPrice

UNITS: Rs/kg

LTQuantityWeightNonLOOP = IF SumNonLOOPQuantity = 0 THEN 0 ELSE NonLOOPLTQuantity/SumNonLOOPQuantity

UNITS: Dimensionless

LTWeightedPriceNonLOOP = LocalTrader.QualityAdjLTBuyingPrice*LTQuantityWeightNonLOOP

UNITS: Rs/kg

MarketACostsPerNonLOOPFarmer = TotalMarketANonLOOPCosts/NonLOOPMarketable.NonLOOPFarmersMarketA

UNITS: Rs

MarketAPropSoldNonLOOP = IF NonLOOPMarketable.TotalNonLOOPMarketA = 0 THEN 0 ELSE (NonLOOPRetailerQuantity+NonLOOPLTQuantity+NonLOOPDTQuantity)/ NonLOOPMarketable.TotalNonLOOPMarketA

UNITS: Dimensionless

MarketAPropUnsoldNonLOOP = 1-MarketAPropSoldNonLOOP

UNITS: Dimensionless

MarketAWeightedAveragePriceNonLOOP = DTWeightedPriceNonLOOP+ LTWeightedPriceNonLOOP+RetailerWeightedPriceNonLOOP

UNITS: Rs/kg

NonLOOPCostPerKg = RANDOM(1, 1.5, 45)

UNITS: Rs/kg

NonLOOPDTQuantity = DistanceTrader.DTNonLOOPSold

UNITS: kg

NonLOOPLTQuantity = LocalTrader.LTNonLOOPSold

UNITS: kg

NonLOOPRetailerQuantity = LocalRetailer.NonLOOPRetailerSold

UNITS: kg

RetailerQuantityWeightNonLOOP = IF SumNonLOOPQuantity = 0 THEN 0 ELSE NonLOOPRetailerQuantity/SumNonLOOPQuantity

UNITS: Dimensionless

RetailerWeightedPriceNonLOOP = LocalRetailer.QualityAdjRetBuyingPrice* RetailerQuantityWeightNonLOOP

UNITS: Rs/kg

SumNonLOOPQuantity = NonLOOPDTQuantity+NonLOOPLTQuantity+NonLOOPRetailerQuantity

UNITS: kg

TotalMarketANonLOOPCosts = TotalTransportCostNonLOOP+TotalNonLOOPCommissionMarketA

UNITS: Rs

TotalNonLOOPCommissionMarketA = GaddidarCommission.DTNonLOOPCommissionIncurred+ GaddidarCommission.LTNonLOOPCommissionIncurred+GaddidarCommission.RetailerNonLOOPCommissionIncurred

UNITS: Rs

TotalNonLoopRevenueMarketA = DistanceTrader.DTNonLOOPRevenue+ LocalTrader.LTNonLOOPRevenue+LocalRetailer.RetailNonLOOPRevenue

UNITS: Rs

TotalTransportCostNonLOOP = SumNonLOOPQuantity*NonLOOPCostPerKg

UNITS: Rs

NonLoop Benefits Market B

AfternoonNonLOOPYieldChange(t) = AfternoonNonLOOPYieldChange(t - dt) + (AfternoonNonLOOPYieldChangeFlow) * dt

INIT AfternoonNonLOOPYieldChange = 1

UNITS: Dimensionless

INFLOWS:

AfternoonNonLOOPYieldChangeFlow = AfternoonNLInvestmentYieldRate

UNITS: Dimensionless/halfday

CumulativeNonLOOPProfitPerFarmerAfternoon(t) = CumulativeNonLOOPProfitPerFarmerAfternoon(t - dt) + (ChangeCumulativeNonLOOPProfitAfternoon - NonLOOPLandInvestmentAfternoon - NonLOOPYieldInvestAfternoon) * dt

INIT CumulativeNonLOOPProfitPerFarmerAfternoon = 0

UNITS: Rs

INFLOWS:

ChangeCumulativeNonLOOPProfitAfternoon = IF LOOPMarketable.MorningIndicator = 0 THEN WeightedNonLOOPProfitPerFarmer/FarmerPopulationAndAdoption.HarvestFrequency ELSE 0

UNITS: Rs/halfday

OUTFLOWS:

NonLOOPLandInvestmentAfternoon = IF SeasonChange = 0 OR CumulativeNonLOOPProfitPerFarmerAfternoon < PriceTenthKatha/NonLOOPLandInvestmentRateAfternoon OR SeasonalSmoothProfits < 0 THEN 0 ELSE CumulativeNonLOOPProfitPerFarmerAfternoon*NonLOOPLandInvestmentRateAfternoon

UNITS: Rs/halfday

NonLOOPYieldInvestAfternoon = IF SeasonChange = 0 OR CumulativeNonLOOPProfitPerFarmerAfternoon <0 THEN 0 ELSE CumulativeNonLOOPProfitPerFarmerAfternoon*NonLOOPYieldInvestmentRateAfternoon

UNITS: Rs/halfday

CumulativeNonLOOPProfitPerFarmerMorning(t) = CumulativeNonLOOPProfitPerFarmerMorning(t - dt) + (ChangeCumulativeNonLOOPProfitMorn - NonLOOPLandInvestmentMorning - NonLOOPYieldInvestMorning) * dt

INIT CumulativeNonLOOPProfitPerFarmerMorning = 0

UNITS: Rs

INFLOWS:

ChangeCumulativeNonLOOPProfitMorn = IF LOOPMarketable.MorningIndicator = 1 THEN WeightedNonLOOPProfitPerFarmer/(FarmerPopulationAndAdoption.HarvestFrequency) ELSE 0

UNITS: Rs/halfday

OUTFLOWS:

NonLOOPLandInvestmentMorning = IF SeasonChange = 0 OR CumulativeNonLOOPProfitPerFarmerMorning < PriceTenthKatha/NonLOOPLandInvestmentRateMorn OR SeasonalSmoothProfits < 0 THEN 0 ELSE CumulativeNonLOOPProfitPerFarmerMorning*NonLOOPLandInvestmentRateMorn

UNITS: Rs/halfday

NonLOOPYieldInvestMorning = IF SeasonChange = 0 OR CumulativeNonLOOPProfitPerFarmerMorning <0 THEN 0 ELSE CumulativeNonLOOPProfitPerFarmerMorning*NonLOOPYieldInvestmentRateMorn

UNITS: Rs/halfday

MorningNonLOOPYieldChange(t) = MorningNonLOOPYieldChange(t - dt) + (MorningNonLOOPYieldChangeFlow) * dt

INIT MorningNonLOOPYieldChange = 1

UNITS: Dimensionless

INFLOWS:

MorningNonLOOPYieldChangeFlow = MorningNLInvestmentYieldRate

UNITS: Dimensionless/halfday

AfternoonNLInvestmentYieldRate = GRAPH(NonLOOPYieldInvestAfternoon)

(0, 0.000), (20000, 0.010)

UNITS: Dimensionless

AverageNonLOOPCumulativeProfit = MEAN(CumulativeNonLOOPProfitPerFarmerMorning, CumulativeNonLOOPProfitPerFarmerAfternoon)

UNITS: Rs

AverageProfitPerNonLOOPFarmer = AverageRevenuePerNonLOOPFarmer-MarketBCostsPerNonLOOPFarmer

UNITS: Rs

AverageRevenuePerNonLOOPFarmer = TotalNonLoopRevenueMarketB/NonLOOPMarketable.NonLOOPFarmersMarketB

UNITS: Rs

HalfDaysPerSeason = GRAPH(TIME) (0, 302.0), (302, 184.0), (486, 244.0), (730, 302.0), (1032, 184.0), (1216, 244.0), (1460, 302.0), (1762, 184.0), (1946, 244.0), (2190, 302.0), (2492, 184.0), (2676, 244.0), (2978, 302.0)

UNITS: Halfdays

LTQuantityWeightNonLOOP = IF SumNonLOOPQuantityMarketB = 0 THEN 0 ELSE NonLOOPLTQuantity/SumNonLOOPQuantityMarketB

UNITS: Dimensionless

LTWeightedPriceNonLOOP = MarketBLocalTrader.QualityAdjLTBBuyingPrice*LTQuantityWeightNonLOOP

UNITS: Rs/kg

MarketBCostsPerNonLOOPFarmer = TotalMarketBNonLOOPCosts/NonLOOPMarketable.NonLOOPFarmersMarketB

UNITS: Rs

MarketBWeightedAveragePriceNonLOOP = LTWeightedPriceNonLOOP+RetailerWeightedPriceNonLOOP

UNITS: Rs/kg

MeanNonLOOPYieldChange = MEAN(MorningNonLOOPYieldChange, AfternoonNonLOOPYieldChange)

UNITS: Dimensionless

MorningNLInvestmentYieldRate = GRAPH(NonLOOPYieldInvestMorning)

(0, 0.000), (20000, 0.010)

UNITS: Dimensionless

NonLOOPLandExpansion = MEAN(NonLOOPLandInvestmentAfternoon, NonLOOPLandInvestmentMorning)/PriceTenthKatha

UNITS: katha

NonLOOPLandInvestmentRateAfternoon = 2/10

UNITS: Dimensionless

NonLOOPLandInvestmentRateMorn = 2/10

UNITS: Dimensionless

NonLOOPLTQuantity = MarketBLocalTrader.MarketBNonLOOPLTSold

UNITS: kg

NonLOOPRetailerQuantity = MarketBLocalRetailer.MarktBNonLOOPRetailerSold

UNITS: kg

NonLOOPPropSoldMarketB = IF NonLOOPMarketable.TotalNonLOOPMarketB= 0 THEN 0 ELSE (NonLOOPRetailerQuantity+NonLOOPLTQuantity)/NonLOOPMarketable.TotalNonLOOPMarketB

UNITS: Dimensionless

NonLOOPPropUnsoldMarketB = 1- NonLOOPPropSoldMarketB

UNITS: Dimensionless

NonLOOPYieldInvestmentRateAfternoon = 1/10

UNITS: Dimensionless

NonLOOPYieldInvestmentRateMorn = 1/10

UNITS: Dimensionless

PriceTenthKatha = 76000*0.1

UNITS: Rs

RetailerQuantityWeightNonLOOP = IF SumNonLOOPQuantityMarketB = 0 THEN 0 ELSE NonLOOPRetailerQuantity/SumNonLOOPQuantityMarketB

UNITS: Dimensionless

RetailerWeightedPriceNonLOOP = MarketBLocalRetailer.QualityAdjRetBBuyingPrice*RetailerQuantityWeightNonLOOP

UNITS: Rs/kg

SeasonalSmoothProfits = SMTH1(WeightedNonLOOPProfitPerFarmer-DELAY(WeightedNonLOOPProfitPerFarmer, HalfDaysPerSeason), HalfDaysPerSeason)

UNITS: Rs

SeasonChange = LOOPLand&Yield.HalfDaySeason-DELAY(LOOPLand&Yield.HalfDaySeason, 1)

UNITS: Dimensionless

SumNonLOOPQuantityMarketB = NonLOOPLTQuantity+NonLOOPRetailerQuantity

UNITS: kg

TotalMarketBNonLOOPCosts = TotalTransportCostMarketBNonLOOP+ TotalNonLOOPCommissionMarketB

UNITS: Rs

TotalNonLOOPCommissionMarketB = MarketBGaddidarCommission.MarketBLTNonLOOPCommissionIncurred+ MarketBGaddidarCommission.MarketBRetailerNonLOOPCommissionIncurred

UNITS: Rs

TotalNonLoopRevenueMarketB = MarketBLocalTrader.MarketBLTNonLOOPRevenue+ MarketBLocalRetailer.MarketBRetailNonLOOPRevenue

UNITS: Rs

TotalNonLOOPRevenues = TotalNonLoopRevenueMarketB+ NonLOOPBenefitsMarketA.TotalNonLoopRevenueMarketA

UNITS: Rs

TotalNonLOOPSales = SumNonLOOPQuantityMarketB+NonLOOPBenefitsMarketA.SumNonLOOPQuantity

UNITS: kg

TotalNonLOOPUnsoldWeighted = WeightedUnsoldMarketB+WeightedUnsoldMarketA

UNITS: Dimensionless

TotalTransportCostMarketBNonLOOP = SumNonLOOPQuantityMarketB*NonLOOPBenefitsMarketA.NonLOOPCostPerKg

UNITS: Rs

WeightedNonLOOPPrice = IF NonLOOPMarketable.TotalNonLOOPMarketA = 0 AND SumNonLOOPQuantityMarketB = 0 THEN 0 ELSE (NonLOOPBenefitsMarketA.MarketAWeightedAveragePriceNonLOOP*(NonLOOPMarketable.TotalNonLOOPMarketA/TotalNonLOOPSales))+(MarketBWeightedAveragePriceNonLOOP*(SumNonLOOPQuantityMarketB/TotalNonLOOPSales))

UNITS: Rs/kg

WeightedNonLOOPProfitMarketA = IF TotalNonLOOPSales = 0 THEN 0 ELSE NonLOOPBenefitsMarketA.AverageProfitPerNonLOOPFarmer*( NonLOOPBenefitsMarketA.SumNonLOOPQuantity/TotalNonLOOPSales)

UNITS: Rs

WeightedNonLOOPProfitMarketB = IF TotalNonLOOPSales = 0 THEN 0 ELSE AverageProfitPerNonLOOPFarmer*(SumNonLOOPQuantityMarketB/TotalNonLOOPSales)

UNITS: Rs

WeightedNonLOOPProfitPerFarmer = (WeightedNonLOOPProfitMarketB+ WeightedNonLOOPProfitMarketA)-OnfarmCosts.NLTotalHalfDayCosts

UNITS: Rs

WeightedUnsoldMarketA = IF (NonLOOPMarketable.TotalNonLOOPMarketA+NonLOOPMarketable.TotalNonLOOPMarketB) = 0 THEN 0 ELSE NonLOOPBenefitsMarketA.MarketAPropUnsoldNonLOOP* (NonLOOPMarketable.TotalNonLOOPMarketA/(NonLOOPMarketable.TotalNonLOOPMarketA+NonLOOPMarketable.TotalNonLOOPMarketB))

UNITS: Dimensionless

WeightedUnsoldMarketB = IF (NonLOOPMarketable.TotalNonLOOPMarketB+NonLOOPMarketable.TotalNonLOOPMarketA) = 0 THEN 0 ELSE NonLOOPUnsoldMarketB*( NonLOOPMarketable.TotalNonLOOPMarketB/ (NonLOOPMarketable.TotalNonLOOPMarketA+NonLOOPMarketable.TotalNonLOOPMarketB))

UNITS: Dimensionless

F.6 Production & Aggregation

Loop Land & Yield

FVAreaProportion(t) = FVAreaProportion(t - dt) + (FVAreaPropChange) * dt

INIT FVAreaProportion = 0.7

UNITS: Dimensionless

INFLOWS:

FVAreaPropChange = IF TwoSeasonMaxMinModel = 0 THEN -0.00055*LOOPStapleFavourable ELSE 0.00055*LOOPSmoothStapleIndex

UNITS: Dimensionless/halfdays

LOOPArea(t) = LOOPArea(t - dt) + (BenefitsMarketB.LOOPLandExpansion) * dt

INIT LOOPArea = 45

UNITS: katha

INFLOWS:

BenefitsMarketB.LOOPLandExpansion = LOOPLandInvestment / NonLOOPBenefitsMarketB.PriceTenthKatha

UNITS: katha

AdjustedYield = BenefitsMarketB.LOOPYieldChange*(BaselineYield*FarmerPopulationAndAdoption.HarvestFrequency)

UNITS: kg/katha

BaselineYield = GRAPH(TIME) (1, 0.16), (152, 0.17), (394, 0.11), (610, 0.12), (882, 0.17), (1124, 0.11), (1340, 0.12), (1612, 0.17), (1854, 0.11), (2070, 0.12), (2342, 0.17), (2584, 0.11), (2800, 0.12), (3072, 0.17)

UNITS: kg/katha/halfday

FVAreaProportionUse = IF FVAreaProportion > 1 THEN 1 ELSE FVAreaProportion

UNITS: Dimensionless

FVPriceFix = IF BenefitsMarketB.WeightedMeanLOOPPrice = 0 THEN NonLOOPBenefitsMarketB.WeightedNonLOOPPrice ELSE BenefitsMarketB.WeightedMeanLOOPPrice

UNITS: Rs/kg

HalfDaySeason = GRAPH(TIME) (0, 0.0), (302, 1.0), (486, 2.0), (730, 0.0), (1032, 1.0), (1216, 2.0), (1460, 0.0), (1762, 1.0), (1946, 2.0), (2190, 0.0), (2492, 1.0), (2676, 2.0), (2920, 0.0), (3222, 1.0)

UNITS: Halfdays

LOOPLOOPFarmersToday = FarmerPopulationAndAdoption.LOOPFarmersSupplyingAnyGivenDay*LOOPProportionLOOP

UNITS: People

LOOPLOOPProduction = LOOPProduction*LOOPProportionLOOP

UNITS: kg

LOOPProduction = LOOPArea*FVAreaProportionUse*AdjustedYield*FarmerPopulationAndAdoption.LOOPFarmersSupplyingAnyGivenDay

UNITS: kg/halfday

LOOPProportionLOOP = IF 1-LOOPRandomSelfSupply-(1-ShortTermSatisfaction.LOOPShortTermSatisfactionUSE) > 1 THEN 1 ELSE 1-LOOPRandomSelfSupply-(1-ShortTermSatisfaction.LOOPShortTermSatisfactionUSE)

UNITS: Dimensionless

LOOPQualitySmoothTime = 14

UNITS: halfdays

LOOPRandomSelfSupply = RANDOM(0, 0.1, 2)

UNITS: Dimensionless

LOOPRelativeFVStapleIndex = FVPriceFix/StaplePrice

UNITS: Dimensionless

LOOPSmoothStapleIndex = SMTH1(LOOPRelativeFVStapleIndex, StaplePricePerceptionTime)

UNITS: Rs/kg

LOOPStapleFavourable = IF LOOPSmoothStapleIndex < 1 THEN 1+(1-LOOPSmoothStapleIndex) ELSE 1-(LOOPSmoothStapleIndex-1)

UNITS: Dimensionless

LOOPSupplyQuality = RANDOM(MinLOOPQuality, MaxLOOPQuality, 1)

UNITS: Dimensionless

MaxLOOPQuality = 1

UNITS: Dimensionless

MinLOOPQuality = 0.6

UNITS: Dimensionless

NonLOOPLOOPFarmersToday = FarmerPopulationAndAdoption.LOOPFarmersSupplyingAnyGivenDay*(1-LOOPProportionLOOP)

UNITS: People

NonLOOPLOOPProduction = LOOPProduction*(1-LOOPProportionLOOP)

UNITS: kg

SmoothLOOPSupplyQuality = SMTH1(LOOPSupplyQuality, LOOPQualitySmoothTime)

UNITS: Dimensionless

StaplePrice = GRAPH(TIME)

(1, 16.67), (63, 16.63), (123, 16.40), (185, 16.13), (247, 16.33), (303, 16.06), (365, 15.93), (425, 15.73), (487, 15.63), (547, 15.70), (609, 16.93), (671, 16.77), (731, 16.50), (793, 16.80), (853, 17.07), (915, 16.13), (977, 16.33), (1033, 16.07), (1095, 15.93), (1155, 15.73), (1217, 15.63), (1277, 15.70), (1339, 16.93), (1401, 16.77), (1461, 16.50), (1523, 16.80), (1583, 17.07), (1645, 16.13), (1707, 16.33), (1763, 16.07), (1825, 15.93), (1885, 15.73), (1947, 15.63), (2007, 15.70), (2069, 16.93), (2131, 16.77), (2191, 16.50), (2253, 16.80), (2313, 17.07), (2375, 16.13), (2437, 16.33), (2493, 16.07), (2555, 15.93), (2615, 15.73), (2677, 15.63), (2737, 15.70), (2799, 16.93), (2861, 16.77), (2921, 16.5)

UNITS: Rs/kg

StaplePricePerceptionTime = 240

UNITS: halfdays

TwoSeasonMaxMinModel = GRAPH(TIME) (0, 1.000), (150, 0.000), (514, 1.000), (882, 0.000), (1246, 1.000), (1614, 0.000), (1978, 1.000), (2346, 0.000), (2710, 1.000), (3078, 0.000)

UNITS: Dimensionless

***Loop Marketable***

LOOPMarketableVolume(t) = LOOPMarketableVolume(t - dt) + (LOOPLand&Yield.LOOPLOOPProduction - MarketADistanceTraderLOOP - MarketALocalLOOP - MarketARetailLOOP - MarketBLocalLOOP - MarketBRetailLOOP - LOOPWasted - LOOPHomeConsumption - LOOPGivenAway) * dt

INIT LOOPMarketableVolume = 0

UNITS: kg

INFLOWS:

Flow1 =

UNITS: kg

OUTFLOWS:

MarketADistanceTraderLOOP = PULSE(LOOPMarketableVolume* MarketPreference.MarketAPreferenceOverBUse*TraderPreference.LOOPDistanceTraderSupplyPreferenceUSE, 1, 2)

UNITS: kg/halfdays

MarketALocalLOOP = PULSE(LOOPMarketableVolume* MarketPreference.MarketAPreferenceOverBUse*TraderPreference.LOOPMorningLTSupplyPreferenceUSE, 1, 2)

UNITS: kg/halfdays

MarketARetailLOOP = PULSE(LOOPMarketableVolume*TraderPreference.LOOPMorningRetailerSupplyPrefUSE*MarketPreference.MarketAPreferenceOverBUse, 1, 2)

UNITS: kg/halfdays

MarketBLocalLOOP = PULSE(LOOPMarketableVolume*(1-MarketPreference.MarketAPreferenceOverBUse)*MarketBWhichTrader.NonLOOPMarketBLTPreferenceUSE, 1, 2)

UNITS: kg/halfdays

MarketBRetailLOOP = PULSE(LOOPMarketableVolume*(1-MarketPreference.MarketAPreferenceOverBUse)*(1-MarketBWhichTrader.NonLOOPMarketBLTPreferenceUSE), 1, 2)

UNITS: kg/halfdays

LOOPWasted = OnFarmWastageRate*LOOPMarketableVolume

UNITS: kg/halfdays

LOOPHomeConsumption = HomeConsumption.LOOPF&VDemand

UNITS: kg/halfdays

LOOPGivenAway = LOOPGivenAwayRate*LOOPMarketableVolume

UNITS: kg/halfdays

LOOPFarmersToday = LOOPMarketAFarmers+LOOPMarketBFarmers

UNITS: People

LOOPGivenAwayRate = 0.025

UNITS: Dimensionless

LOOPMarketAFarmers = LOOPLand&Yield.LOOPLOOPFarmersToday*MarketPreference.MarketAPreferenceOverBUse

UNITS: People

LOOPMarketAPerFarmer = IF LOOPLand&Yield.LOOPLOOPFarmersToday = 0 OR MarketPreference.MarketAPreferenceOverBUse = 0 THEN 0 ELSE TotalMarketALOOP/(LOOPLand&Yield.LOOPLOOPFarmersToday*MarketPreference.MarketAPreferenceOverBUse)

UNITS: kg

LOOPMarketBFarmers = LOOPLand&Yield.LOOPLOOPFarmersToday*(1-MarketPreference.MarketAPreferenceOverBUse)

UNITS: People

LOOPMarketBPerFarmer = IF LOOPLand&Yield.LOOPLOOPFarmersToday = 0 OR MarketPreference.MarketAPreferenceOverBUse = 1 THEN 0 ELSE TotalMarketBLOOP/(LOOPLand&Yield.LOOPLOOPFarmersToday*(1-MarketPreference.MarketAPreferenceOverBUse))

UNITS: kg

MorningIndicator = GRAPH(TIME) [repeat ‘1’ and ‘0’ total of 1460 times, where ‘1’ equals *morning* and ‘0’ equals *non-morning* (i.e. afternoon)]

UNITS: Dimensionless

OnFarmWastageRate = 0.045

UNITS: Dimensionless

TotalLOOPAggregation = TotalMarketBLOOP+TotalMarketALOOP

UNITS: kg

TotalMarketALOOP = MarketARetailLOOP+MarketALocalLOOP+MarketADistanceTraderLOOP

UNITS: kg

TotalMarketBLOOP = MarketBRetailLOOP+MarketBLocalLOOP

UNITS: kg

***Market Preference***

MarketAPreferenceOverB(t) = MarketAPreferenceOverB(t - dt) + (MarketAPreferenceChange) * dt

INIT MarketAPreferenceOverB = 1

UNITS: Dimensionless

INFLOWS:

MarketAPreferenceChange = ((MarketSalesWeight*MarketASoldOverB+MarketProfitWeight*MarketAPotentialProfitsOverB)+RandomPrefChange)/MarketPreferenceChangeTime

UNITS: Dimensionless/halfdays

AggregatorCapacitySD = 900

UNITS: kg

AggregatorDailyCapacity = SMTH1(NORMAL(AggregatorDefaultCapacity, AggregatorCapacitySD, 2), SmoothCapacityTime)

UNITS: kg

AggregatorDefaultCapacity = 2000

UNITS: kg

AggregatorNumbersMarketA = LOOPMarketable.TotalLOOPAggregation/AggregatorDailyCapacity

UNITS: People

AggregatorNumbersMarketB = LOOPMarketable.TotalLOOPAggregation/AggregatorDailyCapacity

UNITS: People

MarketACostPerKm = 140

UNITS: Rs/km

MarketADistance = 10

UNITS: km

MarketAPotentialCommission = LOOPMarketable.TotalLOOPAggregation* GaddidarCommission.GaddidarLocalActorCommission

UNITS: Rs

MarketAPotentialPerFarmerProfit = IF LOOPLand&Yield.LOOPLOOPFarmersToday = 0 THEN 0 ELSE MarketAPotentialProfit/LOOPLand&Yield.LOOPLOOPFarmersToday

UNITS: Rs

MarketAPotentialProfit = MarketAPotentialRevenue-MarketATransportCost-MarketAPotentialCommission

UNITS: Rs

MarketAPotentialProfitsOverB = IF MarketBPotentialProfitPerFarmer = 0 AND MarketAPotentialPerFarmerProfit = 0 THEN 0 ELSE (MarketAPotentialPerFarmerProfit/ (MarketAPotentialPerFarmerProfit+MarketBPotentialProfitPerFarmer))-0.5

UNITS: Dimensionless

MarketAPotentialRevenue = SMTH1(DELAY1(GaddidarCommission.MarketAMeanWeightedPrice, 2), 14) *LOOPMarketable.TotalLOOPAggregation

UNITS: Rs

MarketAPreferenceOverBUse = IF MarketAPreferenceOverB > 1 THEN 1 ELSE DELAY1(MarketAPreferenceOverB, 2)

UNITS: Dimensionless

MarketASingleVehicleCost = MarketACostPerKm*MarketADistance*(1-MarketATransportSubsidy)

UNITS: Rs

MarketASoldOverB = IF BenefitsMarketB.MarketBLOOPSold = 0 AND BenefitsMarketA.MarketALOOPSoldUse = 0 THEN 0 ELSE DELAY((BenefitsMarketA.MarketALOOPSoldUse/(BenefitsMarketA.MarketALOOPSoldUse+BenefitsMarketB.MarketBLOOPSold)) -0.5, 2)

UNITS: Dimensionless

MarketATransportCost = MarketASingleVehicleCost*AggregatorNumbersMarketA

UNITS: Rs

MarketATransportSubsidy = GRAPH(TIME) (1.0, 0.500), (730.0, 0.000)

UNITS: Dimensionless

MarketBCostPerKm = 180

UNITS: Rs/km

MarketBDistance = 10

UNITS: km

MarketBPotentialProfit = MarketBPotentialRevenue - MarketBTransportCost

UNITS: Rs

MarketBPotentialProfitPerFarmer = IF LOOPLand&Yield.LOOPLOOPFarmersToday = 0 THEN 0 ELSE MarketBPotentialProfit/LOOPLand&Yield.LOOPLOOPFarmersToday

UNITS: Rs

MarketBPotentialRevenue = SMTH1(DELAY1(MarketBGaddidarCommission.MarketBMeanWeightedPrice, 2), 14)*LOOPMarketable.TotalLOOPAggregation

UNITS: Rs

MarketBSingleVehicleCost = IF TIME < 669 THEN MarketBCostPerKm*MarketBDistance*(1-MarketBTransportSubsidy) ELSE MarketBCostPerKm*MarketBDistance*(1-MarketBSubsidyScenario)

UNITS: Rs

MarketBSubsidyScenario = IF TIME < 669 THEN 0 ELSE "A.ScenarioHub".MarketBSubsidySCENARIO

UNITS: Dimensionless

MarketBTransportCost = MarketBSingleVehicleCost*AggregatorNumbersMarketB

UNITS: Rs

MarketBTransportSubsidy = GRAPH(TIME) (1.0, 0.500), (730.0, 0.000)

UNITS: Dimensionless

MarketPreferenceChangeTime = 14

UNITS: halfdays

MarketProfitWeight = 0.1

UNITS: Dimensionless

MarketSalesWeight = 0.2

UNITS: Dimensionless

RandomPrefChange = RANDOM(-0.05, 0.05, 10)

UNITS: Dimensionless

SmoothCapacityTime = 60

UNITS: halfdays

**Non-Loop Land & Yield**

LOOPF&VArea(t) = LOOPF&VArea(t - dt) + (NonLOOPBenefitsMarketB.NonLOOPLandExpansion) * dt

INIT LOOPF&VArea = 38

UNITS: katha

INFLOWS: NLLandAreaChange = NonLOOPBenefitsMarketB.NonLOOPLandExpansion

UNITS: katha

NonLOOPFVAreaProportion(t) = NonLOOPFVAreaProportion(t - dt) + (NonLOOPFVAreaPropChange) * dt

INIT NonLOOPFVAreaProportion = 0.7

UNITS: Dimensionless

INFLOWS:

NonLOOPFVAreaPropChange = IF TwoSeasonMaxMinModel = 0 THEN -0.00055*NonLOOPStapleFavourable ELSE 0.00055*NonLOOPSmoothStapleIndex

UNITS: Dimensionless/halfdays

MaxNonLOOPQuality = 1

UNITS: Dimensionless

MinNonLOOPQuality = 0.6

UNITS: Dimensionless

NonLoopAdjustedYield = NonLOOPBenefitsMarketB.MeanNonLOOPYieldChange*(LOOPLand&Yield.BaselineYield*FarmerPopulationAndAdoption.HarvestFrequency)

UNITS: kg/katha

NonLOOPFVAreaProportionUse = IF NonLOOPFVAreaProportion > 1 THEN 1 ELSE NonLOOPFVAreaProportion

UNITS: Dimensionless

NonLOOPProduction = LOOPF&VArea*NonLOOPFVAreaProportionUse*NonLoopAdjustedYield*FarmerPopulationAndAdoption.NonLOOPFarmersGivenDay

UNITS: kg/halfday

NonLOOPQualitySmoothTime = 14

UNITS: halfdays

NonLOOPRelativeFVStapleIndex = NonLOOPBenefitsMarketB.WeightedNonLOOPPrice/LOOPLand&Yield.StaplePrice

UNITS: Dimensionless

NonLOOPSmoothStapleIndex = SMTH1(NonLOOPRelativeFVStapleIndex, LOOPLand&Yield.StaplePricePerceptionTime)

UNITS: Rs/kg

NonLOOPStapleFavourable = IF NonLOOPSmoothStapleIndex < 1 THEN 1+(1-NonLOOPSmoothStapleIndex) ELSE 1-(NonLOOPSmoothStapleIndex-1)

UNITS: Dimensionless

NonLOOPSupplyQuality = RANDOM(MinNonLOOPQuality, MaxNonLOOPQuality, 28)

UNITS: Dimensionless

SmoothNonLOOPSupplyQuality = SMTH1(NonLOOPSupplyQuality, NonLOOPQualitySmoothTime)

UNITS: Dimensionless

TwoSeasonMaxMinModel = GRAPH(TIME) (0, 1.000), (150, 0.000), (514, 1.000), (882, 0.000), (1246, 1.000), (1614, 0.000), (1978, 1.000), (2346, 0.000), (2710, 1.000), (3078, 0.000)

UNITS: Dimensionless

**NonLoop Marketable**

NonLOOPMarketableF&V(t) = NonLOOPMarketableF&V(t - dt) + (NonLOOPLand&Yield.NonLOOPProduction + LOOPLand&Yield.NonLOOPLOOPProduction - NonLOOPHomeConsumption - MarketADistanceNonLOOP - MarketALocalNonLOOP - MarketARetailNonLOOP - MarketBLocalNonLOOP - MarketBRetailLOOP - NonLOOPGivenAway - NonLOOPWastage) * dt

INIT NonLOOPMarketableF&V = 0

UNITS: kg

INFLOWS:

NonLOOPLand&Yield.NonLOOPProduction = NLF&VArea*NonLOOPFVAreaProportionUSE*

NonLoopAdjustedYield*FarmerPopulationAndAdoption.NonLOOPFarmersGivenDay

UNITS: kg/halfdays

LOOPLand&Yield.NonLOOPLOOPProduction = LOOPProduction*(1-LOOPProportionLOOP)

UNITS: kg/halfdays

OUTFLOWS:

NonLOOPHomeConsumption = HomeConsumption.NonLOOPF&VDemand

UNITS: kg/halfdays

MarketADistanceNonLOOP = IF LOOPMarketable.TotalLOOPAggregation > 0 THEN NonLOOPMarketableF&V*NonLOOPMarketAProportion*TraderPreference.NonLOOPDTSupplyPreferenceUSE ELSE 0

UNITS: kg/halfdays

MarketALocalNonLOOP = IF LOOPMarketable.TotalLOOPAggregation >0 THEN NonLOOPMarketableF&V*NonLOOPMarketAProportion*TraderPreference.NonLOOPMorningLTSupplyPreferenceUSE ELSE NonLOOPMarketableF&V*NonLOOPMarketAProportion*TraderPreference.NonLOOPAfternoonLTSupplyPreferenceUSE

UNITS: kg/halfdays

MarketARetailNonLOOP = IF LOOPMarketable.TotalLOOPAggregation > 0 THEN NonLOOPMarketableF&V*TraderPreference.NonLOOPMorningRetailerPreferenceUSE*NonLOOPMarketAProportion ELSE NonLOOPMarketableF&V*NonLOOPMarketAProportion*TraderPreference.NonLOOPAfternoonRetailerSupplyPreferenceUSE

UNITS: kg/halfdays

MarketBLocalNonLOOP = NonLOOPMarketBProportion*MarketBWhichTrader.NonLOOPMarketBLTPreferenceUSE*NonLOOPMarketableF&V

UNITS: kg/halfdays

MarketBRetailLOOP = NonLOOPMarketableF&V*NonLOOPMarketBProportion*(1-MarketBWhichTrader.NonLOOPMarketBLTPreferenceUSE)

UNITS: kg/halfdays

NonLOOPGivenAway = NonLOOPGiveAwayRate*NonLOOPMarketableF&V

UNITS: kg/halfdays

NonLOOPWastage = NonLOOPWastageRate*NonLOOPMarketableF&V

UNITS: kg/halfdays

DefaultNonLoopMarketA = 0.9

UNITS: Dimensionless

NonLOOPFarmersMarketA = NonLOOPMarketAProportion*NonLOOPFarmersToday

UNITS: People

NonLOOPFarmersMarketB = NonLOOPMarketBProportion*NonLOOPFarmersToday

UNITS: People

NonLOOPFarmersToday = LOOPLand&Yield.NonLOOPLOOPFarmersToday+FarmerPopulationAndAdoption.NonLOOPFarmersGivenDay

UNITS: People

NonLOOPGiveAwayRate = 0.025

UNITS: Dimensionless

NonLOOPMarketAPerFarmer = (MarketADistanceNonLOOP+MarketARetailNonLOOP+MarketALocalNonLOOP)/NonLOOPFarmersMarketA

UNITS: kg

NonLoopMarketAProp = IF DefaultNonLoopMarketA*RANDOM(0.9, 1.1, 111) > 1 THEN 1 ELSE DefaultNonLoopMarketA*RANDOM(0.9, 1.1, 111)

UNITS: Dimensionless

NonLOOPMarketAProportion = NonLoopMarketAProp*NonLoopMarketAProp

UNITS: Dimensionless

NonLOOPMarketBPerFarmer = (MarketBLocalNonLOOP+MarketBRetailLOOP)/(NonLOOPFarmersMarketB*NonLOOPMarketBProportion)

UNITS: kg

NonLOOPMarketBProportion = 1-NonLoopMarketAProp

UNITS: Dimensionless

NonLOOPWastageRate = 0.045

UNITS: Dimensionless

TotalNonLOOPMarketA = MarketADistanceNonLOOP+MarketALocalNonLOOP+MarketARetailNonLOOP

UNITS: kg

TotalNonLOOPMarketB = MarketBRetailLOOP+MarketBLocalNonLOOP

UNITS: kg

Onfarm Costs

OnfarmCosts:

LabourDayMinimumWage = 257

UNITS: Rupees/day

LOOPFertilizer = LOGNORMAL(1600, 1080, 11)/YearToHalfDayConverter

UNITS: Rs/halfday

LOOPHalfDayFarmCosts = SMTH1(LOOPSeeds+LOOPFertilizer+LOOPPesticide+LOOPIrrigation+LOOPManure+LOOPTractor+LOOPRotavator, 60)

UNITS: Rs/halfday

LOOPHalfDayLabourCost = (LOOPPloughing+LOOPPlanting+LOOPHilling+LOOPWeeding+LOOPWatering+LOOPHarvesting+LOOPOthers)/YearToHalfDayConverter

UNITS: Rs/halfday

LOOPHarvesting = LOGNORMAL(2.8, 3.6, 26)*LabourDayMinimumWage

UNITS: Rs

LOOPHilling = LOGNORMAL(0.9, 1.5, 24)*LabourDayMinimumWage

UNITS: Rs

LOOPIrrigation = LOGNORMAL(2400, 2200, 13)/YearToHalfDayConverter

UNITS: Rs/halfday

LOOPManure = LOGNORMAL(80, 140, 14)/YearToHalfDayConverter

UNITS: Rs/halfday

LOOPOthers = 0*LabourDayMinimumWage

UNITS: Rs

LOOPPesticide = NORMAL(870, 490, 12)/YearToHalfDayConverter

UNITS: Rs/halfday

LOOPPlanting = LOGNORMAL(1.9, 2.7, 23)*LabourDayMinimumWage

UNITS: Rs

LOOPPloughing = LOGNORMAL(0.9, 1.4, 22)*LabourDayMinimumWage

UNITS: Rs

LOOPRotavator = LOGNORMAL(1200, 1200, 16)/YearToHalfDayConverter

UNITS: Rs/halfday

LOOPSeeds = LOGNORMAL(1100, 800, 10)/YearToHalfDayConverter

UNITS: Rs/halfday

LOOPTotalHalfDayCosts = LOOPHalfDayFarmCosts+LOOPHalfDayLabourCost

UNITS: Rs/halfday

LOOPTractor = LOGNORMAL(2100, 1900, 15)/YearToHalfDayConverter

UNITS: Rs/halfday

LOOPWatering = 0*LabourDayMinimumWage

UNITS: Rs

LOOPWeeding = LOGNORMAL(5.9, 7.8, 25)*LabourDayMinimumWage

UNITS: Rs

NLFertilizer = LOGNORMAL(1720, 1320, 4)/YearToHalfDayConverter

UNITS: Rs/halfday

NLHalfDayFarmCosts = SMTH1(NLSeeds+NLFertilizer+NLPesticide+NLIrrigation+NLManure+NLTractor+NLRotavator, 60)

UNITS: Rs/halfday

NLHalfDayLabourCost = (NLPloughing+NLPlanting+NLHilling+NLWeeding+NLWatering+NLHarvesting+NLOthers)/YearToHalfDayConverter

UNITS: Rs/halfday

NLHarvesting = LOGNORMAL(3.2, 4.8, 21)*LabourDayMinimumWage

UNITS: Rs

NLHilling = LOGNORMAL(1, 1.8, 19)*LabourDayMinimumWage

UNITS: Rs

NLIrrigation = LOGNORMAL(1450, 1290, 6)/YearToHalfDayConverter

UNITS: Rs/halfday

NLManure = LOGNORMAL(90, 140, 7)/YearToHalfDayConverter

UNITS: Rs/halfday

NLOthers = 0*LabourDayMinimumWage

UNITS: Rs

NLPesticide = LOGNORMAL(1110, 650, 5)/YearToHalfDayConverter

UNITS: Rs/halfday

NLPlanting = LOGNORMAL(2.5, 3.6, 18)*LabourDayMinimumWage

UNITS: Rs

NLPloughing = LOGNORMAL(0.3, 0.7, 17)*LabourDayMinimumWage

UNITS: Rs

NLRotavator = LOGNORMAL(1500, 1800, 9)/YearToHalfDayConverter

UNITS: Rs/halfday

NLSeeds = LOGNORMAL(1400, 1020, 3)/YearToHalfDayConverter

UNITS: Rs/halfday

NLTotalHalfDayCosts = NLHalfDayFarmCosts+NLHalfDayLabourCost

UNITS: Rs/halfday

NLTractor = LOGNORMAL(2600, 2500, 8)/YearToHalfDayConverter

UNITS: Rs/halfday

NLWatering = 0*LabourDayMinimumWage

UNITS: Rs

NLWeeding = LOGNORMAL(5.5, 7.1, 20)*LabourDayMinimumWage

UNITS: Rs

WeightedAverageHalfDayCosts = (NLTotalHalfDayCosts*(FarmerPopulationAndAdoption.NonLOOPFarmersKoilwarBlock/FarmerPopulationAndAdoption.KoilwarFarmingHouseholds))+(LOOPTotalHalfDayCosts/FarmerPopulationAndAdoption.KoilwarFarmingHouseholds)

UNITS: Rs/halfday

YearToHalfDayConverter = 730

UNITS: halfdays

Short-term Satisfaction

LOOPShortTermSatisfaction(t) = LOOPShortTermSatisfaction(t - dt) + (STSatisfactionIncrease) * dt

INIT LOOPShortTermSatisfaction = 1

UNITS: Dimensionless

INFLOWS:

STSatisfactionIncrease = LOOPRelativeSatisfaction/SatisfactionPerceptionTime

UNITS: Dimensionless/halfdays

LOOPRelativeSatisfaction = IF BenefitsMarketB.WeightedLOOPProfitPerFarmer = 0 THEN 0 ELSE (BenefitsMarketB.WeightedLOOPProfitPerFarmer/(SmoothedNonLOOPProfitPerFarmer+BenefitsMarketB.WeightedLOOPProfitPerFarmer))-0.5

UNITS: Dimensionless

LOOPShortTermSatisfactionUSE = IF LOOPShortTermSatisfaction >= 1 THEN 1 ELSE LOOPShortTermSatisfaction

UNITS: Dimensionless

SatisfactionPerceptionTime = 14

UNITS: halfdays

SmoothedNonLOOPProfitPerFarmer = SMTH1(NonLOOPBenefitsMarketB.WeightedNonLOOPProfitPerFarmer, 2)

UNITS: Rs

**F.7 Retail Demand**

MarketADemand

CumulMarketACustomerDemand(t) = CumulMarketACustomerDemand(t - dt) + (CumulMarketADemandFlow) * dt

INIT CumulMarketACustomerDemand = 0

UNITS: kg

INFLOWS:

CumulMarketADemandFlow = IF LocalTrader.LocalTraderDownstreamFlow =0 THEN 0 ELSE MarketAPerCustomerDemands*MarketACustomerRoutine

UNITS: kg/halfdays

MarketAPerCustomerDemands(t) = MarketAPerCustomerDemands(t - dt) + (AdjustmentCustomerDemand) * dt

INIT MarketAPerCustomerDemands = ReferenceConsumerDemand

UNITS: kg

INFLOWS:

AdjustmentCustomerDemand = (IndicatedIndustryDemand-MarketAPerCustomerDemands)/Demandadjustmentdelay

UNITS: kg/halfdays

ReferenceConsumerDemand(t) = ReferenceConsumerDemand(t - dt) + (ReferenceDemandGrowthRate) * dt

INIT ReferenceConsumerDemand = 3.4

UNITS: kg

INFLOWS:

ReferenceDemandGrowthRate = IF TIME < 669 THEN 0 ELSE ReferenceConsumerDemand*((((1+"A.ScenarioHub".ExternalRetailReferenceDemandSCENARIO/100)^(1/YearsToHalfDays))-1))

UNITS: kg/halfdays

CustomersPerRetailer = InitialRetailCustomersPerRetailer

UNITS: People/halfday

Demandadjustmentdelay = 14

UNITS: halfdays

DemandCurveSlope = (-ReferenceConsumerDemand* ReferenceIndustryDemandElasticity)/ReferencePrice

UNITS: Dimensionless

IndicatedIndustryDemand = MIN(MaximumConsumption,ReferenceConsumerDemand)* MAX(0,1+DemandCurveSlope*(LocalRetailer.RetailerSellingPrice -ReferencePrice)/ReferenceConsumerDemand)

UNITS: kg

InitialRetailCustomersPerRetailer = 25

UNITS: People/halfday

MarketACustomerRoutine = 1/7

UNITS: halfdays

MarketAPerRetailerDemands = MarketAPerCustomerDemands*CustomersPerRetailer

UNITS: kg

MaximumConsumption = 5

UNITS: kg/halfday

ReferenceIndustryDemandElasticity = 0.92

UNITS: Dimensionless

ReferencePrice = 25

UNITS: Rs/kg

YearsToHalfDays = 730

UNITS: halfdays

Market B Demand

CumulMarketBCustomerDemand(t) = CumulMarketBCustomerDemand(t - dt) + (CumulMarketBDemandFlow) * dt

INIT CumulMarketBCustomerDemand = 0

UNITS: kg

INFLOWS:

CumulMarketBDemandFlow = IF MarketBLocalRetailer.MarketBRetailerDownstreamFlow = 0 THEN 0 ELSE MarketBPerCustomerDemands*MarketBCustomerRoutine

UNITS: kg/halfdays

MarketBPerCustomerDemands(t) = MarketBPerCustomerDemands(t - dt) + (AdjustmentCustomerDemand) * dt

INIT MarketBPerCustomerDemands = MarketADemand.ReferenceConsumerDemand

UNITS: kg

INFLOWS:

AdjustmentCustomerDemand = (IndicatedIndustryDemand-MarketBPerCustomerDemands)/MarketBDemandAdjustmentDelay

UNITS: kg/halfdays

DemandCurveSlope = (-MarketADemand.ReferenceConsumerDemand* ReferenceIndustryDemandElasticity)/ReferencePrice

UNITS: Dimensionless

IndicatedIndustryDemand = MIN(MaximumConsumption,MarketADemand.ReferenceConsumerDemand)* MAX(0,1+DemandCurveSlope*(MarketBLocalRetailer.MarketBRetailerSellingPrice -ReferencePrice)/MarketADemand.ReferenceConsumerDemand)

UNITS: kg

MarketBCustomerRoutine = 1/7

UNITS: halfdays

MarketBDemandAdjustmentDelay = 14

UNITS: halfdays

MarketBPerRetailerDemands = MarketBPerCustomerDemands*MarketBRetailCustomers

UNITS: kg

MarketBRetailCustomers = 25

UNITS: People/halfday

MaximumConsumption = 5

UNITS: kg/halfday

ReferenceIndustryDemandElasticity = 0.88

UNITS: Dimensionless

ReferencePrice = 25

UNITS: Rs/kg

Supplementary references

Barlas, Y., 1989. Multiple tests for validation of system dynamics type of simulation models. European Journal of Operational Research 42, 59–87. https://doi.org/http://dx.doi.org/10.1016/0377-2217(89)90059-3

Chapman, A., Darby, S., 2016. Evaluating sustainable adaptation strategies for vulnerable mega-deltas using system dynamics modelling: Rice agriculture in the Mekong Delta’s An Giang Province, Vietnam. Science of The Total Environment 559, 326–338. https://doi.org/https://doi.org/10.1016/j.scitotenv.2016.02.162

Chichakly, K., 2010. Integration Methods and DT [WWW Document]. STELLA: Modelling Tips. URL https://blog.iseesystems.com/modeling-tips/integration-methods-and-dt/ (accessed 1.10.21).

ISEES, 2018. STELLA Architect: Systems Thinking for Education and Research.

NHB, 2015. Horticulture Crops Estimates for the Year 2013-14 and 2014-15 [WWW Document]. National Horticultural Board, Ministry of Agriculture & Farmer’s Welfare (GoI).

Schwaninger, M., Groesser, S., 2016. System Dynamics Modeling: Validation for Quality Assurance. pp. 1–20. https://doi.org/10.1007/978-3-642-27737-5_540-3

Seppelt, R., Richter, O., 2005. “It was an artefact not the result”: A note on systems dynamic model development tools. Environmental Modelling & Software 20, 1543–1548. https://doi.org/https://doi.org/10.1016/j.envsoft.2004.12.004

Sterman, J.D., 2000. Business Dynamics: Systems Thinking and Modeling for a Complex World. Irwin/McGraw-Hill, New York City, USA.

USDA, 2019. India: Grain and Feed Annual Report. Washington DC, USA.

Voinov, A., Shugart, H.H., 2013. ‘Integronsters’, integral and integrated modeling. Environmental Modelling & Software 39, 149–158. https://doi.org/https://doi.org/10.1016/j.envsoft.2012.05.014
